# Architecture of the Mouse Brain Synaptome

**DOI:** 10.1016/j.neuron.2018.07.007

**Published:** 2018-08-22

**Authors:** Fei Zhu, Mélissa Cizeron, Zhen Qiu, Ruth Benavides-Piccione, Maksym V. Kopanitsa, Nathan G. Skene, Babis Koniaris, Javier DeFelipe, Erik Fransén, Noboru H. Komiyama, Seth G.N. Grant

**Affiliations:** 1 Genes to Cognition Program, Centre for Clinical Brain Sciences, University of Edinburgh, Edinburgh EH16 4SB, UK; 2 UCL Institute of Neurology, Queen Square, WC1N 3BG London, UK; 3 Institut NeuroMyoGène, Université de Lyon, Université Claude Bernard Lyon 1, CNRS UMR-5310, INSERM U-1217, 69008 Lyon, France; 4 Instituto Cajal (CSIC) 28002 Madrid, Centro de Tecnología Biomédica (UPM) 28223 Madrid; 5 CIBERNED, ISCIII, 28031 Madrid, Spain; 6 Synome Ltd, Babraham Research Campus, Cambridge CB22 3AT, UK; 7 UK Dementia Research Institute, Imperial College London, London W12 0NN, UK; 8 Laboratory of Molecular Neurobiology, Department of Medical Biochemistry and Biophysics, Karolinska Institutet, 17177 Stockholm, Sweden; 9 Department of Computational Science and Technology, School of Electrical Engineering and Computer Science, KTH Royal Institute of Technology, 10044 Stockholm, Sweden

**Keywords:** synaptome, synaptomic, synapse diversity, synaptic proteins, synapse proteome, connectome, PSD95, SAP102, machine learning, mutation

## Abstract

Synapses are found in vast numbers in the brain and contain complex proteomes. We developed genetic labeling and imaging methods to examine synaptic proteins in individual excitatory synapses across all regions of the mouse brain. Synapse catalogs were generated from the molecular and morphological features of a billion synapses. Each synapse subtype showed a unique anatomical distribution, and each brain region showed a distinct signature of synapse subtypes. Whole-brain synaptome cartography revealed spatial architecture from dendritic to global systems levels and previously unknown anatomical features. Synaptome mapping of circuits showed correspondence between synapse diversity and structural and functional connectomes. Behaviorally relevant patterns of neuronal activity trigger spatiotemporal postsynaptic responses sensitive to the structure of synaptome maps. Areas controlling higher cognitive function contain the greatest synapse diversity, and mutations causing cognitive disorders reorganized synaptome maps. Synaptome technology and resources have wide-ranging application in studies of the normal and diseased brain.

## Highlights

• Single-synapse-resolution maps of postsynaptic proteins across the whole mouse brain
• Systematic catalogs and anatomical mapping of synapse types and subtypes
• Synapse diversity is organized into a synaptome map architecture
• Disease-relevant mutations reprogram synaptome maps

## Graphical Abstract

**Figure:**
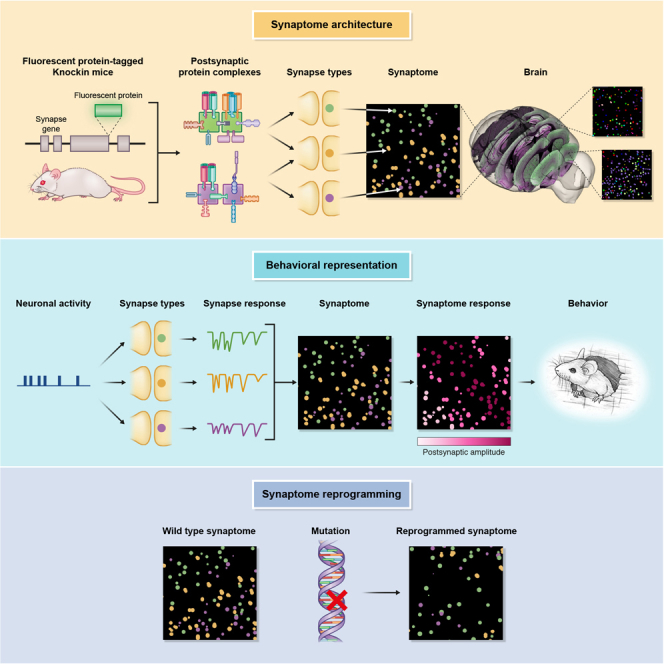

## Teaser

Synapse molecular composition and diversity was systematically mapped across the whole mouse brain. A molecular logic orchestrates the spatial architecture of synapse diversity from individual dendrites to the systems level. Synaptome architecture and function is important for cognition and disease.

## Introduction

The brain is the most complex organ, and a hallmark of this complexity is the vast number of synapses. Synapses are also highly complex at the molecular level, with >1,000 genes encoding postsynaptic proteins in excitatory synapses (Bayés et al., 2011, Bayés et al., 2012, Bayés et al., 2017, Collins et al., 2006, Distler et al., 2014, Emes et al., 2008, Husi et al., 2000, Peng et al., 2004, Roy et al., 2018, Trinidad et al., 2008, Uezu et al., 2016, Yoshimura et al., 2004). The differential expression of these proteins raises the possibility that there is high synapse diversity within the brain (Grant, 2007, Emes et al., 2008, O’Rourke et al., 2012). Although it is routine to examine the expression of proteins and the morphology of individual synapses in small tissue areas (using light and electron microscopy), there are no methods permitting the study of single-synapse molecular composition on the scale of the whole brain. As a result, the extent and spatial organization of synapse diversity across the brain are poorly understood. The term synaptome describes the set of all synapses in the brain, and to date, there has not been a single-synapse-resolution molecular map of the nervous system in any organism.

Synapses connect axons and dendrites into a global anatomical network that is often referred to as the structural connectome, whereas the functional connectome describes the activity of this network (Bullmore and Sporns, 2009). Synaptome mapping could be used to ask if the spatial distribution of synapses with different proteomes is related to connectome architecture. If so, this would support a fundamental role for synapse diversity in the specification of connections and systems-level organization and function. Synaptome mapping at single-synapse resolution could also increase the anatomical resolution of the connection matrix between brain regions.

Synapse diversity might be important for cognitive function. The prevailing model explaining how cognitive processes are represented in the brain is one in which each behavior is the product of a circuit or ensemble of neurons—the connectionist model. If circuits comprised diverse synapse types, arising from the differential distribution of proteins controlling synapse physiology, then this would result in functional diversity where each synapse type would respond differently to patterns of neural activity and thereby shape the circuit activity. Thus, synapse diversity could be an important mechanism for representing information within the brain. Identifying the synapses that express disease-relevant proteins will also be important for understanding how the more than 130 diseases arising from mutations in postsynaptic proteins cause their phenotypes (Bayés et al., 2011). Mutations that reorganized synapse diversity could result in changes to circuit function and representations.

We developed methods to capture information on the molecular composition and morphology of individual synapses on the scale of the whole mouse brain. From these data, we generated the first synaptome molecular maps of any organism. The approach utilizes mice expressing fluorescently labeled postsynaptic proteins and a semi-automated standardized image capture and analysis suite. We focused on two postsynaptic proteins expressed at excitatory synapses, PSD95 (Postsynaptic Density 95) and SAP102 (Synapse-Associated Protein 102), for the following reasons. First, we have extensive experience in genome engineering of these proteins in mice (Broadhead et al., 2016, Cuthbert et al., 2007, Fernández et al., 2009, Migaud et al., 1998) and now report the creation of two lines in which endogenous PSD95 is fused with eGFP (enhanced green fluorescent protein) and endogenous SAP102 with monomeric Kusabira Orange 2 (mKO2). In double-homozygous knockin mice, all copies of these proteins are labeled, which enables their visualization and quantification in individual postsynaptic puncta throughout the brain. Second, PSD95 and SAP102 are abundant and stable postsynaptic scaffolding proteins, which assemble neurotransmitter receptors, ion channels, and structural and signaling proteins into multiprotein signaling complexes (Broadhead et al., 2016, Cuthbert et al., 2007, Fernández et al., 2009, Fernández et al., 2017, Frank et al., 2016, Frank et al., 2017, Husi and Grant, 2001, Husi et al., 2000, Migaud et al., 1998). PSD95 and SAP102 are assembled into physically distinct complexes (Frank et al., 2016), and thus imaging of these proteins reveals the synaptic localization of these complexes. Third, these proteins have distinct roles in shaping synaptic responses to neural activity (Carlisle et al., 2008, Cuthbert et al., 2007, Elias et al., 2006, Migaud et al., 1998). Fourth, mutations in genes encoding these proteins result in cognitive impairments in mice (Cuthbert et al., 2007, Horner et al., 2018, Migaud et al., 1998, Nithianantharajah et al., 2013) and humans (Lelieveld et al., 2016, Tarpey et al., 2004, Wang et al., 2016, Xing et al., 2016).

Here, we report that synapse diversity arising from the differential distribution of postsynaptic proteins generates a previously unanticipated synaptome architecture across scales from single synapses to the entire brain. We provide resources quantifying synapse types and molecular distributions across the mouse brain (http://synaptome.genes2cognition.org) and a suite of analysis tools, including Synaptome Explorer for viewing individual synapses in the mouse brain. The generation and availability of these resources will be of widespread benefit in the neuroscience community.

## Results

### Synaptome Mapping Pipeline and Data Resources

**Figure 1:**
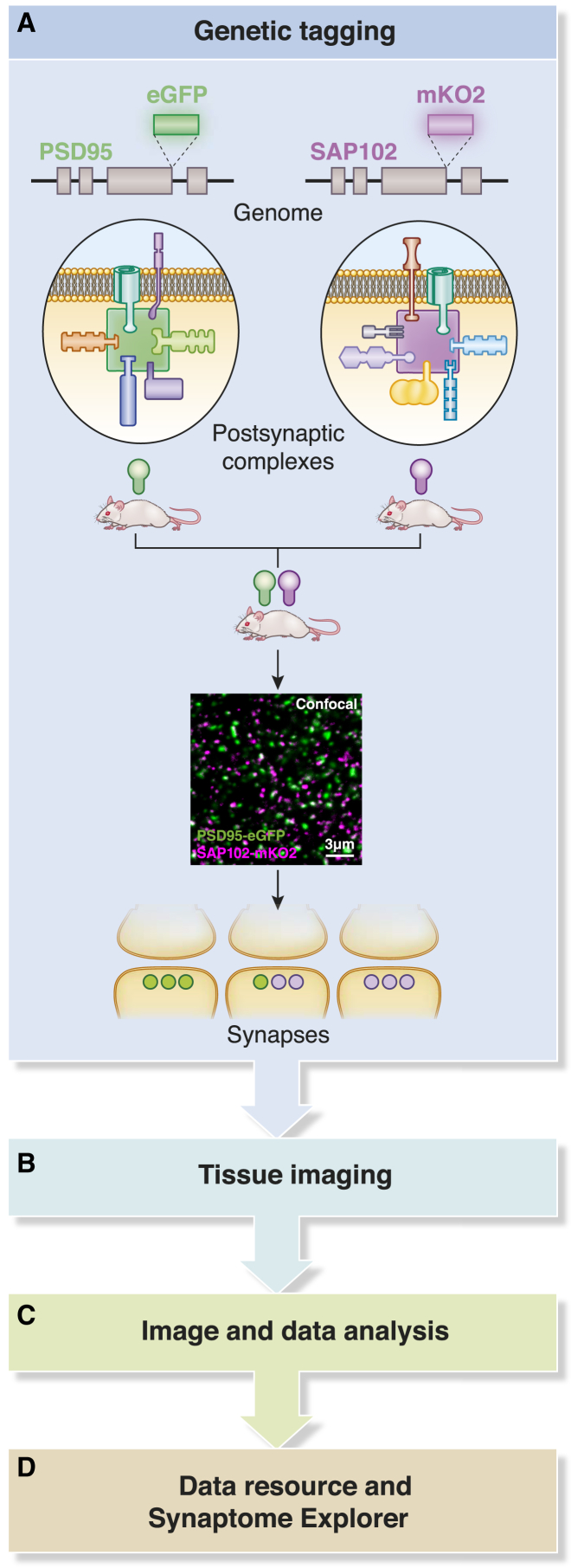
Synaptome Mapping Pipeline (A) Endogenous PSD95 and SAP102 were genetically tagged with eGFP (green) and mKO2 (magenta), respectively. These postsynaptic proteins assemble into signaling complexes at excitatory synapses. (B–D) Mice were crossed, and synaptic puncta (confocal image) expressing the fluorescent proteins were imaged in brain sections (B), analyzed (C), and stored and distributed (D).

**Figure 2:**
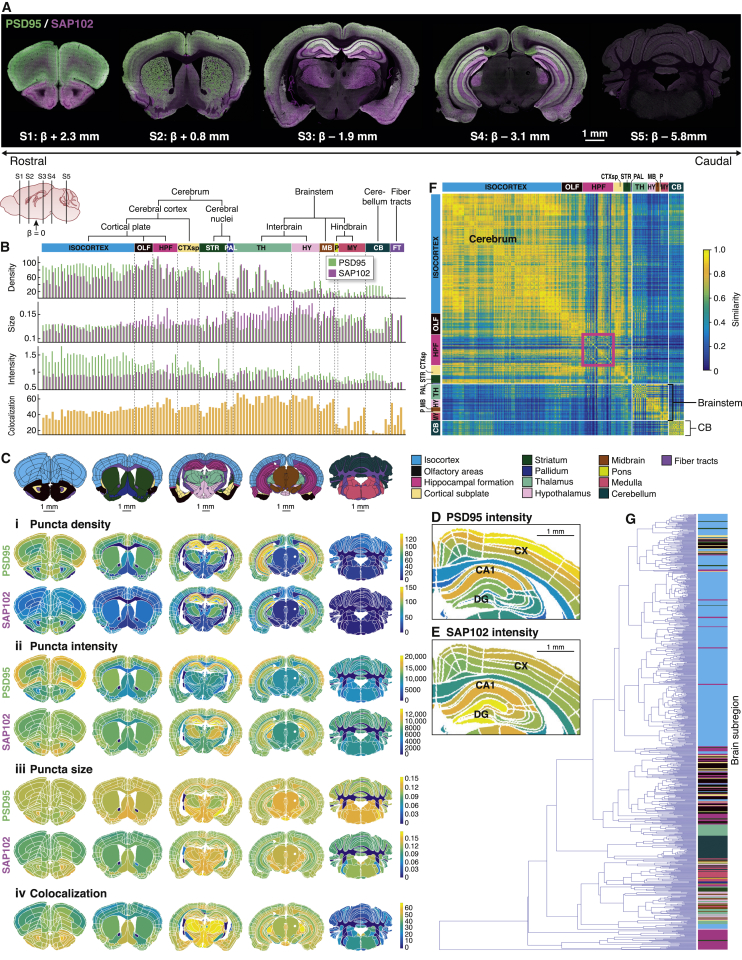
Whole-Brain-Scale Mapping of PSD95 and SAP102 (A) PSD95 (green) and SAP102 (magenta) expression in stitched down-sampled images of five coronal sections (S1–S5). Bregma (β) level is indicated. (B) PSD95 and SAP102 synaptome parameters in ARA anatomical subregions. Parameter units are as follows: density, number of puncta per 100 μm^2^; size, μm^2^; intensity, mean gray value per punctum (AU × 10^4^); colocalization, %. (C) Synaptome maps of delineated regions in five sections. Median punctum density (i), intensity (ii), size (iii), and colocalization (iv) for PSD95 (top) and SAP102 (bottom) are shown. Parameter units: density, number of puncta per 100 μm^2^; intensity, mean gray value per punctum (AU); size, μm^2^; colocalization, %. (D) PSD95 punctum intensity in delineated subregions of the hippocampus and cortex. CA1, cornu ammonis; CX, isocortex; DG, dentate gyrus. (E) SAP102 punctum intensity in delineated subregions of hippocampus and cortex. (F) Similarity matrix between pairs of subregions (rows and columns). White lines outline three major blocks (cerebrum, brainstem, and cerebellum). Pink box highlights hippocampal subregions. (G) Dendrogram showing hierarchical organization of subregions based on their similarity. Branch tips represent delineated subregions, colored as in (B).

To label and map the molecular composition of individual synapses across the whole mouse brain, we built a synaptome mapping pipeline (SYNMAP) consisting of four main components: (1) genetic tagging of synaptic proteins in knockin mice, (2) tissue imaging, (3) image and data analysis, and (4) data storage and dissemination (Figure 1). Because genetic tagging of endogenous proteins assures quantitative labeling, we fused PSD95 with eGFP and SAP102 with mKO2 using gene targeting, producing PSD95-eGFP and SAP102-mKO2 knockin mice (Figures 1A and S1). Normal protein expression levels (Figures S2A and S2B), regional expression (Figures S2C and S2D; Fukaya and Watanabe, 2000), postsynaptic localization (Figure S3), and absence of electrophysiological perturbations (Figures S4 and S5) confirmed that gene tagging did not detectably alter endogenous protein function. Both proteins labeled discrete postsynaptic puncta, and crossing of PSD95-eGFP and SAP102-mKO2 mice enabled comparisons of their expression patterns (Figures 1A, 2A, and S6).

Single-synapse-resolution imaging across brain sections required high-speed spinning disk confocal microscopy (SDM), which offers near-diffraction-limit resolution (∼290 nm in *xy*) (Huang et al., 2010). For each punctum, we measured a set of parameters, including intensity, size, and shape. The size and shape of synaptic junctions correlate with synaptic strength, efficacy, and plasticity (Ganeshina et al., 2004, Nusser et al., 1998, Takumi et al., 1999). Advanced computer vision and machine learning algorithms were developed to segment individual synaptic puncta, measure their spatial distribution, and classify them in an unsupervised manner. The validity of detection and quantification of synaptic punctum parameters by SDM was established by the high correlation of SDM data with published data (Broadhead et al., 2016) from laser scanning confocal microscopy (LSCM) and super-resolution gated stimulated emission depletion (g-STED) microscopy (Figure S7). Synapse diversity was evident in different neuronal types and brain regions; for example, hippocampal CA3 pyramidal neurons had large PSD95-eGFP puncta (characteristic of “thorny excrescence” synapses), in contrast to the small puncta in pyramidal neurons of the somatosensory cortex (Figure S3). Furthermore, different brain regions showed differential distributions of PSD95-eGFP and SAP102-mKO2 (Figure S6).

To study this synapse diversity at the whole-brain scale, we performed synaptome mapping by imaging five coronal sections covering 13 overarching brain areas: isocortex, olfactory areas (OLFs), hippocampal formation (HPF), cortical subplate (CTXsp), striatum (STR), pallidum (PAL), thalamus (TH), hypothalamus (HY), midbrain (MB), pons (P), medulla (MY), cerebellum (CB), and fiber tracts (FT) (Figure 2). These were further subdivided into ∼800 delineated subregions over both hemispheres, representing >300 unique subregions aligned with those in the Allen Reference Atlas (ARA) (Dong, 2008). We also developed an unsupervised mapping strategy devoid of *a priori* constraints on region boundaries in which the density of synapse parameters, type, or subtype (described below) were quantified in voxels (19 × 19 × 0.5 μm). The sets of synaptome maps are available on our website (http://synaptome.genes2cognition.org). We have also developed an interactive software application called Synaptome Explorer that allows visualization of individual synaptic puncta and their intensities, sizes, types, and subtypes across the five coronal sections (STAR Methods; Video S1).

Video S1. Synaptome Explorer, Related to Figure 2 An instructional video describing the use of the Synaptome Explorer: a visualization tool to view the molecular composition of billions of individual synapses at single-synapse resolution across the mouse brain. Alternatively the video is available on YouTube at https://www.youtube.com/watch?v=_ypYP_8fetE&feature=youtu.be.

### Hierarchical Patterning of Synapse Parameters

A striking and distinct anatomical patterning of PSD95-eGFP and SAP102-mKO2 puncta was observed in the low-magnification view of brain sections (Figures 2A and S6A). High-magnification images showed that this patterning reflected the differential distribution of the two proteins in populations of synapses in different brain areas (Figure S6B). Therefore, our first goal was to quantify the punctum parameters in synapse populations from delineated areas defined by the ARA. We observed unique regional distributions of punctum density (number of puncta per area), intensity (reflecting protein amount), and size (reflecting PSD size) for each protein, as well as for the percentage of puncta expressing both proteins (colocalization) (Figures 2B, 2C, and S8; Table S1). We also observed diversity between subregions as shown in the delineated cortical layers and hippocampal formation subregions (Figures 2D and 2E). These data reveal that each region and subregion has a characteristic “synaptome signature” of these parameters.

To explore the similarity of brain regions, we generated a similarity matrix from the synaptome signatures of ∼800 subregions (Figure 2F; Table S2). This revealed three major blocks of synaptome signature similarity: block 1 comprised all overarching areas from the cerebrum (except the pallidum), block 2 comprised all areas of the brainstem (and the pallidum), and block 3 defined the cerebellum. Strikingly, these three blocks broadly correspond to those regions arising from the first patterning of the nervous system, when the neural tube divides into three primary vesicles (forebrain, midbrain, and hindbrain) (Swanson, 2012). Moreover, each of these major blocks was composed of smaller blocks, many of which corresponded to hierarchical anatomical divisions within these regions. We examined the hierarchical organization of synaptome signatures with a dendrogram where subregions were clustered based on their level of similarity (Figure 2G). Subregions belonging to the same overarching areas typically clustered together. These results indicate that there is a hierarchical anatomical architecture to the distribution of synapse diversity in the global synaptome.

### Cataloging and Mapping Synapse Diversity

**Figure 3:**
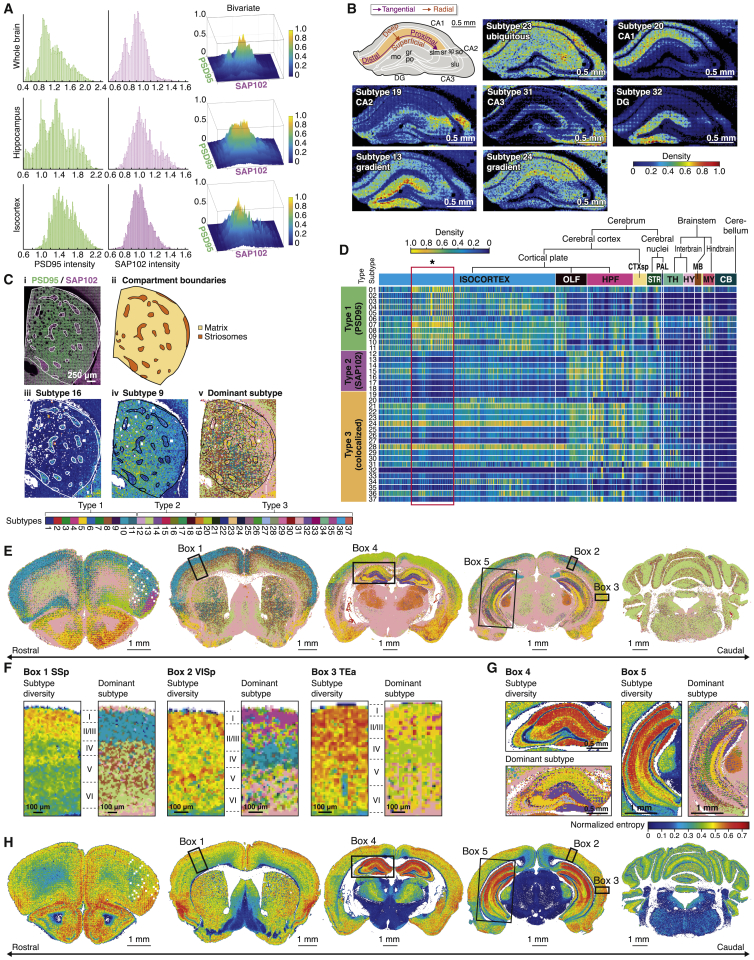
Catalog of Synapse Subtypes and Synaptome Maps (A) Probability density function of punctum intensities for PSD95 (left), SAP102 (middle), and PSD95 + SAP102 (right) in the whole brain (top), hippocampus (middle), and isocortex (bottom) reveals a multimodal distribution indicative of synapse subtype populations. Intensity, mean gray value per punctum (AU × 10^4^). (B) Synapse subtype distribution (density per 19.2 μm × 19.2 μm) in hippocampal formation. Top left: nomenclature of subregions and gradients. Ubiquitous subtype (23) and region-enriched subtypes (20, 19, 31, and 32). Reciprocal tangential gradients in CA1sr of subtypes 13 and 24 (see Figure S13 for all subtype maps). CA, cornu ammonis; DG, dentate gyrus; gr, granular layer; mo, molecular layer; po, polymorphic layer; slm, stratum lacunosum-moleculare; slu, stratum lucidum; so, stratum oriens; sp, stratum pyramidale; sr, stratum radiatum. (Ci) SAP102-rich/PSD95-poor patches (white delineated) within the caudate putamen nucleus (white outline). (Cii) Patches (seen in Ci) correspond to striosomes within the matrix. (Ciii and Civ) Examples of synapse subtypes with differential densities between striosome and matrix compartments; subtype 16 is highest in striosomes (Ciii), and subtype 9 is highest in the matrix (Civ). (Cv) Synaptome dominant subtype map showing differential expression between striosomes and matrix compartment and a mediolateral gradient. Key, synapse subtypes. (D) Density of the 37 synapse subtypes (rows) across 775 delineated regions (columns). Each subtype density was normalized (0–1) to its maximal density over all regions. Key, anatomical regions as in Figure 2B. (E) Synaptome dominant subtype maps showing subtype with highest density per area (19.2 μm × 19.2 μm). Subtype color key as in (C). Scale bars, 1 mm. (F) Dominant subtypes and diversity maps in cortical areas (boxes in E and H). Box 1, SSp (primary somatosensory area); box 2, VISp (primary visual area); box 3, TEa (temporal association area). Key, diversity in (H) and subtypes in (C). (G) Dominant subtypes and diversity maps in hippocampus. Box 4, rostral; box 5, caudal. Key, diversity in (H) and subtypes in (C). (H) Synaptome diversity maps showing the spatial distribution of normalized Shannon information entropy per area (19.2 μm × 19.2 μm). Boxes as in (F) and (G).

To better understand synapse diversity, it is necessary to classify or catalog synapses into different types. Presently, there are no catalogs of synapses generated from molecular and/or morphological measurements obtained across the whole brain. Using the molecular, size and shape parameters, we constructed a synapse catalog using an advanced machine learning technique developed in-house, called the weighted clustering ensemble method. As shown by the multiple peaks in the probability density distribution of the intensity in PSD95-expressing and/or SAP102-expressing synapses from the whole brain, cortex, or hippocampus, heterogeneous populations of synapses were differentially distributed in these brain regions (Figure 3A). We next classified all synapses into three major types according to the expression of the two proteins: type 1 express PSD95 only, type 2 express SAP102 only, and type 3 express both PSD95 and SAP102 (Figure S9). Next, using additional punctum parameters (STAR Methods; Figure S10A), we classified ∼1 billion individual synapses across all brain regions in a purely unsupervised manner and retrieved 37 synaptic subtypes: type 1 was divided into 11 subtypes, type 2 was divided into 7 subtypes, and type 3 was divided into 19 subtypes (Figures S9 and S10B).

To gain insight into the potential role of the comparatively large number of subtypes arising from the mapping of two postsynaptic proteins, we examined their anatomical distribution. For each subtype, we generated synaptome maps using both the delineated regions of the ARA (Figure S11) and the unsupervised voxel-based maps (Figure S12). These maps show discrete distributions for each subtype. For example, in the hippocampus, subtype 20 was enriched in CA1, subtype 19 was enriched in CA2, subtype 31 was enriched in CA3, and subtype 32 was enriched in DG, in contrast to subtype 23, which was ubiquitously expressed (Figures 3B and S13). Within the caudate putamen nucleus of the striatum, we observed patches enriched in subtype 16 (Figures 3Ciii and S14), whereas other subtypes, such as subtype 9 (Figures 3Civ and S14), were enriched outside of these patches. These SAP102-rich/PSD95-poor patches (Figure 3Ci) aligned with patches enriched in mu-opioid receptor (MOR) expression, a marker of striosomes (Koizumi et al., 2013), which are a sub-compartment of the striatum embedded within the matrix compartment (Figures 3Cii and S15A–S15C).

To contrast the differential distribution of the 37 synapse subtypes across the whole brain, we plotted the density of each in the ∼800 delineated regions of the ARA (Figure 3D). This revealed that each subtype has a unique distribution and that each region has a specific signature of synaptic subtype composition. Some subtypes were ubiquitously expressed across the brain (e.g., subtypes 6 and 7), whereas others showed more restricted regional expression. For instance, subtypes 24 and 28 were highly expressed in most cerebrum regions but nearly absent in the brainstem and cerebellum. Others showed preferential expression in the subregions of the cerebrum (e.g., subtype 32 in hippocampal formation), and multiple subtypes defined common subregions of the isocortex (Figure 3D, red box). We generated synaptome maps of the dominant subtype in each region (Figures 3E and S16A). These maps show striking patterning in cortical layers and hippocampal subregions (Figures 3F and 3G). Moreover, the cortical layers in the primary somatosensory (SSp), primary visual (VISp), and temporal association areas (TEa) differed from each other and revealed boundaries between and within cortical layers defined by classical cytoarchitectonic methods (Figure 3F, boxes 1–3).

The extent of synapse diversity in different brain regions is unknown. We therefore generated unsupervised synaptome maps of synapse diversity (Figures 3H and S16B). These showed that the highest diversity was in the hippocampal formation, followed by cortical regions, olfactory areas, and the striatum. In contrast to these areas, which are involved with higher cognitive functions, the brainstem, which controls basic behaviors, showed the lowest synapse diversity. The CA1 and DG subregions showed highest diversity within the hippocampal formation (Figure 3G), and the cortical layers in primary somatosensory, primary visual, and temporal association areas differed from each other and exhibited previously unknown layering patterns (Figure 3F).

These catalogs of synapse subtypes, based on the molecular organization of the postsynaptic proteome, revealed differential distribution of subtypes and complexity between brain regions and previously unknown anatomical boundaries and features. As a final step in this analysis, we examined the similarity matrix of subtype distribution between brain regions, which reveals organization within and between the classical topographic divisions of the brain (Figure S17). These findings suggest synapse cataloging and synaptome mapping are powerful techniques for deciphering the complexity of brain architecture.

### Synapse Diversity in Circuits and Connectome Networks

**Figure 4:**
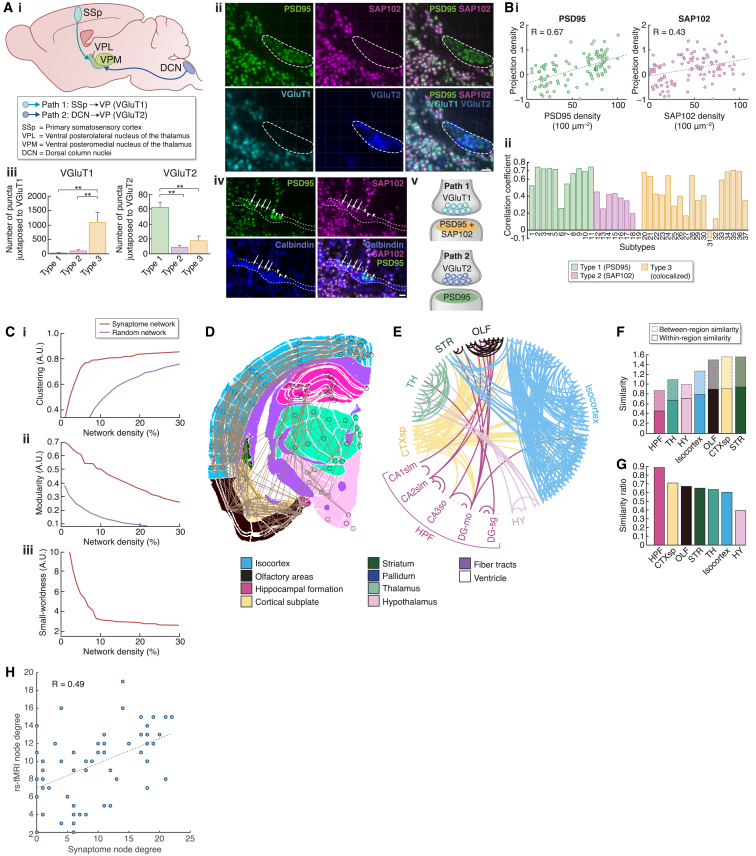
Synaptome and Connectome (Ai) Two main glutamatergic inputs to ventral posterior thalamus (VP) express VGluT1 and VGlut2 in their respective presynaptic terminals. Adapted from Graziano et al. (2008). (Aii) Co-labeling of PSD95 (green) and SAP102 (magenta) with presynaptic proteins VGluT1 (cyan) and VGluT2 (blue) in the VP. Scale bar, 1 μm. (Aiii) Quantification of PSD95-only (green), SAP102-only (magenta), or PSD95+SAP102 (orange) puncta juxtaposed to VGluT1 (left) or VGluT2 (right) (mean ± SD). ^∗∗^p < 0.01 post hoc Tukey. (Aiv) Co-labeling of PSD95 (green), SAP102 (magenta) and calbindin (blue) in VP. Arrows, colocalized synapses; arrowheads, PSD95-only synapses. Dashed lines delineate a calbindin-positive dendrite. Scale bar, 1 μm. (Av) Combinations of synaptic proteins define two connections: path 1, where VGluT1 contacts type 3 postsynaptic puncta (PSD95+SAP102); and path 2, where VGluT2 contacts type 1 postsynaptic puncta (PSD95 only). (Bi) Positive correlation (p < 0.05) between mesoscale maximal normalized projection density and PSD95 (left) and SAP102 (right) regional punctum densities. (Bii) Correlation coefficients between densities of 37 synapse subtypes and the mesoscale connectome projection density. (C) Synaptome network topology. Clustering coefficient (i), modularity (ii), and small-worldness (iii) of the synaptome network and random network. (D) Most significant connections (gray lines) between subregions (black circles) in section 3. (E) Circular graph of the 5% most significant connections of the binarized similarity between brain regions in section 3. (F) Between-region and within-region similarity of synaptome parameters for each overarching area from section 3. (G) The ratio of within-region to between-region similarities for synaptome parameters for areas in (F). (H) Correlation between synaptome and resting-state fMRI connectome node degree. Dots represent brain subregions. Correlation coefficient is shown (p < 0.0002).

The spatial distribution of synapse diversity raises the possibility that specific circuits are composed of particular types of synapses. To address this, we examined two long-range connections that converge on the ventral posterior nucleus of the thalamus (VP) (shown as pathways 1 and 2 in Figure 4Ai). Pathway 1, which descends from the somatosensory cortex, forms vesicular glutamate transporter 1 (VGluT1)-positive presynaptic boutons, whereas pathway 2, which arises from the brainstem, forms VGluT2-positive glomeruli (Graziano et al., 2008). Quadruple labeling of PSD95, SAP102, VGluT1, and VGluT2 was used to investigate the postsynaptic signatures of these two types of inputs (Figure 4Aii). We found that VGluT1 puncta were associated with type 3 puncta (i.e., expressing PSD95 and SAP102; p < 0.01), whereas VGluT2 puncta were juxtaposed to type 1 (PSD95-only) puncta (p < 0.01) (Figure 4Aiii). Furthermore, the type 1 and type 3 puncta were on the same dendrite of putative relay cells (identified by calbindin immunolabeling), revealing that two specific long-distance projections connect to molecularly distinct synapses on a single dendrite (Figures 4Aiv and 4Av).

To ask if these principles extend more broadly to long-range connections across the entire brain, we examined the synaptome signatures of brain regions connected by the mesoscale connectome atlas (Oh et al., 2014). We found a positive correlation between the projection density of connections and the density of PSD95 (R = 0.67, p < 0.0001) and SAP102 (R = 0.43, p < 0.0001) synaptic puncta (Figure 4Bi). We next asked whether there was a correlation between the subtype density of 104 subregions and their connections at the whole-brain scale. We found that type 1 (PSD95-only) subtypes (1–11, except 6) had a higher correlation (eight subtypes had R > 0.6) than type 2 (SAP102-only) types (subtypes 12–19), whereas type 3 (colocalized) types (subtypes 20–37) displayed both high and low correlations (Figure 4Bii). These results suggest that the synaptome architecture is a fundamental component of connectome organization.

The structure of connectome networks is known to be nonrandom and described by a topology with small-world architecture (Backus et al., 2016, Bullmore and Sporns, 2009, Mišić et al., 2014, Stafford et al., 2014, van den Heuvel and Sporns, 2011). We examined the global network topology of synaptome maps built from the similarity matrix, where each node represented a delineated brain subregion and edges that link nodes are scored for similarity of synaptome parameters (Figure 4C). Calculation of three topological properties (clustering coefficient, modularity, and small-worldness) showed the synaptome network had a higher clustering coefficient and modularity than random networks and high small-worldness (Figures 4Ci–4Ciii).

Hubs, which are nodes with high numbers of connections, are a central feature of small-world networks (Amaral et al., 2000, Barabasi and Albert, 1999), and fMRI studies found that the hippocampal formation was the major hub in functional connectomes (Backus et al., 2016, Mišić et al., 2014, van den Heuvel and Sporns, 2011). We asked which brain regions were hubs in the synaptome network by examining the connectedness (similarity) between regions and/or subregions. Strikingly, we found the hippocampus to be a hub using several approaches: (1) visualization of the synaptome network superimposed on the anatomical map (Figure 4D) and the circular graph (Figure 4E) showed that the subregions of the hippocampal formation (CA1slm, CA2slm, CA3so, DG-mo, and DG-sg) were frequently connected to other subregions, and (2) the hippocampal formation exhibited the highest ratios of within- to between-region similarity (Figures 4F and 4G), indicating it is a highly connected region and major hub in the global synaptome network. Thus, network analysis further reinforces the conclusion that synaptome architecture and the distribution of synapse diversity is a fundamental property of brain circuits and networks.

To examine the relationship between synaptome architecture and dynamic brain activity in the global network, we turned to the observation that whole-brain network activity measured by resting-state fMRI (rs-fMRI) shows small-world topology. Using mouse brain rs-fMRI data (Stafford et al., 2014), we compared the network node degree (i.e., the number of connections linking a region to other regions, the most fundamental parameter of network topology) (Bullmore and Sporns, 2009) between the synaptome and rs-fMRI networks. We found a significant correlation between rs-fMRI and synaptome node degree (R = 0.49, p < 0.0002) (Figure 4H). Collectively, these results suggest that the global architecture of synaptome networks is central to the structural and functional characteristics of connectomes.

### Gradients of Synapse Subtypes in the Hippocampal Formation

**Figure 5:**
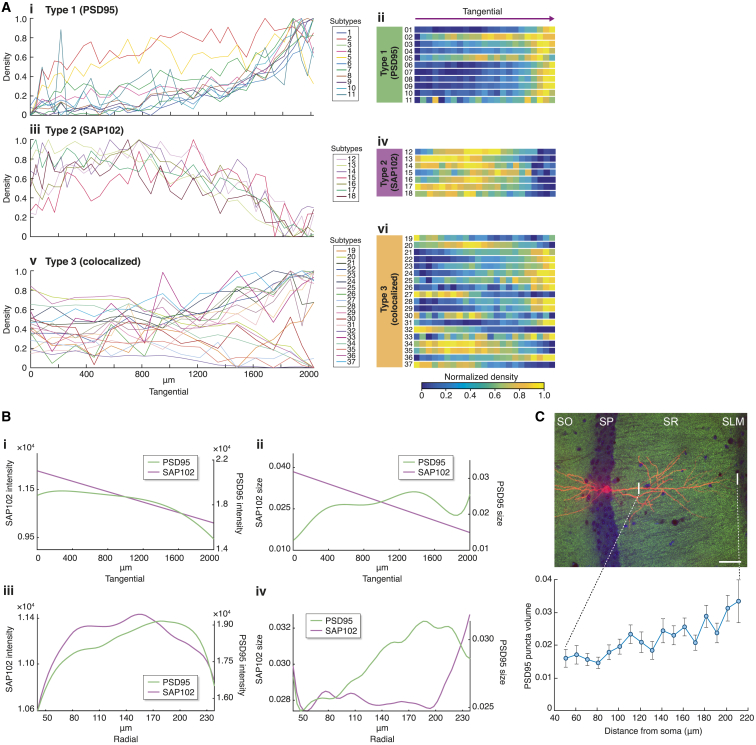
Synapse Gradients in the Hippocampal Formation (A) Line plots (i, iii, and v) and heatmaps (ii, iv, and vi) of the normalized density for type 1 (PSD95-only) (i and ii), type 2 (SAP102-only) (iii and iv) and type 3 (colocalized) (v and vi) subtypes on CA1sr tangential axis. Density unit, A.U. (B) Tangential and radial gradients of PSD95 and SAP102 punctum intensity (I and iii) and size (ii and iv) in CA1sr. Intensity, 16-bit grayscale; size, μm^3^. (C) Top: Alexa-594-injected pyramidal neuron (red) in the CA1 and PSD95 punctum labeling (green). DAPI staining, blue. Scale bar, 35 μm. SLM, stratum lacunosum-moleculare. SO, stratum oriens; SP, stratum pyramidale; SR, stratum radiatum; Bottom: PSD95 punctum volume (μm^3^) as a function of distance from the soma in apical dendrites of dye-filled CA1 pyramidal neurons (mean ± SEM). Dotted lines indicates range of plotted data.

The hippocampal formation plays a key role in cognitive functions, including the representation of spatial information in the pattern of nerve cell firing (O’Keefe and Dostrovsky, 1971). We were struck by the presence of synapse subtype gradients within the CA1 stratum radiatum (CA1sr), because an extensive literature describes gradients of synaptic electrophysiological properties (Andersen et al., 1980, Danielson et al., 2016, Igarashi et al., 2014, Pofantis et al., 2015). The density of subtype 13 gradually decreased along the tangential axis (toward CA2), whereas subtype 24 exhibited a gradient in the opposite direction (Figures 3B and 5A). When we systematically quantified the gradients for all subtypes, most of the type 1 (PSD95-only) subtypes showed an increasing distal-to-proximal tangential gradient (Figures 5Ai and 5Aii), type 2 (SAP102-only) subtypes showed a decreasing tangential gradient (Figures 5Aiii and 5Aiv), and type 3 (colocalized) subtypes showed a mixture of both gradients (Figures 5Av and 5Avi). We extended this analysis to the perpendicular radial gradient, thereby examining two dimensions (Figure 5B; Table S3). Both axes also revealed distinct gradients in PSD95 and SAP102 punctum parameters (size and intensity) (Figure 5B). Consistent with a previous super-resolution microscopy study (Broadhead et al., 2016), we confirmed that the distance-dependent increase in PSD95 punctum size along the radial axis (deep-to-superficial; Figure 3B) corresponded to the increase in PSD95 in spine heads of individual apical dendrites detected by dye-filled pyramidal cells (Figure 5C). Thus, the differential distribution of these proteins within (radially) and between (tangentially) pyramidal cell dendrites produces the gradients within the CA1sr.

We next asked if there was a correlation between these gradients and electrophysiological data recorded across the CA1 region (Chang and Jackson, 2006). Consistent with the major electrophysiological function of PSD95 (Béïque et al., 2006, Carlisle et al., 2008, Elias et al., 2006, Horner et al., 2018, Migaud et al., 1998), we found correlations between gradients of PSD95 and the peak postsynaptic response amplitude (tangential: R = 0.66, p = 0.0003; radial: R = 0.74, p = 0.05). This suggests that the spatial responses of the CA1sr are governed by the spatial distribution of synapse diversity of this region. Because PSD95 and SAP102 play a crucial role in modulating excitatory postsynaptic responses (EPSPs) to sequences of neuronal activity (Béïque et al., 2006, Carlisle et al., 2008, Elias et al., 2006, Horner et al., 2018, Migaud et al., 1998, Xu, 2011, Xu et al., 2008), we reasoned that the spatial variation in subtype composition could transform an incoming spatiotemporal pattern into a spatial map of activated synapses. This is an exciting possibility because it implies that synapse diversity could produce a functional output from a molecularly encoded representation.

### Functional Representations from Synaptome Maps

**Figure 6:**
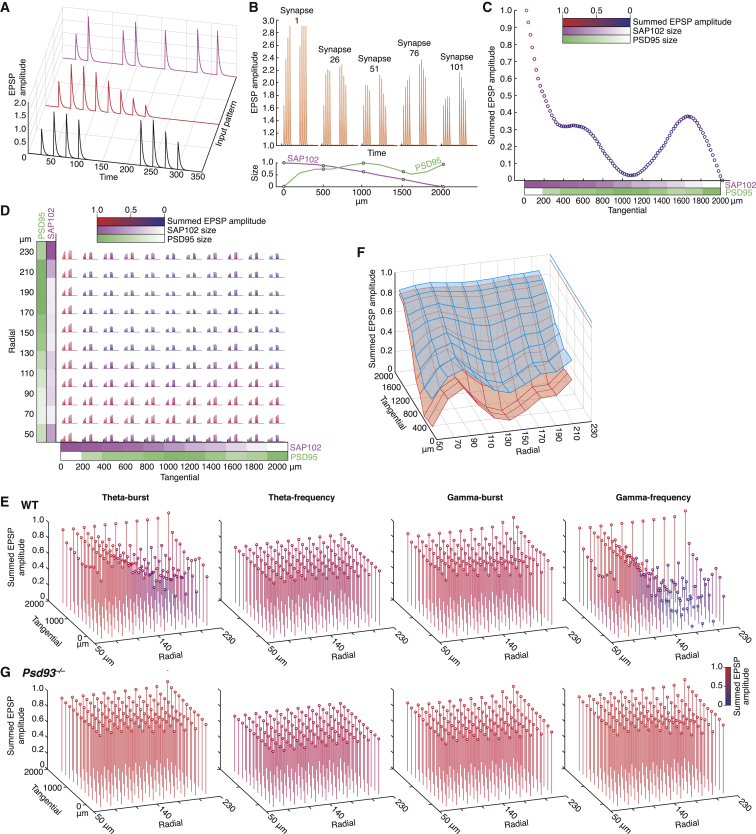
Computational Models of Synaptome Function (A) EPSP amplitude depends on the temporal stimulus pattern. Synaptic responses to theta-burst (black), gamma-frequency (red), and mixed-frequency stimuli (magenta). (B) Differential EPSP responses using hippocampal tangential gradient data. PSD95 and SAP102 size data (bottom) were used to model amplitude parameter values (top). Theta-burst induced EPSP responses over time are shown for five synapses (numbered 1–101) located along the CA1sr tangential axis. Circles in lower graph indicate size values used. (C) Normalized summed EPSP amplitude responses to theta-burst (as in B) shown for 101 synapses along the CA1sr tangential axis. Note regions of strong and weak responses. PSD95 and SAP102 gradients illustrated in the lower graph of (B) are shown here with graded colors (PSD95, green; SAP102, magenta). (D) EPSPs in response to two theta-bursts (as in A–C) in a two-dimensional synaptome map (11 × 11 synapses) derived from tangential (x axis) and radial (y axis) gradients in CA1sr. Normalized sum of EPSP peak amplitudes was color coded from blue (zero) to red (one). PSD95 and SAP102 gradients are shown with graded colors. (E) Normalized summed EPSP peak amplitude responses to different spike input patterns are mapped to different spatial locations and zones in a two-dimensional synaptome map of CA1sr (as in D). Four patterns, each comprising eight pulses, are displayed: theta-burst, theta-frequency, gamma-burst, and gamma-frequency. Amplitudes indicate normalized sum of EPSP amplitudes per synapse. (F) Normalized summed EPSP peak amplitude responses to spike patterns from correct (blue surface) and incorrect (red surface) choice trials are mapped to different spatial locations in a two-dimensional synaptome map of CA1sr (as in D and E). p < 0.05, two-sample Kolmogorov-Smirnov t test. Data are from Kim et al. (2016). (G) Normalized summed EPSP peak amplitude responses to different spike input patterns (as in E) are mapped to different spatial locations and zones in a two-dimensional synaptome map of CA1sr in *Psd93*^−/−^ mice. Note that the pattern specificity in WT (E) is largely lost, with only a difference in overall amplitude remaining.

To test this hypothesis, we constructed quantitative models of CA1sr synaptic physiology based on the molecular gradients. Using a computational biophysical model of synaptic transmission that exhibits short-term synaptic plasticity (Tsodyks and Markram, 1997, Varela et al., 1997), a “control” model was constructed to replicate experimentally derived EPSP amplitudes in response to paired-pulse and theta-burst stimulation. Figure 6A shows the responses of the control synapse to three different input patterns, illustrating how different temporal patterns could induce differential postsynaptic responses. To test if the molecular gradients of PSD95 and SAP102 along the tangential axis of CA1sr could result in spatially organized functional properties, we modified a set of 101 control synapses according to the punctum size profiles obtained from our synaptome maps (Figure 5Bii), changing their short-term plasticity characteristics. As shown by the individual EPSP responses to two theta-burst stimuli, five synapses interspersed along the tangential axis show differential responses (Figure 6B). The summed amplitude responses of each of the 101 synapses showed that alternating strong and weak synaptic responses emerge along the tangential axis (Figure 6C).

Next, we modeled a two-dimensional synaptome map based on the perpendicular tangential and radial gradients in hippocampal CA1sr (Figure 6D). This CA1sr synaptome map responded to an input of two theta-bursts by generating spatial zones with distinct EPSP profiles (Figure 6D). We next asked whether this functional mapping of patterns of activity to local zones of synapses varied according to the activity pattern by simulating four different patterns of activity, including theta and gamma frequency trains or bursts (with the same number of stimuli), which are patterns implicated in cognitive processes in the hippocampus and neocortex (Sirota et al., 2008) (Figure 6E). Each pattern of activity resulted in a unique functional synaptome map output: some zones showed a similar response to theta and gamma activity patterns, whereas other zones discriminated between the two stimuli. Bursts and trains also reveal a clear functional dichotomy: dramatic response zones were seen during theta-burst, but not theta-frequency, stimulation and in gamma-burst, but not gamma-frequency, stimulation (Figure 6E). These results are comparable to experimentally determined changes seen in short-term plasticity along the longitudinal axis of CA1, including gradual changes over the axis, diversified patterns of responses, response features common to all locations, and features specific to locations (Papaleonidopoulos et al., 2017). Together, these findings suggest that spatial organization of synapse diversity within the CA1sr can generate a multiplicity of functional representations from incoming patterns of neuronal activity.

### Behavioral Representations from Synaptome Maps

To investigate how synaptome map outputs would respond when animals are in different behavioral conditions, we used neuronal spike patterns recorded during a three-choice serial reaction time attention experiment (Kim et al., 2016) (Figure 6F). We compared the synaptome responses to patterns of activity recorded during trials ending in either a correct or incorrect response in model synapses as described above. In two-sample statistical tests (Kolmogorov-Smirnov test and Student’s t test, p < 0.05), the synaptome map outputs from the two responses were significantly different. Although the number of spikes was larger for incorrect trials, the synaptome map produced larger outputs for the correct trials (Figure 6F). Moreover, synapses in some zones displayed a larger differential response than in others. These results support the view that synaptome maps are a molecular neuroanatomical substrate for internal representations of behavioral responses involving decision-making.

### Synaptome Reprogramming

The aforementioned experiments provide support for a model where the spatial organization of synapse diversity arising from the expression of PSD95 and SAP102 could “store” or represent information in synaptome maps, which is then “recalled” with sequences of behaviorally relevant neural activity. This model suggests that in animals carrying mutations in synaptic proteins, changes in synapse diversity and synaptome maps will alter the stored representation and thus patterns of activity will produce a different spatiotemporal output.

To explore how mutations in synapse proteins affect synaptome maps, we reasoned that two main kinds of mechanisms merit consideration. The first is the direct impact of the mutation on the synapses that express the protein encoded by the gene. For example, a knockout (loss-of-function) mutation of SAP102 would have a direct effect on all SAP102-positive synapses (types 2 and 3, subtypes 12–37). Indeed, in the complete absence of SAP102, the proteome of these synapses would be directly altered. Thus, the catalog of synapses in the mutant mouse would be reorganized. In the second mechanism, a mutation in one postsynaptic protein causes an adaptive change in the expression of other synaptic proteins, which we refer to as “synaptome reprogramming.”

**Figure 7:**
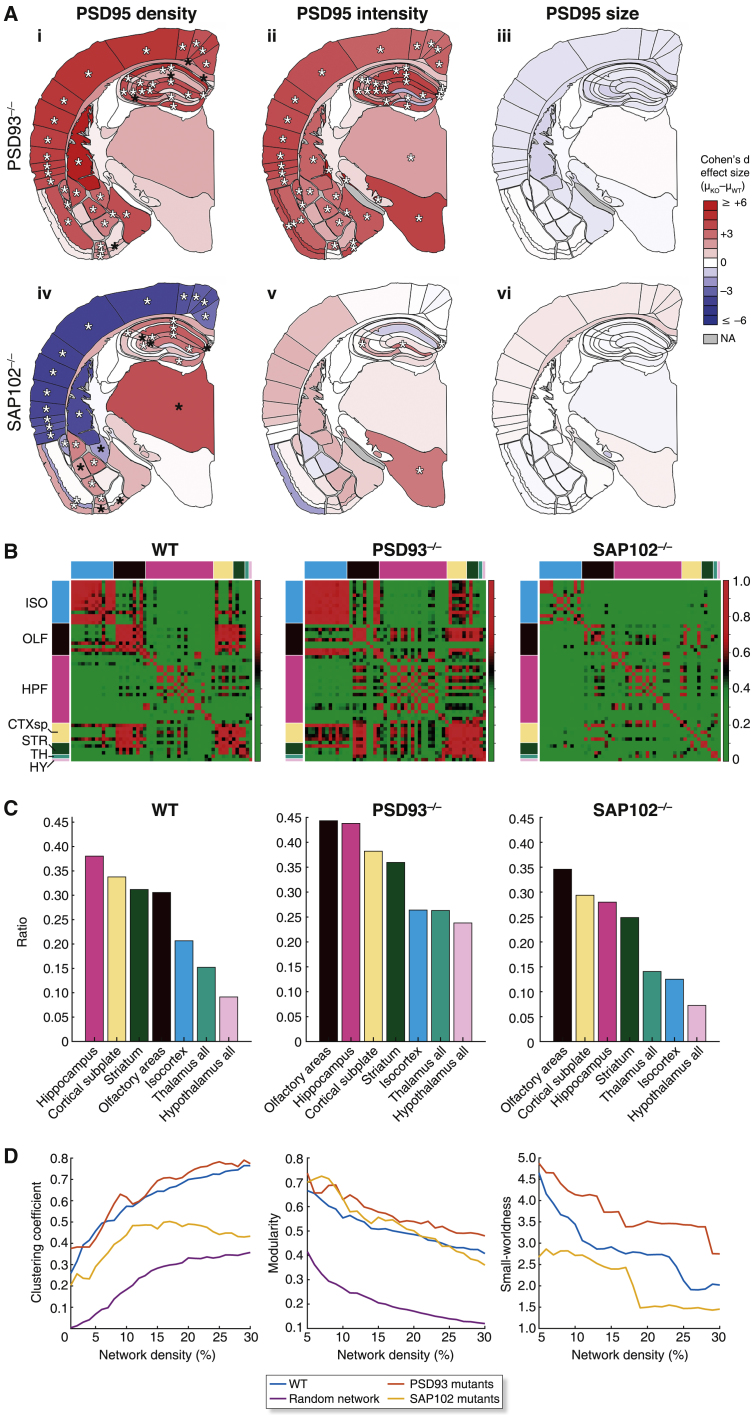
PSD93 and SAP102 Mutations Reprogram the PSD95 Synaptome (A) Cohen’s *d* values of changes in PSD95 punctum density (i and iv), intensity (ii and v), and size (iii and vi) in *Psd93*^−/−^ mice (i–iii, n = 6) and *Sap102*^−/−^ mice (iv–vi, n = 11) compared with wild-type (WT) mice (n = 13). Key, increase (red) or decrease (blue) relative to WT. Significance calculated using Bayesian estimation analysis with Benjamini-Hochberg correction: p < 0.05 (black asterisks), p < 0.01 (white asterisks). NA, not applicable. Raw values for PSD95 quantification in Table S4. (B) Subregion similarity matrices of PSD95 synaptome between pairs of subregions in WT, *Psd93*^−/−^ and *Sap102*^−/−^ mice. (C) Ratio of between-region to within-region similarities ordered from highest to lowest for regions in WT, *Psd93*^−/−^, and *Sap102*^−/−^ mice. (D) Clustering coefficient (left), modularity (middle), and small-worldness (right) for WT, *Psd93*^−/−^, and *Sap102*^−/−^ mice compared with a random-controlled network with an equivalent network complexity.

To test whether synaptome reprogramming occurs, we mapped the PSD95-eGFP synaptome in *Psd93* (also known as *Dlg2*) and *Sap102* (also known as *Dlg3*) knockout mice, which have abnormalities in synaptic physiology and behavior (Carlisle et al., 2008, Cuthbert et al., 2007, Nithianantharajah et al., 2013). We crossed the PSD95-eGFP mice with those carrying knockout mutations in *Psd93* and *Sap102* and compared the PSD95 synaptome maps in wild-type (WT; n = 13) and mutant backgrounds (*Psd93*^−/−^, n = 6; *Sap102*^−/−^, n = 11) in coronal sections (bregma level −1.8 mm) (Figures 7, S18, and S19). Widespread reorganization of the PSD95 synaptome map was observed in both mutant lines, indicating that synaptome reprogramming takes place in both mutants (Figures 7A, S18, and S19; Table S4).

Next, we asked if the synaptome reprogramming modified the global synaptome network topology. In both mutants, the regional similarity matrices showed reorganization; there was increased regional similarity in *Psd93*^−/−^ mice, in contrast to a marked reduction of similarity in *Sap102*^−/−^ mice (Figure 7B). Many areas showed major changes in the ratio of between-region to within-region similarities (as in Figure 4G) in both mutants (Figure 7C). To explore how the mutations reprogrammed the topology of the synaptome, we examined clustering coefficient, modularity, and small-worldness (Figure 7D). This again showed a striking dichotomy in which the PSD95 synaptome topology was reprogrammed in opposite directions by the two mutations: the network was less clustered and more randomized with reduced small-worldness in the *Sap102*^−/−^ mice, whereas in *Psd93*^−/−^ mice, it showed increased clustering, modularity, and small-worldness. No significant differences were detected in the cell density or size of delineated regions that could impact the synaptome changes observed (Figure S20). These data show that mutations in synaptic proteins induce large-scale synaptome reprogramming affecting the global topology of brain networks.

To test our hypothesis that reorganization of synaptome maps in animals carrying mutations would change the representation of information, we adapted our computational electrophysiological model of the two-dimensional CA1sr synaptome map using parameters based on electrophysiological data from *Psd93*^−/−^ mice (Carlisle et al., 2008). These mice show the same behavioral phenotypes as schizophrenia patients carrying *Psd93* mutations (Nithianantharajah et al., 2013). Comparison of the synaptome map outputs in WT (Figure 6E) and *Psd93*^−/−^ mice (Figure 6G) with the four patterns of activity described above shows that the spatial response to some patterns (theta-burst, gamma-frequency) was severely reduced in the mutants, whereas the response to other patterns was largely unaffected (theta-frequency, gamma-burst). In other patterns (data not shown), responses were increased, or there were novel responses (positive). These findings show that mutations in synaptic proteins reorganize synaptic diversity and alter the spatial architecture of synaptome maps at the global systems level and can change the capacity to represent information in the hippocampal formation.

## Discussion

### Synapse Diversity and Proteome Complexity

**Figure 8:**
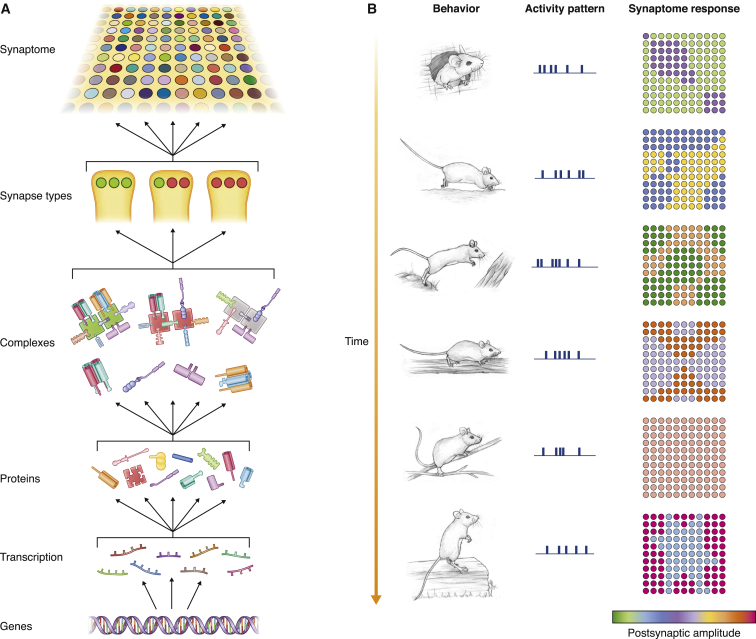
The Synaptomic Model (A) Synaptome architecture arises from a hierarchical molecular organization. Differentially expressed postsynaptic proteins are assembled into complexes (PSD95 complexes, green; SAP102 complexes, red). Complexes are inserted into postsynaptic terminals of excitatory synapses in different proportions producing synapse types and subtypes, which are spatially distributed (colored circles) into synaptome maps. (B) Synaptome map function in behavior and physiology. Each perception and action is associated with a pattern of neural activity. These patterns trigger postsynaptic responses of varying amplitudes in different synapse subtypes, producing zones and regions of differential activity in synaptome maps. As the mouse moves through its environment, the different patterns of activity produce a continuously varying synaptome output.

The synaptome of the mouse brain reveals that synapse diversity arising from combinations of postsynaptic proteins (Figure 8A) has the potential to generate an extraordinary number of excitatory synapse subtypes and synaptome maps; as few as ten proteins could produce 1,023 types and 10^11^ subtypes, which equals the total number of synapses in the mouse brain. Even though it is clear from our data that proteins are not randomly distributed into different synapses and the combinatorial diversity will therefore be constrained, the fact that the postsynaptic proteome contains >1,000 proteins and >200 multiprotein complexes (Frank et al., 2016) suggests synapse diversity will be vast. Moreover, we expect that posttranslational and other activity-dependent protein modifications (Coba et al., 2009, Collins et al., 2005, Trinidad et al., 2008) will generate further diversity, including transient and dynamic subtypes. The presynaptic and inhibitory synapse proteomes will also be expected to contribute to overall synaptome diversity.

Our findings suggest that synapse diversity increased in the vertebrate brain as a result of genome duplications early in the vertebrate lineage (Dehal and Boore, 2005, Holland et al., 2017). These duplications expanded the families of synapse proteins and multiprotein complexes (Grant, 2016, Nithianantharajah et al., 2013, Ryan and Grant, 2009, Ryan et al., 2008, Ryan et al., 2013) available for distribution into synapse types, allowing the evolution of neuroanatomical specializations. Thus, genome evolution and mechanisms controlling the assembly of proteins into multiprotein complexes provides a molecular logic to synaptome architecture in the vertebrate brain.

Each region of the brain was characterized by a signature of synapse subtypes, and each subtype showed a unique distribution pattern. The hippocampus and neocortex showed the highest overall diversity of excitatory synapses, and their synaptome maps revealed a plethora of previously unknown zones, boundaries, and gradients. The gradients of synapse subtypes in the CA1sr corresponded to known electrophysiological properties, and the brain-wide electrophysiological network, measured by resting-state fMRI, corresponded to the global synaptome architecture. Specific synapse subtypes were found in discrete circuits and the connected regions of the mesoscale structural connectome, revealing that circuits across the brain are defined by their synapse composition. In other words, the wiring diagram of the brain connects specialized local synaptome maps into a global brain network.

### The Synaptome in Information Storage and Recall

We studied how synapse diversity might contribute to cognition by focusing on the gradients of PSD95 and SAP102 found in the CA1sr. It was striking that the pattern of neural activity, such as a theta-burst train, generated differential EPSP responses in zones or regions and that other patterns of activity generated distinct and overlapping spatial outputs. This is an important finding, because it suggests that a multiplicity of representations can be “written” into a synaptome by the molecular composition of its synapses and that each representation can be accessed or “read” by a particular pattern of activity. The instantaneous response to changing patterns means that the synaptome map is continuously transforming temporally encoded information into changing spatial outputs. Thus, as an animal moves through its environment and receives changing sensory inputs that modulates the pattern of neuronal activity, the synaptome will generate a continuous “movie” of functional outputs (Figure 8B; Video S2). As such, synapse diversity and short-term plasticity provides a versatile mechanism for information storage and a simple and instantaneous recall mechanism.

Video S2. The Synaptome of the Mouse during Behavior, Related to Figure 8 A video describing how synaptome maps are created and how synapse diversity produces synaptome map responses during behavior. Alternatively, the video is available on YouTube at https://www.youtube.com/watch?v=HuU1UOGcenU.

This synaptomic model of cognition also raises interesting issues regarding the distributed nature and complexity of representations. Considering that some sensory stimuli are known to produce widespread activation of neurons across many regions (Musall et al., 2018), a global response from the synaptome would result. The synaptome architecture would produce distributed representations comprising a diversity of spatiotemporal responses. This would permit many different brain regions to contribute to the motor and other outputs. Interestingly, the hippocampus was found to be a hub in both the synaptome and functional connectome architectures (Mišić et al., 2014, van den Heuvel and Sporns, 2011), suggesting it participates in many representations and behaviors involving other regions of the brain.

### Synaptome Reprogramming

We asked if mutations could change the synaptome architecture and found a remarkable adaptive response, which we refer to as synaptome reprogramming. The PSD95 synaptome was changed in mice lacking PSD93, which is an integral component of PSD95 complexes (Frank et al., 2016). The PSD95 synaptome was also changed in mice lacking SAP102, which is a component of other complexes. Thus, the PSD95 synaptome architecture was reprogrammed by these two classes of mutations, which implies that potentially all mutations in postsynaptic proteins could cause synaptome reprogramming. This may be important for the more than 130 brain diseases that arise from postsynaptic mutations (Bayés et al., 2011). In addition to synaptome reprogramming, the loss of any synaptic protein would cause a direct effect on synapse diversity. For example, knockout of *Sap102*, which causes X-linked intellectual disability and autism (Lelieveld et al., 2016, Tarpey et al., 2004, Xing et al., 2016), will directly impact type 2 and 3 synapses. We also expect that mutations impacting transcription factors and other non-synaptic proteins that control expression of synaptic proteins will result in synaptome reprogramming. Thus, it is likely that changes in synapse diversity will be a core feature of many genetic brain diseases.

Synaptome reprogramming had widespread effects on brain regions and altered the synaptome network topology. There was also a striking and unexpected dichotomy in the synaptome reprogramming observed in the *Psd93* and *Sap102* mutants: they showed opposite effects on network topology and cortical puncta parameters, which parallels the dichotomy observed in their higher cognitive functions and electrophysiological properties (Carlisle et al., 2008, Cuthbert et al., 2007, Nithianantharajah et al., 2013). We also found that the *Psd93* mutation altered the conversion of activity patterns into a spatiotemporal output in the CA1sr. Thus, this mouse model of schizophrenia has altered representations, which may be directly relevant to the delusions and hallucinations experienced by these patients (Silverstein and Keane, 2011).

### Synaptome Resources and Applications

We have generated brain-wide atlases of synapse numbers and catalogs of synapse types and subtypes, and we derived many different maps, all of which are available on our website (http://synaptome.genes2cognition.org). We have also generated a novel Synaptome Explorer tool that can be used to interactively visualize brain sections at all levels of magnification allowing observations of individual puncta and their type and subtype classifications at the whole-brain scale. It takes ∼100 hr for imaging of two molecular markers in five coronal sections generating ∼10 Tb of data, ∼90 hr for image analysis (on a stand-alone workstation), and several days for manual delineation of brain regions and registration to the ARA. We anticipate a 20-fold improvement in throughput with ongoing methodological modifications. The SYNMAP pipeline is suitable for many molecular labeling methods, although we find that knockin mice carrying genetic labels are advantageous over antibody-based methods, because they reliably and reproducibly label every molecule and reduce the number of steps in the protocols.

We suggest it will be useful in future experiments to report the precise spatial coordinates of electrophysiological/optical recordings of synaptic function so that results can be assigned to the relevant synapse types/subtypes. Our study also provides a framework for generation of systematic unbiased synapse catalogs based on high-content image data and a standardized nomenclature describing synapse diversity based on molecular composition. The whole-brain synaptome maps reported here are first editions in a very large library encompassing all synaptic proteins. There will also be a need to map reprogrammed synaptomes in genetic and other disease models and modifications arising from development, aging, behavior, and experimental manipulations.

## STAR★Methods

### Key Resources Table

**Table:** 

REAGENT or RESOURCE	SOURCE	IDENTIFIER
**Antibodies**		

Mouse monoclonal anti-VGluT1(clone N28/9)	NeuroMab	Cat# 75-066; RRID: AB_2187693
Rabbit polyclonal anti-VGluT2	Synaptic Systems	Cat# 135 403; RRID: AB_887883
Rabbit polyclonal anti-MOR	Millipore	Cat# AB1580-I; RRID: AB_2716850
Mouse monoclonal anti-PSD-95 (clone K28/43)	NeuroMab	Cat# 75-028; RRID: AB_10698024
Mouse monoclonal anti-SAP102 (clone N19/2)	NeuroMab	Cat# 75-058; RRID: AB_10671660
Mouse monoclonal anti-PSD95 (clone K28/74) for western blotting	NeuroMab	Cat# 75-348; RRID: AB_2315909
IRDye 680RD Goat anti-Mouse IgG (H+L) for western blotting	LI-COR Biosciences	Cat#926-68070; RRID: AB_10956588
IRDye 800CW Goat anti-Rabbit IgG (H+L) for western blotting	LI-COR Biosciences	Cat#925-32211; RRID: AB_2651127
Rabbit polyclonal anti-Calbindin	Swant	Cat# CB 38; RRID: AB_10000340
Cy5-conjugated goat anti-mouse	Jackson ImmunoResearch	Cat# 115-175-205; RRID: AB_2338715
Cy5-conjugated donkey anti-rabbit	Jackson ImmunoResearch	Cat# 711-175-152; RRID: AB_2340607
Pacific Blue-conjugated goat anti-rabbit	Thermo Fisher Scientific	Cat# P-10994; RRID: AB_2539814

**Bacterial and Virus Strains**		

*E.coli* strain EL350 for recombineering	Lee et al., 2001	N/A

**Chemicals, Peptides, and Recombinant Proteins**		

DAPI	Sigma-Aldrich	Cat# D9542
DABCO (1,4-Diazabicyclo[2.2.2]octane)	Sigma-Aldrich	Cat# D27802
Alexa Fluor 594 dye solution	Life Technologies	Cat# A10442
ProLong antifade mounting medium	Life Technologies	Cat# P36930

**Deposited Data**		

Allen mouse brain reference atlas	Dong, 2008; ISBN: 9780470054086	http://mouse.brain-map.org/static/atlas
Mesoscale structural connectome	Oh et al., 2014; PMID: 24695228	http://connectivity.brain-map.org/
Resting-state fMRI connectome	Stafford et al., 2014; PMID: 25512496	http://www.pnas.org/content/111/52/18745.long?tab=ds
Optical recording of CA1 stratum radiatum	Chang and Jackson, 2006; PMID: 16873414	https://physoc.onlinelibrary.wiley.com/doi/10.1113/jphysiol.2006.112128
Supervised synaptome maps of PSD95 and SAP102	This paper	http://synaptome.genes2cognition.org/supervised_sm.html#
Supervised synaptome map of 37 synapse subtypes	This paper	http://synaptome.genes2cognition.org/supervised_subtypes.html#
Unsupervised synaptome map of 37 synapse subtypes	This paper	http://synaptome.genes2cognition.org/unsupervised_subtypes.html#
Synaptome Diversity map (unsupervised)	This paper	http://synaptome.genes2cognition.org/diversity.html#
Dominant subtype synaptome map (unsupervised)	This paper	http://synaptome.genes2cognition.org/dominant_subtype.html#
The PSD95 and SAP102 synaptome parameters in different brain subregions in the whole mouse brain scale	This paper	Table S1
The similarity matrix of the synaptome maps	This paper	Table S2
The PSD95 and SAP102 gradients in the CA1SR of the hippocampus	This paper	Table S3
PSD95 synaptome parameters in wild-type, PSD93^−/−^ and SAP102^−/−^ mice	This paper	Table S4
Mouse brain 3D model	Scalable Brain Atlas	https://scalablebrainatlas.incf.org/templates/ABA_v3/wholebrain.x3d
Synaptome data with individual synapse parameters, types, and subtypes across the 5 coronal mouse brain sections	This paper	https://doi.org/10.7488/ds/2366
An instructional video describing the use of the Synaptome Explorer: a visualization tool to view the molecular composition of billions of individual synapses at single-synapse resolution across the mouse brain	This paper	Video S1

**Experimental Models: Cell Lines**		

Mouse: E14Tg2a ES cells	Hooper et al., 1987; PMID: 3821905	N/A

**Experimental Models: Organisms/Strains**		

Mouse: *PSD95*^*eGFP/eGFP*^	This paper	N/A
Mouse: *SAP102*^*mKO2/mKO2*^	This paper	N/A
Mouse: *PSD93*^*−/−*^	McGee et al., 2001	N/A
Mouse: *SAP102*^*−/−*^	Cuthbert et al., 2007	N/A

**Oligonucleotides**		

Mouse genotyping primers PSD95-eGFP: exon F: CAAAGT GAAACGTGTCATCGAAG	This paper	N/A
Mouse genotyping primers PSD95-eGFP: 95UTR R: GAAGA AAGGCTAGGGTACGAAGG	This paper	N/A
Mouse genotyping primers PSD95-eGFP: eGFP F: AACCAC TACCTGAGCACCCAGTC	This paper	N/A
Mouse genotyping primers SAP102-mKO2: exon F: CATCAC AGGAGGGTCGTTACTAG	This paper	N/A
Mouse genotyping primers SAP102-mKO2: 102UTR R: GGG ACAAGAACAGTAGTCATTTG	This paper	N/A
Mouse genotyping primers SAP102-mKO2: mKO2 F: GCCA GATGAAGACCACCTACAAG	This paper	N/A

**Recombinant DNA**		

pneoflox-TAP vector	Fernández et al., 2009; PMID: 19455133	N/A
pTARGETER vector for gene targeting	Cuthbert et al., 2007; PMID: 1734405	N/A
peGFP-N1 vector carrying eGFP coding sequence	Clontech	NCBI: U55762
phmKO2-MN1 vector carrying mKO2 coding sequence	MBL	Cat# AM-V0146-NP

**Software and Algorithms**		

MATLAB	MathWorks	https://www.mathworks.com/
R for statistical computing	R projects	https://www.r-project.org/
Imaris	Bitplane	http://www.bitplane.com/imaris/imaris
ImageJ	NIH	https://imagej.nih.gov/ij/
SPSS	IBM Analytics	https://www.ibm.com/analytics/spss-statistics-software
Prism	GraphPad	https://www.graphpad.com/scientific-software/prism/
Illustrator	Adobe	https://www.adobe.com/products/illustrator.html
R code for comparison of WT/KO synaptomes using the Bayesian estimation method - Model	This paper	http://synaptome.genes2cognition.org/download_source.html
Triple Spots Colocalization – Imaris Plugin	Bitplane	http://open.bitplane.com/tabid/235/Default.aspx?id=91
Just Another Gibbs Sampler	SourceForge	https://sourceforge.net/projects/mcmc-jags/files/
Python 2.7.6	Python Software Foundation	https://www.python.org/download/releases/2.7.6/
Anaconda 1.9.2 Linux	Anaconda, Inc.	https://anaconda.org/anaconda
ImageMagick	ImageMagick Studio	https://www.imagemagick.org/script/index.php
OpenCV 2.4.6	Intel Corporation, Willow Garage, Itseez	https://opencv.org/
Synaptome Explorer v.1.0	This paper	https://github.com/SynaptomeMapping/SynaptomeExplorer/archive/master.zip
SDL2	Simple DirectMedia Layer	https://www.libsdl.org
Dear ImGui	GitHub	https://github.com/ocornut/imgui
GLEW	SourceForge	http://glew.sourceforge.net

### Contacts for Reagent and Resource Sharing

Further information and requests for resources and reagents should be directed to and will be fulfilled by the Lead Contact, Seth Grant (seth.grant@ed.ac.uk).

### Experimental Model and Subject Details

#### Gene Targeting and Mouse Generation

All mouse procedures were performed in accordance with UK Home Office regulations and approved by Edinburgh University Director of Biological Services. The gene targeting strategy for tagging PSD95 and SAP102 was previously described (Fernández et al., 2009) and was used to generate the PSD95-eGFP and SAP102-mKO2 targeting constructs. The template vector pneoflox-TAP contained mini homology arms of murine *Dlg4* (GeneID: ENSMUSG00000020886) or murine *Dlg3* gene (GeneID: ENSMUSG00000000881) that were PCR amplified from BAC clones (bMQ239c12 or bMQ312G21, respectively). Enhanced green fluorescent protein (eGFP, NCBI Accession number U55762) or mKO2 (MBL AM-V0146-NP) coding sequence, which follows a short linker sequence encoding for four amino acids (Gly-Gly-Gly-Ser), was inserted into the open reading frame of *Dlg4* or *Dlg3* at the 3′ end and immediately before its stop codon, respectively. The final targeting vector pTARGETER was constructed by recombineering techniques using a *E.coli* strain EL350 expressing λ phage gene products (recombinases), as a result, the final vector contained a 5′ homology arm and 3′ homology arm of *Dlg4* at the size of 6.3 kb and 2.9 kb, and for *Dlg3* at the size of 2 kb (5′ homology arm) and 5.7 kb (3′ homology arm) (Figure S1). All vector junctions and PCR cloned fragments were confirmed by Sanger sequencing. Correctly targeted ES clones (E14Tg2a) were identified by long-range PCR and microinjected into blastocysts for chimera generation. F1 generation pups presenting both WT and mutant bands on genotyping were considered as germline-transmitted heterozygotes. These heterozygous mice were crossed with a Cre-deleter mouse (CAG-Cre or CMV-Cre) to remove the loxP flanked *neo* cassette (Figure S1). To establish the reporter mouse lines, individual heterozygotes (PSD95^eGFP/+^ or SAP102^mKO2/+^) were further bred with C57BL/6J WT mice before interbreeding to create double-homozygous reporter mice, which were then maintained by intercrossing.

PSD95-eGFP and SAP102-mKO2 mice were genotyped by PCR using the following primer sets flanking targeted exon, FP coding region and endogenous 3′UTR. PSD95-eGFP mice genotyping primers: exon F: CAAAGTGAAACGTGTCATCGAAG; eGFP F: AACCACTACCTGAGCACCCAGTC; 95UTR R: GAAGAAAGGCTAGGGTACGAAGG; SAP102-mKO2 mice genotyping primers: exon F: CATCACAGGAGGGTCGTTACTAG; mKO2 F:

GCCAGATGAAGACCACCTACAAG; 102UTR R: GGGACAAGAACAGTAGTCATTTG

An adult (postnatal day 80 male) PSD95^eGFP/eGFP^;SAP102^mKO2/mKO2^ mouse was used for the mapping. The following groups of mice were used for the study of synaptome maps in mutant mice: WT (PSD95^eGFP/+^), n = 13 (9 m, 4f), age mean = 94.8, SD = 15.4; PSD93 mutant (PSD95^eGFP/+^;PSD93^−/−^), n = 6 (2 m, 3f), age mean = 90.3, SD = 3.4; SAP102 mutant (PSD95^eGFP/+^;SAP102^−/−^), n = 11 (3 m, 8f), age mean = 107.2, SD = 25.6. Five P70 male mice were used for cell-filling experiments.

### Method Details

#### Western Blotting

Forebrain tissue was homogenized in deoxycholate buffer (50 mM Tris pH 9.0, 1% sodium deoxycholate, 50 mM NaF, 20 μM ZnCl, 1 mM Na_3_VO_4_, 1 mM PMSF and 1 tablet/10 mL protease inhibitor cocktail tablets (Roche)) and clarified by centrifugation as previously described (Fernández et al., 2009, Husi et al., 2000). Each protein sample was quantified using a bicinchoninic acid assay (Pierce) and analyzed by SDS-PAGE. Ten mL of 5% milk PBS-T was added to the membrane, to block for 1–2 h at room temperature. Primary antibodies (PSD-95: 75-348, Mouse IgG1; Neuromab) (SAP102: 124213, Rb polyclonal; Synaptic System) used at a dilution of 1:1000 were diluted in 5 mL 1% milk PBS-T and incubated on rollers at 4°C overnight. The blot was washed for 15–20 min in PBS-T. Secondary antibody (IRDye secondary antibodies, LI-COR) was diluted in 5 mL 1% milk PBS-T and incubated on rollers at room temperature for 1 h. The blot was washed for 15–20 min in PBS-T, followed by rinsing once in PBS for 5 min before examined by Odyssey imaging system (LI-COR).

#### Electrophysiological Recordings

Electrophysiological recordings in the CA1 region of hippocampal slices were used to test for any functional consequences of eGFP and mKO2 tag insertions. Acute hippocampal slices were prepared as previously described (Kopanitsa et al., 2006). PSD95-eGFP animals were 2.5–4 months old and SAP102-mKO2 mice were 5.5–8.5 months old on the day of dissection. Field excitatory postsynaptic potentials (fEPSPs) were recorded using a MEA60 electrophysiological suite (Multi Channel Systems, Reutlingen, Germany). To record fEPSPs, a hippocampal slice was placed into the well of 5 × 13 3D MEA biochip (Qwane Biosciences, Lausanne, Switzerland). Monopolar stimulation of Schaffer collateral/commissural fibers through array electrodes was performed by STG4008 stimulus generator. Biphasic (positive/negative, 100 μs/a phase) voltage pulses were used. Amplitude, duration and frequency of stimulation were controlled by the MC_Stimulus II software. We performed all long-term potentiation (LTP) experiments using two-pathway stimulation of Schaffer collateral/commissural fibers (Andersen et al., 1977). A single principal recording electrode in the middle of proximal part of CA1 was chosen and two electrodes were assigned for stimulation of the control and test pathways on the subicular side and on the CA3 side of stratum radiatum respectively. The distance from the recording electrode to the test stimulation electrode was 420–510 μm and to the control stimulation electrode 316-447 μm. To evoke orthodromic fEPSPs, stimulation electrodes were activated at a frequency of 0.02 Hz. Peak amplitude of the negative part of fEPSPs was used as a measure of the synaptic response. Following at least 10–15 min of equilibration period inside an MEA well, I/O relationships were obtained and baseline stimulation strength was set to evoke a response that corresponded to ∼40% of the maximal attainable fEPSP at the principal recording electrode. Paired pulse facilitation (PPF) was observed after stimulating Schaffer collateral/commissural fibers with a pair of pulses at baseline stimulation strength and an interpulse interval of 50 ms. PPF value was calculated as fEPSP2/fEPSP1^∗^100%. Average data from five paired-pulse stimulations were used for each slice. LTP was induced after 60 min period of stable baseline responses by a theta-burst stimulation (TBS) train consisting of 10 bursts given at 5 Hz with 4 pulses given at 100 Hz per burst. Stimulus strength was not altered during LTP induction. LTP plots were scaled to the average of the first five baseline points. To account for a possible drift of baseline conditions, peak values in the test pathway were normalized by peak amplitudes in the control pathway prior to statistical comparison. LTP magnitude was assessed by averaging normalized fEPSPs in the test pathway 60–65 min after LTP induction. As several slices were routinely recorded from every mouse, values of area under the I/O relationship (AUC_I/O_), PPF and LTP in wild-type and mutant mice were compared using two-way nested ANOVA design with genotype (group) and mice (sub-group) as fixed and random factors correspondingly (STATISTICA v. 10, StatSoft, Tulsa, OK, USA). Statistical effects were considered significant if p < 0.05. Graph plots and normalization were performed using OriginPro 8.5 (OriginLab, Northampton, MA, USA). Data are presented as the mean ± standard error of the mean with *n* and *N* indicating number of slices and mice respectively.

#### Tissue Collection and Sectioning

Adult mice were anesthetized by an intraperitoneal injection of 0.1 mL of 20% w/v sodium pentobarbital (Euthatal, Merial Animal Health or Pentoject, Animalcare). After complete anesthesia, 10 mL of phosphate buffered saline (PBS; Oxoid), was perfused transcardially, followed by 10 mL of 4% v/v paraformaldehyde (PFA; Alfa Aesar). Whole brains were dissected out and post-fixed for 3–4 h at 4°C in 4% PFA then cryoprotected for 3 days at 4°C in 30% sucrose solution (w/v in 1 × PBS; VWR Chemicals). Brains were then embedded into optimal cutting temperature (OCT) medium within a cryomold and frozen by placing the mold in isopentane cooled-down with liquid nitrogen. Brains were then sectioned in the coronal plane at 18-μm thickness using a NX70 Thermo Fisher cryostat, and cryosections were mounted on Superfrost Plus glass slides (Thermo scientific) and stored at −80°C.

#### Histology and Immunohistochemistry

Sections were washed for 5 min in PBS, incubated for 15 min in 1 μg/mL DAPI (Sigma), washed and mounted using home-made MOWIOL (Calbiochem) containing 2.5% anti-fading agent DABCO (Sigma-Aldrich), covered with a coverslip (thickness #1.5, VWR international) and imaged the following day. For immunohistochemistry, sections were first washed for 5 min in PBS. For SAP102 immunostaining experiments only, an antigen retrieval step was then carried out by incubating the section in citric acid (2.1 g/L in distilled water, pH 6.0) and placing it in a pressure cooker at 120°C. for 30 s. For all immunostainings, sections were then incubated for 1 h in Tris-buffered saline (TBS) with 5% bovine serum albumin (BSA, Sigma-Alrich) and 0.5% Triton X-100 (Sigma-Alrich) and then incubated overnight at 4°C with primary antibodies diluted in a solution of TBS, 3% BSA and 0.5% Triton X-100 (Calbindin D-28k, 1:500, Swant CB38; MOR, 1:500, Millipore AB1580-I; PSD95, 1:250, Neuromab 75-028; SAP102, 1:250, Neuromab 75-058; VGluT1, 1:250, NeuroMab 75-066; VGluT2, 1:250, Synaptic Systems 135403). Sections were then washed three times for 10 min in TBS with 0.5% Triton X-100, incubated for 2 h with secondary antibodies diluted in a solution of TBS, 3% BSA and 0.5% Triton X-100 (Cy5 anti-mouse IgG1, 1:500, Jackson laboratories 115-175-205; Cy5 anti-rabbit IgG, Jackson laboratories 711-175-152; Pacific blue anti-rabbit IgG, 1:500, Millipore P-10994) and washed three times for 10 min in TBS with 0.5% Triton X-100 before mounting of the coverslip as described above.

#### Neuronal Cell Filling

Adult mouse brains (n = 5) were perfused and removed as described above and post-fixed in 4% PFA overnight at 4°C. After washing in PBS, coronal sections (200 μm thick) were then cut with a Vibratome and prelabeled with 10^−5^ M 4,6 diamidino-2-phenylindole (DAPI: Sigma D9542 St. Louis, MO, USA) to identify cell nuclei. Pyramidal cells in the CA1 region of the hippocampus were then individually injected with Alexa Fluor 594 dye solution (Life Technologies), by continuous current until the distal tips of each cell fluoresced brightly, indicating that the dendrites were filled completely. After injections, sections were mounted in ProLong antifade mounting medium (Life Technologies). Sections were then analyzed with a Zeiss LSM 710 Confocal microscope. Fluorescently labeled profiles were examined through separate channels, using excitation peaks of 585 and 491 nm to visualize Alexa Fluor 594 and eGFP, respectively. Consecutive stacks of images (ranging from 3 to 5) were acquired at high magnification (63 × oil immersion; 0.14 z-step) to capture the length, depth, and width of main apical dendrites (n = 7). For each stack of images, confocal parameters were set so that the fluorescence signal was as bright as possible while ensuring that there were no pixels saturated within the PSDs and spines. PSDs and spine volumes were analyzed using Imaris 7.6.5 (Bitplane AG, Zurich, Switzerland). Over 1000 manually reconstructed PSDs and corresponding spines were reconstructed along the length of apical dendrites (see Benavides-Piccione et al., 2013) for detailed information regarding 3D reconstruction). Measurements are reported as the mean ± SEM.

#### Spinning Disk Confocal Microscopy

For synaptome mapping, two types of Spinning Disk confocal Microscopy (SDM) platforms were used. The initial mapping of five coronal sections was performed using the Cell Voyager 1000 (CV1000, Imsol) equipped with a 100X lens (NA 1.4), a CSU-W1 spinning disk (Yokogawa) with a pinhole diameter of 50 μm and a Hamamatsu back-illuminated EMCCD camera. A Z stack containing five optical slices was acquired with an interval of 0.1 μm for a final voxel dimension of 76 × 76 × 100 nm and a depth of 16 bits. To cover entire brain sections, several overlapping mosaic grids with a constant optical range were set-up. For the comparison of PSD95 synaptome between WT and mutant mice, the Andor Revolution XDi was used with an Olympus UPlanSAPO 100X oil immersion lens (NA 1.4), a CSU-X1 spinning-disk (Yokogawa) and an Andor iXon Ultra monochrome back-illuminated EMCCD camera. Images acquired with that system have a pixel dimension of 84 × 84 nm and a depth of 16 bits. A single mosaic grid was used to cover each entire brain section with an adaptive Z focus set-up by the user to follow the unevenness of the tissue using the Andor iQ2 software. In both systems, eGFP was excited using a 488 nm laser and mKO2 with a 561 nm laser. The CV1000 system is equipped with the following filters: BP 525/50 nm for eGFP and BP 617/73 nm for mKO2 whereas the Andor Revolution XDi is equipped with a Quad filter (BP 440/40, BP 521/21, BP 607/34 and BP 700/45). For both systems, mosaic imaging was set-up with no overlap between adjacent tiles.

#### Computational Modeling

We used a computational biophysical model of synaptic transmission including synaptic short-term plasticity (STP) to study the relation between incoming temporal spike patterns and the resulting synaptic activation and compared activations over regions with differences in synaptic properties. Simulations were performed using MATLAB, R2015b with a time discretization of 1 ms.

##### Synaptic Responses

Synaptic EPSPs evoked by an incoming event (transmitter release following a presynaptic spike) were described by a bi-exponential function. Parameters τ1 and τ2 were set to reproduce a fast ionotropic synaptic AMPA-type time course.

$$\text{V}={\text{A}}_{\text{e}}\times \left(\exp\left(-t/\mathrm{\tau 1}\right)-\exp\left(-t/\tau 2\right)\right)$$

where ${\text{A}}_{\text{e}}=\Pi_{\text{i}}1\times {\text{A}}_{\text{tfi}}\times {\text{A}}_{\text{tdi}}$, τ1 = 3.0 ms, τ2 = 0.4 ms, i index of all preceding spikes

Short-term synaptic changes followed a formalism described by Tsodyks and Markram (1997) and Varela et al. (1997). We included one fast and one slow facilitating component and one depressing component, all which affected synaptic responses following the triggering one. In all figures, amplitudes were shown normalized to the amplitude of the first response.

##### Depression

$${\text{A}}_{\text{di}}={\text{A}}_{\text{d}}\times \exp\left(-\Delta t/\tau_{\text{d}}\right)$$

where

Δt_i_ is the time between the preceding event i and the present event
A_d_ = A_d0_ × S_Ad_, S_Ad_ is normalized tangential PSD95 size factor^∗^, [0, 1]
τ_d_ = τ_d0_ × S_Td_, S_Td_ is normalized radial PSD95 size factor^∗^, [0, 1]
A_tdi_ = max (Σ_i_(1-A_di_), 0), total depressing response was limited to positive values.

##### Fast facilitation, F1

$${\text{A}}_{\text{fi}}={\text{A}}_{\text{f}}\times \exp\left(-\Delta t_{i}/\tau_{\text{f}}\right)$$

where

A_f_ = A_f0_ × S_Af_, S_Af_ is normalized tangential SAP102 size factor^∗^, [0, 1]
τ_f_ = τ_f0_ × S_Tf_, S_Tf_ is normalized radial SAP102 size factor^∗^, [0, 1]

##### Slow facilitation, F2

$${\text{A}}_{\text{si}}={\text{A}}_{\text{s}}\times \exp\left(-\Delta t_{i}/\tau_{\text{s}}\right)$$

where

$${\text{A}}_{\text{s}}={\text{A}}_{\text{s}0}$$

$$\tau_{\text{s}}=\tau_{\text{s}0}$$

The total facilitatory response had a saturation at 3.3 times the unit response (Zucker and Regehr, 2002).

$${\text{A}}_{\text{tfi}}=\min\left(1+\sum_{\text{i}}\left({\text{A}}_{\text{fi}}+{\text{A}}_{\text{si}}\right),3.3\right)$$

^∗^For Figures 6B–6G, differential model parameter values along the spatial dimension were obtained from the experimental tangential and radial profile data of PSD95 and SAP102 normalized size data (Figure 5B). Values for the spatial locations used in the model were interpolated from data using cubic B-splines.

Time constants τ in unit ms and amplitudes in a.u (arbitrary units). Control parameters were set to replicate experimental data of synaptic amplitudes in response to a 10 cycle theta-burst protocol. STP-parameters were estimated using the Nelder-Mead Simplex method. For estimation of control model, all six parameters were free. The model was fitted to amplitude data from theta-burst experiments for bursts 1, 2, 8 and 10 in a 10 burst protocol containing four pulses per burst. Verification tests showed that including a second, potentially slower, depression factor did not significantly reduce the error (data not shown). PSD95 and PSD93 parameters were set to replicate the respective paired-pulse facilitation fractional differences between recordings in tissues from WT and knockout animals (IPI = 25, 50, 100, 200 ms) (Carlisle et al., 2008). For the estimation of the parameters in knockout models, only A_d0_, τ_d0_ and A_f0_ were allowed to change. Verification and parameter sensitivity tests showed that inclusion of three other parameters did not significantly affect the fitting error (data not shown).

##### Stimulation Patterns

Theta-burst, four pulses per burst, interburst interval (IBI) = 125 ms, interpulse interval (IPI) = 25 ms

Theta, IPI = 200 ms
Gamma, IPI = 25 ms
Gamma doublets, two pulses per doublet, IBI = 300 ms, IPI = 13 ms
Mixed pattern in Figure 6C, stimulation time (ms): 25, 50, 120, 145, 205, 270, 310

##### Normalized Synaptic Response

To quantify synaptic map responses, the synaptic response was defined as the sum of peak EPSP amplitudes produced by a stimulating pattern. Figures 6C and 6E–6G shows the normalized sum for the values obtained for that figure, except for panels F and H, which share the normalization.

##### Prefrontal Cortex Data

Data from behavioral experiments using chronic electrophysiological recordings during a 3-choice serial reaction time attention experiment (Kim et al., 2016) were used. Neurons were identified as putative parvalbumin-positive inhibitory interneurons based on local-field potential features (including narrow spike characteristics) as well as firing frequency (f > 10 Hz). To verify the identification, these neuron data were compared to that obtained from optogenetically tagged and optically stimulated neurons. We compared synaptome map responses to spike patterns recorded during trials ending in a correct response (the correct port visited) to those ending in an incorrect response (one of the wrong ports visited). Data from 10 neurons recorded during trials ending in a correct response (705 spikes in total) and 10 neurons from incorrect trials (917 spikes in total) were used. Data were taken from the waiting time period, between trial onset and stimulus cue presentation, a time period defined as the sustained attention period. Model synaptic map responses for each synapse were taken as the sum of amplitudes as described above. Responses from the two conditions were further analyzed. For the correct responses, the ratio of largest over smallest synaptic map response was 1.7 and for incorrect responses it was 2.4. Responses from the two conditions were further compared and found different in two-sample Kolmogorov-Smirnov goodness-of-fit hypothesis test as well as t test, both at 5% level of significance.

### Quantification and Statistical Analysis

#### Detection of Synaptic Puncta

Punctum detection was carried out using image detection algorithms, Ensemble Detection developed in house. Detection of the synaptic puncta is a key and fundamental step in the SYNMAP pipeline. Our synaptic punctum images were usually acquired in a low signal-to-noise-ratio (SNR) environment and puncta were diverse in the intensity distribution, as shown in the PSD95 and SAP102 intensity PDF (Figure 3A). Existing punctum/particle detection algorithms (Chenouard et al., 2014) in fluorescence microscopy can only process images with similar punctum intensities and background noise, or require significant fine tuning of algorithm parameters. Therefore, they can only be applied to fluorescence microscopic images collected within a small area of tissue and not perform robustly detect synaptic puncta in our data at the whole brain scale.

To address the problem, we have developed a new punctum/particle detection method based on the multi-resolution image feature detector and supervised machine learning technique. In specific, we proposed a multi-resolution and multi-orientation version of 2^nd^-order nonlocal derivative (NLD) developed in our previous work (Qiu et al., 2012) and used it to calculate intensity differences, referred to as image features, for each of all individual puncta at different spatial resolutions and orientations. For PSD95, 33 image features were calculated per punctum and for SAP102, 105 features were used per punctum. These intensity differences were then assembled as feature vectors of each individual puncta for classification and detection of puncta. An initial intensity threshold is set to a very low value only to filter out extremely dim puncta and to avoid missing true synaptic puncta. The remaining candidate puncta were finally classified as either true puncta or background noise using the corresponding feature vectors and the classifier. The classifier was pre-trained with the training image set and machine learning algorithms (Zhou, 2012). The training set was randomly selected from the whole-brain synapse images of over ∼800 delineated subregions. The puncta in these images were then manually annotated independently by two different experts. Ensemble learning (Zhou, 2012) was selected as the classifier learning algorithm as it has proven performance in predicting generalization error (Breiman, 2001) in machine learning and hence, is suitable to classify puncta with diverse intensities in our dataset.

#### Measurement of Synaptic Parameters

After being detected and localized, all puncta were then segmented adaptively based on their individual intensity values: for each punctum a threshold was set as 10% of the maximum pixel intensity within the punctum, so that punctum size and shape measurement were independent of punctum intensity. This is in analogy with the super-resolution microscopy, where the optimal resolution was measured as the full-width-at-half maximum of the puncta (Betzig et al., 2006). With puncta segmented and binarized, six punctum parameters were then calculated including mean punctum pixel intensity, punctum size, skewness, kurtosis, circularity, and aspect ratio. The latter four parameters were used to quantify the punctum shape: skewness was for measuring asymmetry of puncta intensity profiles, kurtosis was for measuring the sharpness of intensity profiles, circularity, and aspect ratio was for measuring the roundness of the puncta (Schneider et al., 2012).

#### Colocalization of Synaptic Puncta

The colocalization analysis determines whether one PSD95 punctum and one SAP102 punctum were co-expressed in the same post-synaptic density (PSD). It was measured based on the spatial distance between two puncta: puncta with the centric distance smaller than a given distance threshold were considered as being colocalized. The threshold in our analysis was set as 500 nm based on the typical PSD diameter (Sheng and Kim, 2011).

#### Classification of Synaptic Puncta

Unsupervised classification was applied to ∼1 billion individual synapses to group synaptic puncta of similar parameters into 37 subtypes. A new classification method, weighted ensemble clustering (WEC) algorithm, was developed in order to automatically generate a robust and validated classification results by combining clustering results from different clustering methods, each of which often performs well only for a specific type of statistical PDF (Wiwie et al., 2015). Eight state-of-the-art clustering methods (Wiwie et al., 2015) were independently applied to ∼1 billion of puncta and each method was used by varying number of clusters from 1 to 300 for initial clustering. This gave rise to a comprehensive pool of clustering that contained 2400 different clustering results on the same dataset. Each clustering result was then quality evaluated based on the 11 clustering validity indices (Liu et al., 2013) in machine learning. These indices were finally used as weights to combine 2400 clustering results: results with higher validity indexes were given more priority in the final clustering result, and the cluster number was selected from results of the highest validity indices. By using WEC, we could finally generate a high-quality clustering result with algorithmically validated cluster number.

#### Mapping of Synaptome Parameters

After detection and segmentation, all punctum parameters were first averaged per volumetric area (19.2 μm × 19.2 μm × 0.5 μm), so that each area was represented by one set of parameters instead of parameters of all puncta within it. For supervised mapping in Figures 2C, 2D, 3D, and 7A, the parameters were then averaged over all volumetric areas of all delineated subregions shown in Figure 2C. Subregion delineation was performed on stitched/downsized images using polygon selection tool of Fiji software based on the the Allen Reference Atlas of coronal adult mouse brain.

#### Diversity and Subtype Maps

The Synaptome Diversity map in Figure 3H was built based on normalized Shannon entropy (Mackay, 2003), where the 37 subtypes were histogram bins for discrete random variable *X*, and the population per bin was the density per subtype. Shannon entropy was normalized between 0 and 1 by dividing by log_2_37.

The Dominant Subtype map in Figure 3E was built based on the largest densities of subtypes per volumetric area: subtype index with the largest population per volumetric area was used as the pixel intensity in the map.

#### Validation of SYNMAP

The correlation between the density, intensity, and size parameters measured for PSD95 in the hippocampus from Section 3 were correlated with previously published LSCM and g-STED microscopy data (Broadhead et al., 2016). Six hippocampal subregions common to the two studies were used to calculate the Pearson correlation coefficient and corresponding p values using SPSS software.

#### PSD95/SAP102 Juxtaposition with VGlut1/VGluT2

For imaging of PSD95/SAP102/VGluT1/VGluT2 quadruple labeling in the VP, Andor Revolution XDi SDM was used and VGluT1/VGluT2 were labeled with Cy5/Pacific Blue and excited using 640/405 nm wavelengths, respectively. Quantification of the juxtapositions of PSD95 and SAP102 with those of VGluT1 and VGluT2 was done using Imaris software (version 8.1.2). Spot detection function was used to segment synapses expressing VGluT1, PSD95 and SAP102 puncta in 3D datasets (Z stack depth of 2 μm with 11 slices separated by 0.2 μm) from three mice (males, 4.7 months). Three-way colocalization was then measured by Imaris Triple Colocalization plugin, developed by Michael Adams, using a distance threshold of 600 nm. Colocalization with VGluT2 was measured in two steps: first, PSD95 and SAP102 spots with an intensity above 3,000 in VGluT2 channels were filtered. Second, colocalization between PSD95 and SAP102 within the filtered spots was measured using Imaris XT Colocalize Spots function.

#### Striatal Compartment Synaptome Parameters

For characterization of striatal compartments, PSD95/SAP102/MOR were labeled and imaged in the CP using Andor Revolution XDi SDM. Pairs of closely located images from each compartment were then analyzed using the Imaris software (version 8.1.2) in brain slices of three mice (males, 2-3 months). PSD95 and SAP102 were detected using spot detection in order to extract the number of puncta, mean punctum intensity and mean punctum size in each image. Colocalization was then measured as for synaptome mapping, using a distance threshold of 300 nm. Moreover, to consider the level of colocalization that might occur by chance, colocalization was also calculated between PSD95 and SAP102 images coming from different mice, in order to create a randomized control.

#### Similarity Matrices, Connectome, and Network Analysis

Each column/row in the similarity matrix in Figure 2F represents one delineated brain subregion shown in Figure 2C. Elements in the matrix are similarities between pairs of subregions, and calculated based on the Euclidean distance (Wang et al., 2005) between standardized synaptome parameter sets, each of which consists of punctum density, intensity, size, skewness, kurtosis, circularity, aspect ratio and colocalization percentage. A conventional Gaussian kernel function (Babaud et al., 1986) was finally applied to convert distance to similarity. The dendrogram in Figure 2G was plotted based on the similarity measurement in Figure 2F: the measurement was clustered hierarchically using unweighted average distance algorithm (Zhang et al., 2013). The similarity matrices in Figure 7 were also built using same method. The similarity matrix in Figure S17 was built as described in Figure 2F, except that matrix elements were calculated as Pearson correlation coefficients between densities of 37 subtypes in two delineated subregions.

The projection density of the structural mesoscale connectome shown in Figure 4B was calculated (Rubinov et al., 2015) as division of the projection strength by the volumetric size of the source region. All synaptome network analysis in Figures 4C–4G was based on the similarity matrix in Figure 2F. The nodes used in Figure 4C-E of the brain network analysis refer to the brain subregions defined in the ARA (Dong, 2008). The node degree of a brain subregion quantifies the average connectivity to other brain regions in the functional connectome by rs-fMRI (Stafford et al., 2014) and the average similarity to other brain regions in our synaptome data. The clustering coefficient (CC) in Figure 4C is calculated (Watts and Strogatz, 1998) as the fraction of triangles around a node and small-world networks usually have high clustering compared with random network (Bullmore and Sporns, 2009). The modularity in Figure 4C measures the degree to which the network can be decomposed into a set of non-overlapping subnets, each of which comprises a number of densely inter-connected nodes that are sparsely connected to the noses in the other subnets (Jao et al., 2015). The small worldness (Bullmore and Sporns, 2009) in Figure 4C is calculated as the ratio of the clustering coefficient and average path length normalized by the random network,

$$\sigma =\left({\text{C}}_{\text{net}}/{\text{C}}_{\text{rand}}\right)/\left(l_{\text{net}}/l_{\text{rand}}\right).$$

where *C*_net_, *C*_rand_ represents the CCs of the synaptome and random network respectively, and the *l*_net_, *l*_rand_ are the path length of the synaptome and random network, respectively.

#### Region Size and Cell Density

In order to check for gross anatomical changes in *Psd93*^−/−^ and *Sap102*^−/−^ mice compared to WT mice, the size of analyzed overarching areas was measured by adding together the surface area of delineated subregions that belong to this area. Moreover, cell density was measured from DAPI counterstaining in the same sections used for synaptome mapping. The DAPI signal was imaged at low resolution (20 × ) using the AxioScan.Z1 (Zeiss). Images were then exported in TIFF format and processed in Fiji. First, a background subtraction step was applied using a rolling ball radius of 50 pixels. Images were then scaled by 0.302 and Image-based Tool for Counting Nuclei (ITCN) plugin was applied using a width of 7 pixels and a minimum distance of 4 pixels. The number of counted nuclei was then normalized to subregion area in order to obtain cell density.

#### Cohen’s d Formula

Cohen’s *d* values were used to measure the effect size of synaptome parameter changes between WT and mutant mice as follows:

$$d=\frac{\bar{x_{1}}-\bar{x_{2}}}{\text{s}}$$

where $\bar{x_{1}}$ and $\bar{x_{2}}$ are the average synaptome parameter for the mutant and WT groups, respectively, for a given subregion, and s is pooled standard deviation, defined as follows:

$$s=\sqrt{\frac{\left(n_{1}-1\right)s_{1}^{2}+\left(n_{2}-1\right)s_{2}^{2}}{n_{1}+n_{2}-2}}$$

where s_1_ and s_2_ are the standard deviations in mutant and WT groups, respectively, and n_1_ and n_2_ are the N numbers of mutant and WT groups, respectively.

#### Bayesian Analysis

Bayesian estimation was used for the analysis of mutant effects on synaptome maps (Figure 7). Posterior probabilities of model parameters were estimated from the data and these were then used to determine whether genotype dependent differences exist. Each of the synaptome mapping parameters (e.g., intensity, density, and size) was modeled as having a t-distribution. For both studies, major brain regions (i.e., hippocampus, cortex, medulla, and striatum) were modeled separately, while subregions (i.e., CA1so, CA1slm and DGgrInf were contained within a single model).

The model was written in JAGS based on the script ‘Jags-Ymet-Xnom2fac-MrobustHet.R’ associated with Chapter 20.4 of Kruschke’s textbook (Kruschke, 2014). The key modification made to the base model is that the scale parameter of the t-distribution was adapted to depend only on one of the explanatory variables, ${\text{x}}_{2}$ (brain region) and not ${\text{x}}_{1}$ (genotype). All priors were left as set in the Kruschke model. The standard deviation of the t-distribution, $\sigma_{j}$, was permitted to vary across brain regions (indexed by *j*) but not with genotype, and was modeled as being gamma distributed with mode ω and standard deviation $\sigma_{\sigma }$. The values of ω and $\sigma_{\sigma }$ were themselves gamma distributed, with parameter pairs $\left\{Mo_{\omega },S_{\omega }\right\}$ and $\left\{Mo_{\sigma_{\sigma }},S_{\sigma_{\sigma }}\right\}$ defined based on the standard deviations found within region/genotype groups. The modes of ω and $\sigma_{\sigma }$ ($Mo_{\omega }$ and $Mo_{\sigma_{\sigma }}$) was thus set equal to the median standard deviation of region/genotype groups, while $S_{\omega }$ and $S_{\sigma_{\sigma }}$ were equal to the standard deviation of group standard deviations.

To evaluate differences between groups it was necessary to calculate the posterior probability for the mean of the t-distribution for each region and genotype. To approximate traditional significance testing we calculated a p value based on the maximal size for the Highest Density Region (HDI) before it would contain part of the Region of Practical Equivalence centered around zero. The Region of Practical Equivalence had boundaries defined as $\pm \sigma_{\text{j}}/3$. As several models were generated for the major brain regions (i.e., hippocampus and neocortex), Benjamini-Hochberg testing was used to correct over all probabilities.

### Data and Software Availability

#### Software

MATLAB package GraphVar used for the analysis of brain network is available at http://www.rfmri.org/GraphVar. Custom-built R code for statistical hypothesis test can be downloaded at http://synaptome.genes2cognition.org/download_source.html. Custom-built MATLAB code for electrophysiological modeling can be downloaded at http://synaptome.genes2cognition.org/download_source.html.

To visualize individual puncta overlaid on delineated section images we developed an in-house visualization software, called “Synaptome Explorer,” implemented in C++/OpenGL. The software uses several inputs: a) A stitched, downsampled section image generated from the raw microscopic images of each of the PSD95/SAP102 scans, b) a pre-processed form of all calculated puncta and their parameters, c) user-defined delineations provided as black-and-white overlays to the section composite image and d) a text file describing the delineation hierarchy. The software allows interactive exploration of the mouse synaptome at individual synapse resolution, utilizing an intuitive user interface resembling mouse-driven navigation functionality (panning, zooming) in mapping software such as Google Maps. All delineation overlays (> 700) are efficiently packed so that any individual or group of delineations can be visualized on the fly. Individual synaptic puncta can be displayed on top of the section image, filtered by proteins, types or subtypes, so that users can observe the spatial distribution of any possible subset of subtypes as well as the spatial relationships of puncta types/subtypes in any particular region. The tool is compiled as a standalone executable software including a manual file and can be downloaded at https://github.com/SynaptomeMapping/SynaptomeExplorer/archive/master.zip

#### Data

The full-resolution version of all synaptome maps presented in the paper are accessible at http://synaptome.genes2cognition.org/. Raw PSD95 and SAP102 fluorescence microscopy tiff images are very large (∼10 Terabytes) and therefore are available upon request by contacting Seth Grant, seth.grant@ed.ac.uk. Synaptome data (∼5Gb) of individual synaptic puncta used in the manuscript can be downloaded at https://doi.org/10.7488/ds/2366. The data include parameters of billions of individual synapses across the 5 coronal section, including the position, intensity, size, shape, type, and subtype for each individual synaptic punctum. They can be loaded and visualized with the Synaptome Explorer. An instructional video is given by synaptome_explorer.mp4 and a detailed manual file is included the software package.

Regional densities, sizes, and colocalization for SAP102 and PSD95 presented in Figure 2B can be found in Table S1. Raw values of the similarity matrix presented in Figure 2F can be found at Table S2. Tangential and Radial gradient presented in Figure 5 can be found at Table S3. Electrophysiological modeling data presented in Figure 6 can be found at Table 1 in section Computational modeling, above. PSD95 parameters quantified for the mutation experiments in Figure 7 can be found at Table S4.

**Table 1:** STP Parameters

	A_d0_	τ_d0_	A_f0_	τ_f0_	A_s0_	τ_s0_
control	0.25	130	4.9	13	0.45	860
PSD95	0.022	140	7.3	13	0.45	860
PSD93	0.14	40	4.4	13	0.45	860

## Supporting Citations

The following references appear in the Supplemental Information: Colledge et al., 2003, Morabito et al., 2004.

## Supplemental Information

Document S1. Figures S1–S20

Table S1. The PSD95 and SAP102 Synaptome Parameters in Different Brain Subregions on the Whole-Mouse-Brain Scale, Related to Figure 2B Average PSD95 and SAP102 punctum density, intensity, size, and colocalization values in different delineated brain subregions defined in the Allen Reference Atlas. The main overarching area is also indicated for each subregion. Abbreviations, see Table S2.

Table S2. The Similarity Matrix of the Synaptome Maps, Related to Figure 2F Each row and column in the table represent one delineated mouse brain subregion and values in the cell represent the similarity of synaptome parameters for the two subregions. Abbreviations: ACAd, Anterior Cingulate Area, dorsal part; ACAv, Anterior Cingulate Area, ventral part; ACB, nucleus Accumbens; AId, Agranular Insular area, dorsal part; AIv, Agranular Insular area, ventral part; AN, Anisoform lobule; ANcr1gr, Anisoform lobule, crus 1, granular layer ; ANcr1mo, Anisoform lobule, crus 1, molecular layer ; ANcr2gr, Anisoform lobule, crus 2, granular layer ; ANcr2mo, Anisoform lobule, crus 2, molecular layer ; AON, Anterior Olfactory Nucleus; AON1l, Anterior Olfactory Nucleus, layer 1, lateral part; AON1m, Anterior Olfactory Nucleus, layer 1, medial part; AONm, Anterior Olfactory Nucleus, medial part; AONpv, Anterior Olfactory Nucleus, posteroventral part ; ARH, Arcuate Hypothalamic nucleus; AUDd, Auditory area, dorsal part; AUDp, Auditory area, primary part; AUDv, Auditory area, ventral part; BLA, Basolateral Amygdalar nucleus; BLAa, Basolateral Amygdalar nucleus, anterior part; BLAp, Basolateral Amygdalar nucleus, posterior part; BLAv, Basolateral Amygdalar nucleus, ventral part; BMA, Basomedial Amygdalar nucleus; BMAp, Basomedial Amygdalar nucleus, posterior part; CA1, Cornu Ammonis 1; CA1slm, Cornu Ammonis 1, stratum lacunosum-moleculare; CA1so, Cornu Ammonis 1, stratum oriens; CA1sp, Cornu Ammonis 1, stratum pyramidale; CA1sr, Cornu Ammonis 1, stratum radiatum; CA2, Cornu Ammonis 2; CA2slm, Cornu Ammonis 2, stratum lacunosum-moleculare; CA2so, Cornu Ammonis 2, stratum oriens; CA2sp, Cornu Ammonis 2, stratum pyramidale; CA2sr, Cornu Ammonis 2, stratum radiatum; CA3, Cornu Ammonis 3; CA3slm, Cornu Ammonis 3, stratum lacunosum-moleculare; CA3slu, Cornu Ammonis 3, stratum lucidum; CA3so, Cornu Ammonis 3, stratum oriens; CA3sp, Cornu Ammonis 3, stratum pyramidale; CA3sr, Cornu Ammonis 3, stratum radiatum; CEA, Central Amygdalar nucleus; CENT, Central lobules; CENT1gr, Central lobule I, granular layer; CENT1mo, Central lobule I, molecular layer; CENT2gr, Central lobule II, granular layer; CENT2mo, Central lobule II, molecular layer; CENT3gr, Central lobule III, granular layer; CENT3mo, Central lobule III, molecular layer; CLA, Claustrum; CM, Central Medial nucleus of the thalamus; CNlam, granular lamina of the Cochlear Nuclei; COAp, Cortical Amygdalar area, posterior part; COApl, Cortical Amygdalar area, posterior part, lateral zone; COApl1, Cortical Amygdalar area, posterior part, lateral zone, layer 1; COApl2, Cortical Amygdalar area, posterior part, lateral zone, layer 2; COApl3, Cortical Amygdalar area, posterior part, lateral zone, layer 3; COApm1, Cortical Amygdalar area, posterior part, medial zone, layer 1; COApm2, Cortical Amygdalar area, posterior part, medial zone, layer 2; COApm3, Cortical Amygdalar area, posterior part, medial zone, layer 3; CP, Caudate Putamen; CUL, Culmen lobules ; CUL4,5gr, Culmen lobules IV-V, granular layer; CUL4,5mo, Culmen lobules IV-V, molecular layer; DCO, Dorsal Cochlear nucleus; DCOmo, Dorsal Cochlear nucleus, molecular layer; DG, Dentate Gyrus; DG-sg inf, Dentate Gyrus, granular layer, inferior blade; DG-sg sup, Dentate Gyrus, granular layer, superior blade; DG-sg vent, Dentate Gyrus, granular layer, ventral part; DG-mo inf, Dentate Gyrus, molecular layer, inferior blade; DG-mo sup, Dentate Gyrus, molecular layer, superior blade; DG-mo vent, Dentate Gyrus, molecular layer, ventral part; DG-po, Dentate Gyrus, polymorphic cell layer ; DG-po vent, Dentate Gyrus, polymorphic cell layer, ventral part; DMH, Dorsomedial nucleus of the Hypothalamus; DN, Dentate Nucleus; ECT, Ectorhinal area; ENTl, Entorhinal area, lateral part; EPd, Endopiriform nucleus, dorsal part; EPv, Endopiriform nucleus, ventral part; FC, Fasciola Cinerea ; FL, Flocculus; FLgr, Flocculus, granular layer; FLmo, Flocculus, molecular layer; GU, Gustatory areas; IA, Intercalated Amygdalar nucleus; IG, Induseum griseum; ILA, Infralimbic area; IMD, Intermediodorsal nucleus of the thalamus; IP, Interposed nucleus; isl, islands of Calleja; LA, Lateral Amygdalar nucleus; LAV, Lateral Vestibular nucleus; LC, Locus Ceruleus; LD, Lateral Dorsal nucleus of thalamus; LG, Lateral Geniculate complex; LGv, ventral part of the Lateral Geniculate complex; LH, Lateral Habenula; LHA, Lateral Hypothalamic area; LP, Lateral Posterior nucleus of the thalamus; LSc, Lateral Septal nucleus, caudal part; LSr, Lateral Septal nucleus, rostral part; LT, Lateral Terminal nucleus of the accessory optic tract; MBmot, Midbrain, motor related; ME, Medial Eminence; MEA, Medial Amygdalar nucleus; MG, Medial Geniculate complex; MH, Medial Habenula; MM, Medial Mammillary nucleus; MOp, Primary motor area; MOs, secondary Motor area; MS, Medial Septal nucleus; MSC, Medial Septal Complex; MV, Medial Vestibular nucleus; MY-mot, Medulla, motor related; ND, Nucleus of Darkschewitsch; ORBl, Orbital area, lateral part; ORBm, Orbital area, medial part; ORBvl, Orbital area, ventrolateral part; OT, Olfactory Tubercle; OT1, Olfactory Tubercle, molecular layer; OT2, Olfactory Tubercle, pyramidal layer; OT3, Olfactory Tubercle, polymorph layer; PA, Posterior Amygdalar nucleus; PAA, Piriform-Amygdalar Area; PAA1, Piriform-Amygdalar Area, molecular layer; PAA2, Piriform-Amygdalar Area, pyramidal layer; PAA3, Piriform-Amygdalar Area, polymorph layer; PAG, Periaqueductal Gray; PERI, Perirhinal area; PF, Parafascicular nucleus; PFL, Paraflocculus; PFLgr inf, Paraflocculus, granular layer, inferior part; PFLgr sup, Paraflocculus, granular layer, superior part; PFLmo inf, Paraflocculus, molecular layer, inferior part; PFLmo sup, Paraflocculus, molecular layer, superior part; PIR, Piriform area; PIR1, Piriform area, layer 1; PIR2, Piriform area, layer 2; PIR3, Piriform area, layer 3; PL, Prelimbic area; PO, Posterior complex of the thalamus; POL, Posterior Limiting nucleus of the thalamus; PP, Peripeduncular nucleus; PRP, nucleus Prepositus; PSTN, Parasubthalamic Nucleus; PTLp, Posterior parietal association areas; PVT, Paraventricular nucleus of the thalamus; RAmb, midbrain Raphe nuclei; RM, Nucleus raphe magnus; RPA, nucleus Raphe Pallidus; RSPagl, Retrosplenial area, lateral agranular part; RSPd, Retrosplenial area, dorsal part; RSPv, Retrosplenial area, ventral part; RT, Reticular nucleus of the thalamus; SCs, Superior Colliculus, sensory related; SCzo, Superior Colliculus, zonal layer; SH, Septohippocampal nucleus; SI, Substantia Innominata; SIM, Simple lobule; SIMgr, Simple lobule, granular layer; SIMmo, Simple lobule, molecular layer; SNc, Substantia Nigra, compact part; SNr, Substantia Nigra, reticular part; SPFm, Subparafascicular nucleus, magnocellular part; SPFp, Subparafascicular nucleus, parvicellular part; SPVO, Spinal nucleus of the trigeminal, Oral part; SPVOrdm, Spinal nucleus of the trigeminal, Oral part, rostral dorsomedial part; SPVOvl, Spinal nucleus of the trigeminal, Oral part, ventrolateral part; SSp-bfd, primary Somatosensory area, barrel field; SSp-m, primary Somatosensory area, mouth; SSp-tr, primary Somatosensory area, trunk; SSp-ul, primary Somatosensory area, upper limb; STN, Subthalamic Nucleus; SUBd, Subiculum, dorsal part; SUBd-m, Subiculum, dorsal part, molecular layer; SUBd-sp, Subiculum, dorsal part, pyramidal layer; SUBd-sr, Subiculum, dorsal part, stratum radiatum; SUM, Supramammillary nucleus; SUV, Superior Vestibular nucleus; TEa, Temporal association areas ; TR, postpiriform Transition area; TR1, postpiriform Transition area, layer 1; TR2, postpiriform Transition area, layer 2; TR3, postpiriform Transition area, layer 3; TT, Taenia Tecta; TTd1, Taenia Tecta, dorsal part, layer 1; TTd3, Taenia Tecta, dorsal part, layer 3; TTv1, Taenia Tecta, ventral part, layer 1; TTv2, Taenia Tecta, ventral part, layer 2; TTv3, Taenia Tecta, ventral part, layer 3; TU, Tuberal nucleus; VCO, Ventral Cochlear nucleus; VISal, anterolateral Visual area; VISam, Anteromedial Visual area; VISp, primary Visual area; VM, Ventral Medial nucleus of the thalamus; VPL, Ventral Posterolateral nucleus of the thalamus; VPLpc, Ventral Posterolateral nucleus of the thalamus, parvicellular part; VPM, Ventral Posteromedial nucleus of the thalamus; VPPC, Ventral posterior nucleus, parvicellular part; ZI, Zona Incerta; ZID, Zona Incerta, dorsal part; ZIV, Zona Incerta, ventral part.

Table S3. The PSD95 and SAP102 Gradients in the CA1SR of the Hippocampus, Related to Figure 5B The PSD95 and SAP102 intensity and size parameters along the tangential (the first spreadsheet) and radial axis (the second spreadsheet) in the CA1SR of the hippocampus.

Table S4. PSD95 Synaptome Parameters in Wild-Type, *Psd93*^−/−^, and *Sap102*^−/−^ Mice, Related to Figure 7A Average PSD95 punctum density (left tables, in puncta per 100 μm^2^), intensity (middle tables, mean gray value, 16 bits), and size (right tables, in μm^2^) in wild-type (orange, n = 13), *Psd93*^−/−^ (blue, n = 6) and *Sap102*^−/−^ mice (green, n = 11). The standard deviations (STD), the Cohen’s d values (effect size between wild-type and mutant group, see STAR Methods for details) and the p values measured using the Bayesian estimation method are also indicated. p values are highlighted as follows: 0.01 < p < 0.05 in yellow and p < 0.01 in red. Results are indicated separately for the 9 overarching areas (top tables) and for the 76 subregions (bottom tables) studied. Results for *Psd93*^−/−^ mice are in the first sheet, and results for *Sap102*^−/−^ mice are in the second sheet. Region abbreviations are consistent with those in Table S1.

Document S2. Article plus Supplemental Information
