# Supplementary material for: Architecture of the Mouse Brain Synaptome
Source: Neuron. 2018 Aug 22;99(4):781–799.e10. doi: 10.1016/j.neuron.2018.07.007 (PMC6117470; doi:10.1016/j.neuron.2018.07.007)
Supplement: Document S1. Figures S1–S20 [file mmc1.pdf]

Neuron, Volume *99*

## Supplemental Information

# Architecture of the Mouse Brain Synaptome

**Fei Zhu, Mélissa Cizeron, Zhen Qiu, Ruth Benavides-Piccione, Maksym V. Kopanitsa, Nathan G. Skene, Babis Koniaris, Javier DeFelipe, Erik Fransén, Noboru H. Komiyama, and Seth G.N. Grant**

# Supplementary Figures (S1-20)

## Figure S1.

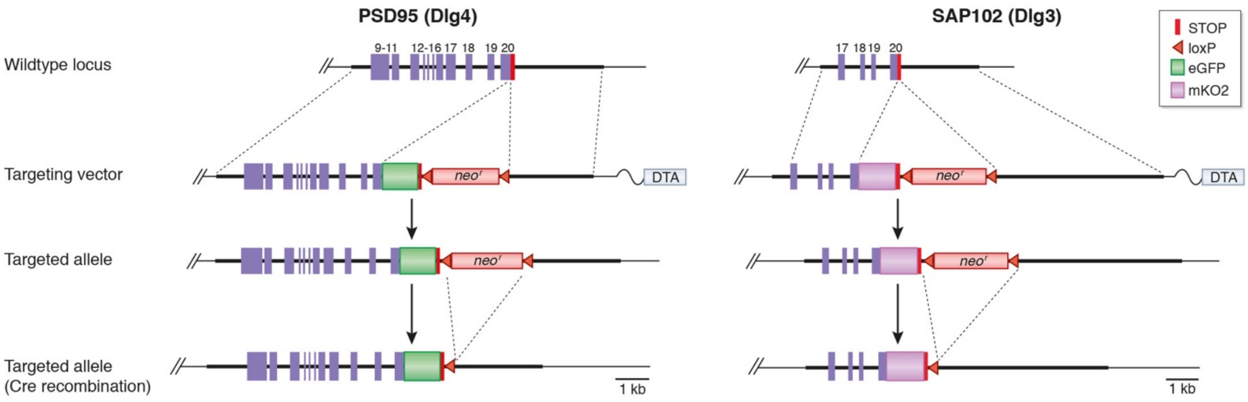


**Figure S1 related to Figure 1. Gene targeting strategy for generation of PSD95-eGFP and SAP102-mKO2 mice.**

The wildtype genomic loci of murine *Dlg4* (PSD95) and *Dlg3* (SAP102) genes showing exon structure and STOP codon is shown with the corresponding Targeting vectors (dotted lines show regions of homology). The vectors contain a cDNA encoding their respective fluorescent protein (eGFP, mKO2), a neomycin positive selection cassette (*neo*$^r$) flanked by LoxP sites and a Diptheria Toxin A (DTA) negative selection cassette. Homologous recombination of the targeting vector with the wildtype locus results in integration of the sequence encoding the fluorescent protein and the neomycin selection cassette into the genome (targeted allele). The *loxP*-flanked neomycin cassette was removed by crossing with Cre-deleter mice.

**Figure S2.**

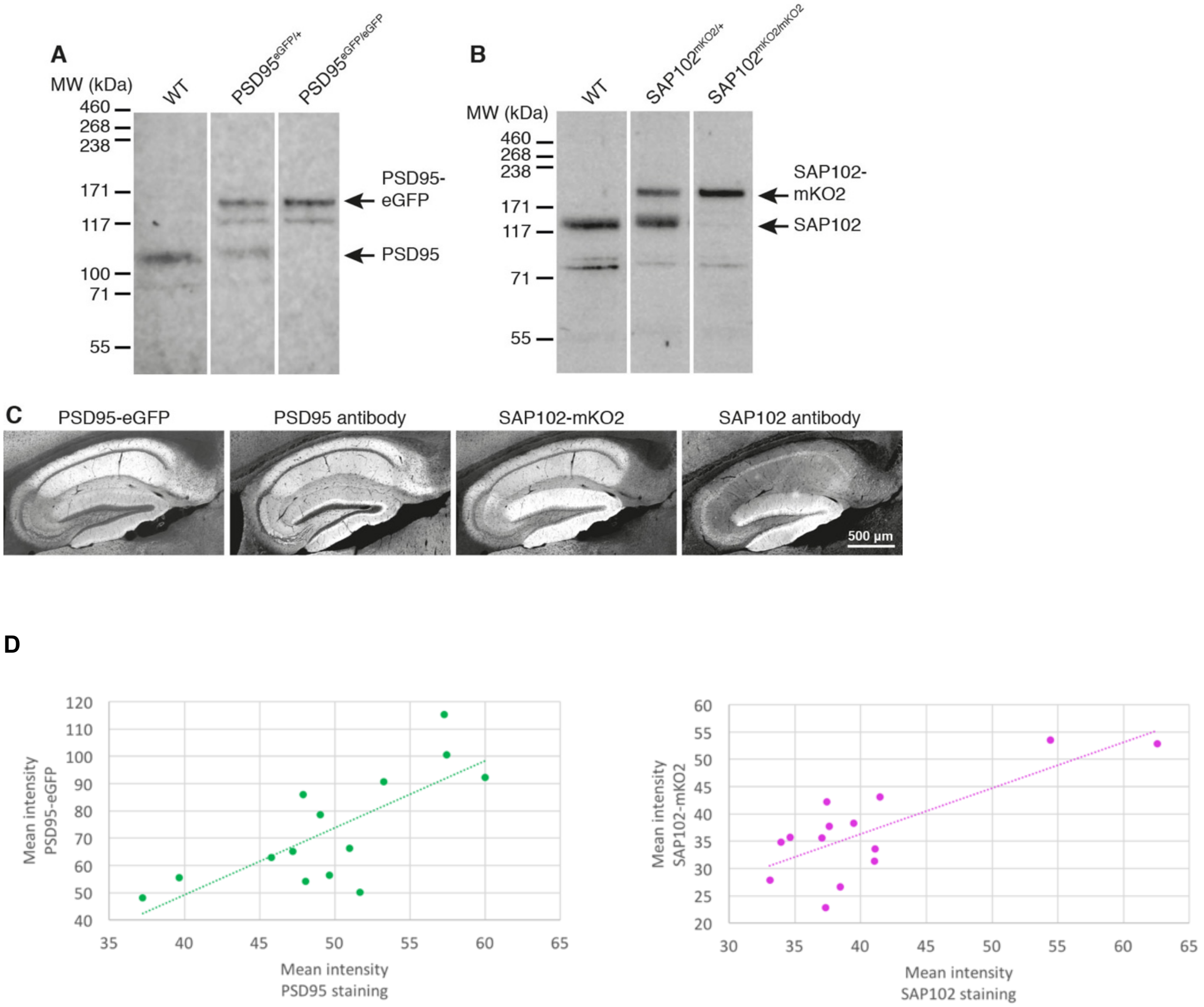


**Figure S2 related to Figure 1. Normal expression of PSD95-eGFP and SAP102-mKO2 proteins.**

A-B. Western blot analysis of forebrain extracts from wild-type, heterozygous and homozygous mice for the PSD95-eGFP (A) or SAP102-mKO2 (B) allele, using antibodies against PSD95 (A) or SAP102 (B). All lanes are from the same blot. Molecular weight (MW) indicated by size markers (kDa, kilodalton). Note that the lower band (~72 kD) in PSD95 blots has been reported previously (Morabito et al., 2004; Colledge et al, 2003; Frank et al, 2017) and is either a different isoform of PSD95 or an N-terminal cleavage product of PSD95. This product is also labelled by our PSD95-eGFP-knockin mice.

C. Comparable subregional patterns of labelling are obtained in the hippocampus between PSD95-eGFP and PSD95 antibody staining and between SAP102-mKO2 and SAP102 antibody staining. Scale bar: 500 μm. Note that immunolabelling of SAP102 requires antigen retrieval, which produces variability in the signal (Fukaya and Watanabe, 2000). The pattern of expression of PSD95-eGFP and SAP102-mKO2 is nonetheless very similar to previously published results (Fukaya and Watanabe, 2000).

D. Correlation between the expression intensity in hippocampal formation regions using immunohistochemistry and genetic labelling. A significant positive correlation between the two staining methods was observed (PSD95, R = 0.75, $P$ = 0.0022; SAP102, R = 0.77, $P$ = 0.0014).

**Figure S3.**

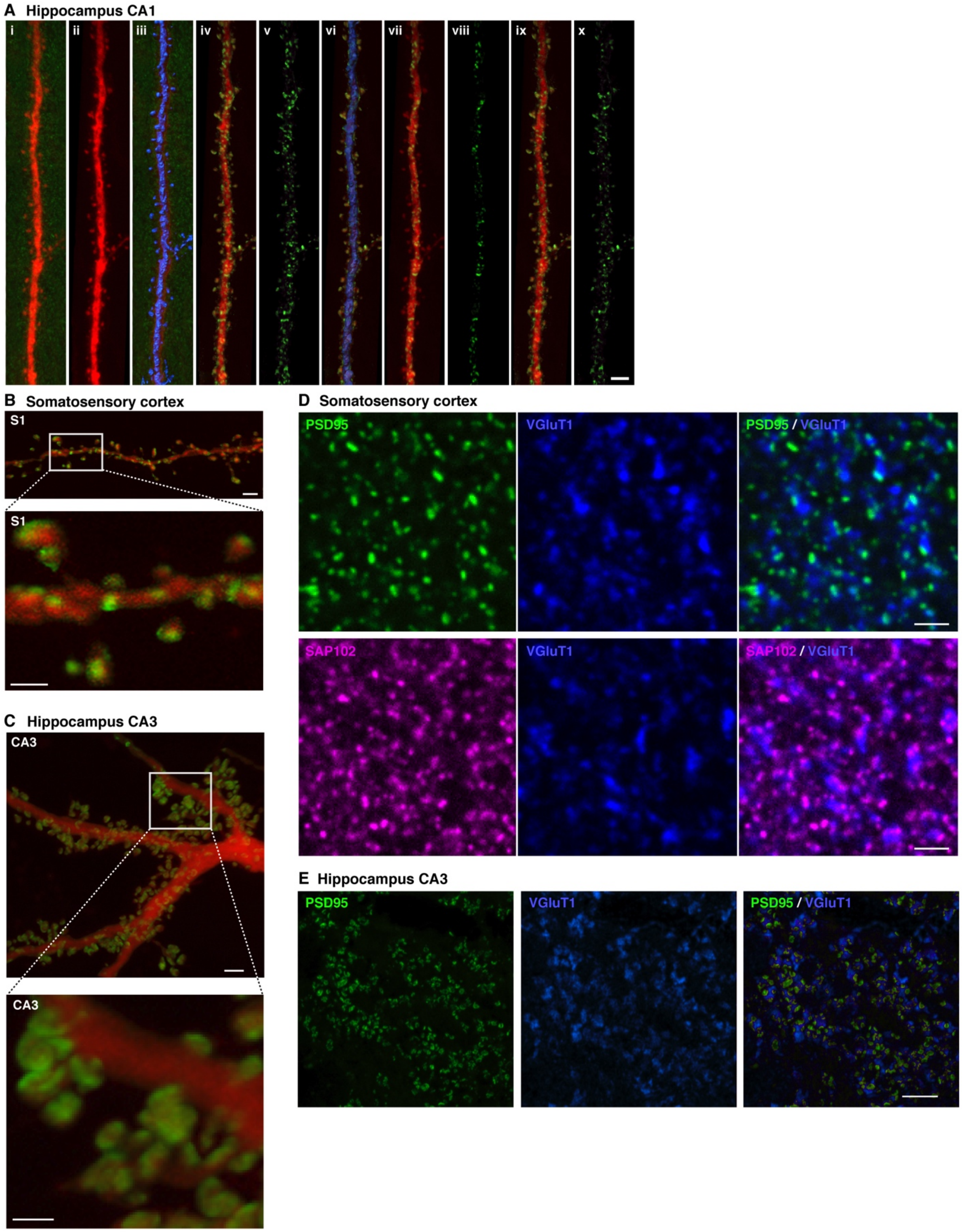
A Hippocampus CA1
i
ii
iii
iv
v
vi
vii
viii
ix
x
B Somatosensory cortex
S1
S1
C Hippocampus CA3
CA3
CA3
D Somatosensory cortex
PSD95
VGluT1
PSD95 / VGluT1
SAP102
VGluT1
SAP102 / VGluT1
E Hippocampus CA3
PSD95
VGluT1
PSD95 / VGluT1

**Figure S3 related to Figure 1. Postsynaptic localization of PSD95-eGFP and SAP102-mKO2 proteins in dendritic spines.**

A. Dendritic segment of CA1 pyramidal neuron (110–160 microns from the soma) filled with Alexa fluor 594 (red) showing the surface of the dendritic shaft and spines relative to the location of PSD95eGFP (green).

i. Image showing the Alexa fluor 594-injected apical dendritic segment (110–160 microns from the soma; red) and the punctuate PSD95 labelling (green).

ii. Same dendritic segment as in (i), showing only the information from the red channel.

iii. Shows the information in (i) but including an artificial surface that fitted (by thresholding) the surface of the real dendrite of both the dendritic shaft and spines (blue).

iv. Image processing to obtain a new green channel with the information contained within the dendritic surface created, by appling a mask to the green channel that set the voxels outside the surface to 0. Thus, by combining the new green channel and the real dendrite, the relative location of PSD95 (green) could be established with respect to the surface of the dendrite (red).

v. Shows the information in (iv) but including only the green channel.

vi. Shows the information in (iv) but including an artificial surface represented the estimated dendritic shaft volume (blue).

vii. Same image processing as in (iv) to obtain a new green channel with the information contained within the dendritic shaft, that reveals the relative location of PSD95 with respect to the dendritic shaft.

viii. Shows the information in (vii) but including only the green channel.

ix. Image processing to obtain a new green channel with the information contained on the surface of the dendritic spines, by applying a mask to the green channel that set the voxels on the surface representing the estimated dendritic shaft (shown in vii) to 0. Thus, by combining the new green channel and the real dendrite, the relative location of PSD95 (green) could be established with respect to the dendritic spines.

x. Shows the information in (ix) but including only the green channel**.** Scale Bar = 2.5 µm.

B. PSD95-eGFP (green) localization to spine heads in dye-filled dendrites from principal neurons (red) in the somatosensory cortex. Scale bars are 2 μm (left panel) and 1 μm (right panel).
C. PSD95-eGFP (green) localization to spine heads in dye-filled dendrites from principal neurons (red) in the CA3 subfield of the hippocampus. Note the large thorny excrescence synapses, typical of the CA3 stratum lucidum, compared to the more classical smaller spines in the cortex (panel B). Scale bars are 2 μm (left panel) and 1 μm (right panel).
D. PSD95-eGFP puncta (top left panel, green) and SAP102-mKO2 puncta (bottom left panel, magenta) are juxtaposed to the presynaptic terminal (VGluT1) puncta (middle panels, blue) as shown on merged images (right panels, top: PSD95+VGluT1, bottom: SAP102+VGLuT1) from layer 1 of the somatosensory cortex. Scale bar: 2 μm.
E. PSD95-eGFP puncta (green) of CA3 thorny excrescence synapses are juxtaposed to the presynaptic VGluT1 puncta (blue) as shown on merged image (PSD95+VGluT1). Scale Bar: 6 μm.

**Figure S4.**

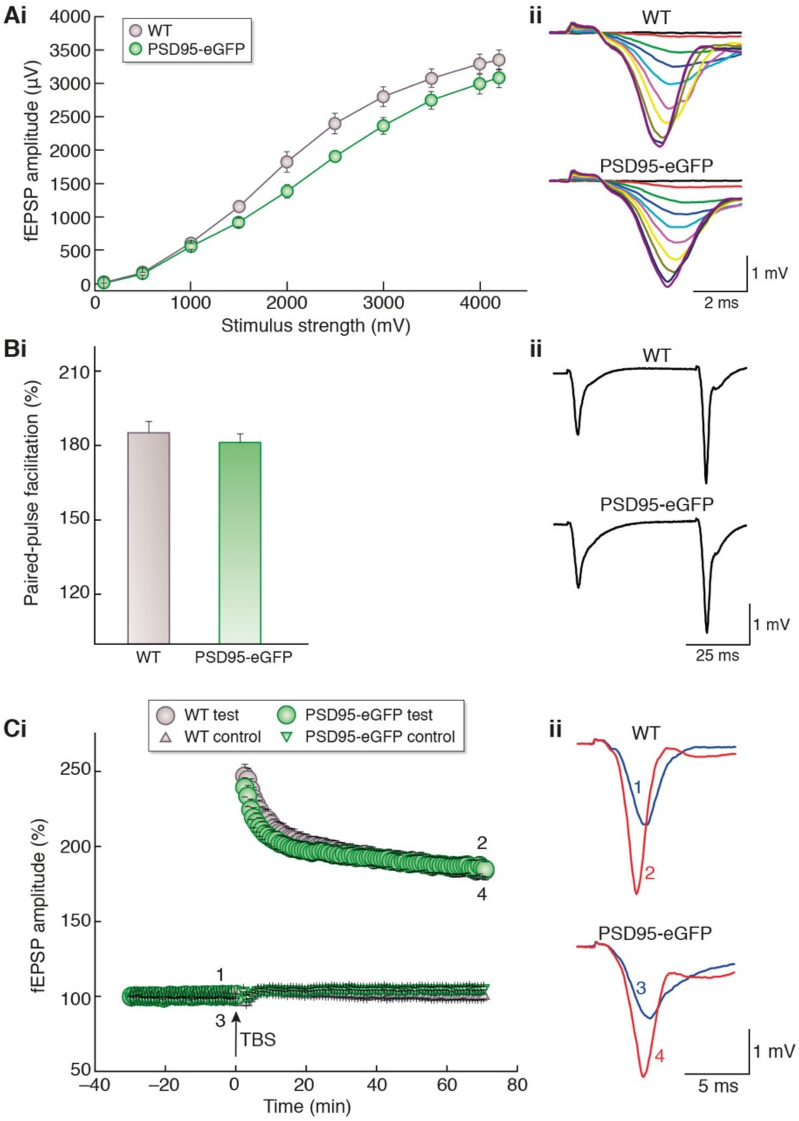


**Figure S4 related to Figure 1. Normal synaptic physiology in PSD95-eGFP mice.**

A. Input-output relationships (i) illustrate averaged fEPSP amplitudes in slices from PSD95-eGFP (n = 19; N = 6) and WT mice (n = 18; N = 6) in response to stimulation of Schäffer collaterals by biphasic voltage pulses of 0.1 – 4.2 V. Representative families of fEPSP traces are given in (ii). Comparison of areas under the input-output curve revealed no significant effect of genotype ($F_{(1,8.93)}$ = 3.019; $P$ = 0.117; two-way nested ANOVA). Similarly, maximum fEPSPs did not differ significantly in mutant and WT mice ($F_{(1,9.44)}$ = 0.79; $P$ = 0.396).

B. Paired-pulse facilitation (i) was not significantly different ($F_{(1,8.96)} = 0.351$; $P = 0.568$) in PSD95-eGFP animals ($n = 19$; $N = 6$) as compared to their WT littermates ($n = 18$; $N = 6$). Representative fEPSP sweeps are given in ii.

C. Theta-burst stimulation elicited pathway-specific long-term potentiation of synaptic transmission in hippocampal CA1 area (i). Normalised magnitude of this potentiation 60-65 min after LTP induction in mutant mice (178 ± 6%; $n = 19$; $N = 6$) and their wild type counterparts was not significantly different (187 ± 6%; $n = 18$; $N = 6$; $F_{(1,7.55)} = 1.48$; $P = 0.26$). Examples of test pathway fEPSP traces immediately before and 1 h after theta-burst stimulation are presented in (ii). Data are expressed as mean ± s.e.m. Statistical significance of differences of mean values between the two genotypes was determined by two-way nested ANOVA with Satterthwaite's correction (main genotype effect).

**Figure S5.**

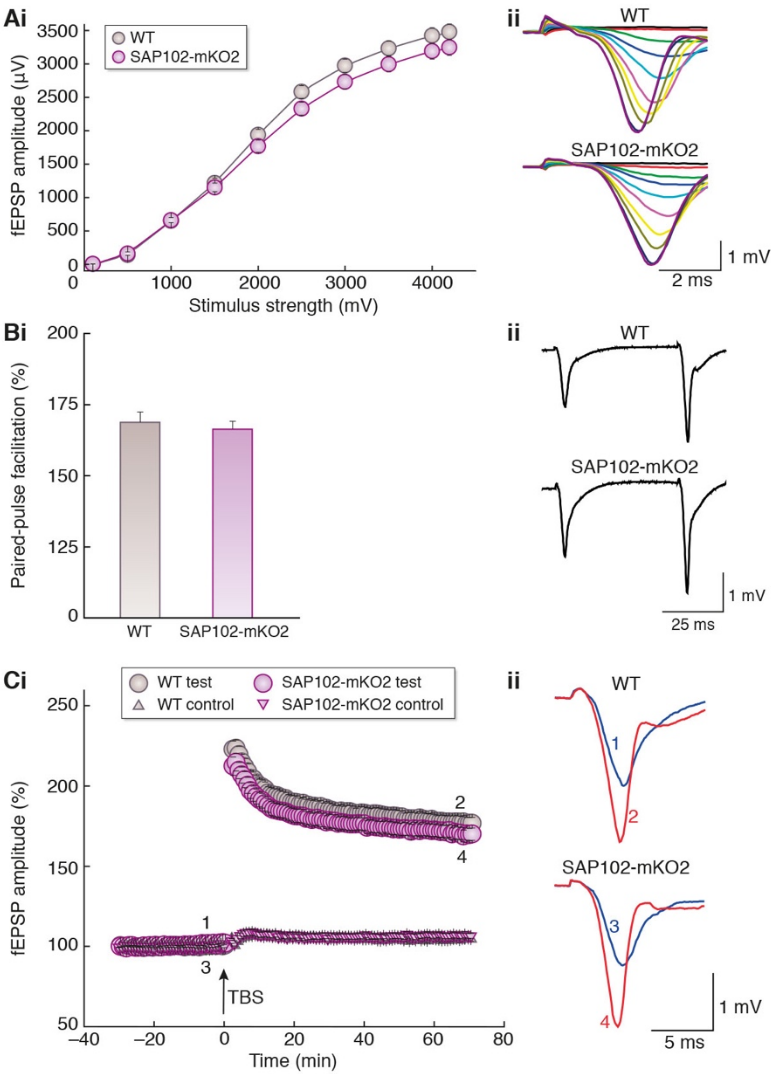


**Figure S5 related to Figure 1. Normal synaptic physiology in SAP102-mKO2 mice.**

A. Input-output relationships (i) illustrate averaged fEPSP amplitudes in slices from SAP102-mKO2 (n = 28; N = 7) and WT mice (n = 29; N = 7) in response to stimulation of Schäffer collaterals by biphasic voltage pulses of 0.1 – 4.2 V. Representative families of fEPSP traces are given in (ii). Comparison of areas under the input-output curve revealed no significant effect of genotype ($F_{(1,11.27)}$ = 1.83; $P$ = 0.203; two-way nested ANOVA). Similarly, maximum fEPSPs did not differ significantly in mutant and WT mice ($F_{(1,11.46)}$ = 1.44; $P$ = 0.255).

B. Paired-pulse facilitation (i) was not significantly different ($F_{(1,11.31)} = 0.253$; $P = 0.624$) in SAP102-mKO2 animals ($n = 28$; $N = 7$) as compared to their WT littermates ($n = 29$; $N = 7$). Representative fEPSP sweeps are given in (ii).

C. Theta-burst stimulation elicited pathway-specific long-term potentiation of synaptic transmission in hippocampal CA1 area (i). Normalized magnitude of this potentiation 60–65 min after LTP induction in mutant mice (161 ± 4%; $n = 24$; $N = 6$) and their wild type counterparts (171 ± 3%; $n = 29$; $N = 7$) was not significantly different ($F_{(1,10.44)} = 3.02$; $P = 0.111$). Examples of test pathway fEPSP traces immediately before and 1 h after theta-burst stimulation are presented in ii. Data are expressed as mean ± s.e.m. Statistical significance of differences of mean values between the two genotypes was determined by two-way nested ANOVA with Satterthwaite's correction (main genotype effect).

# Figure S6.

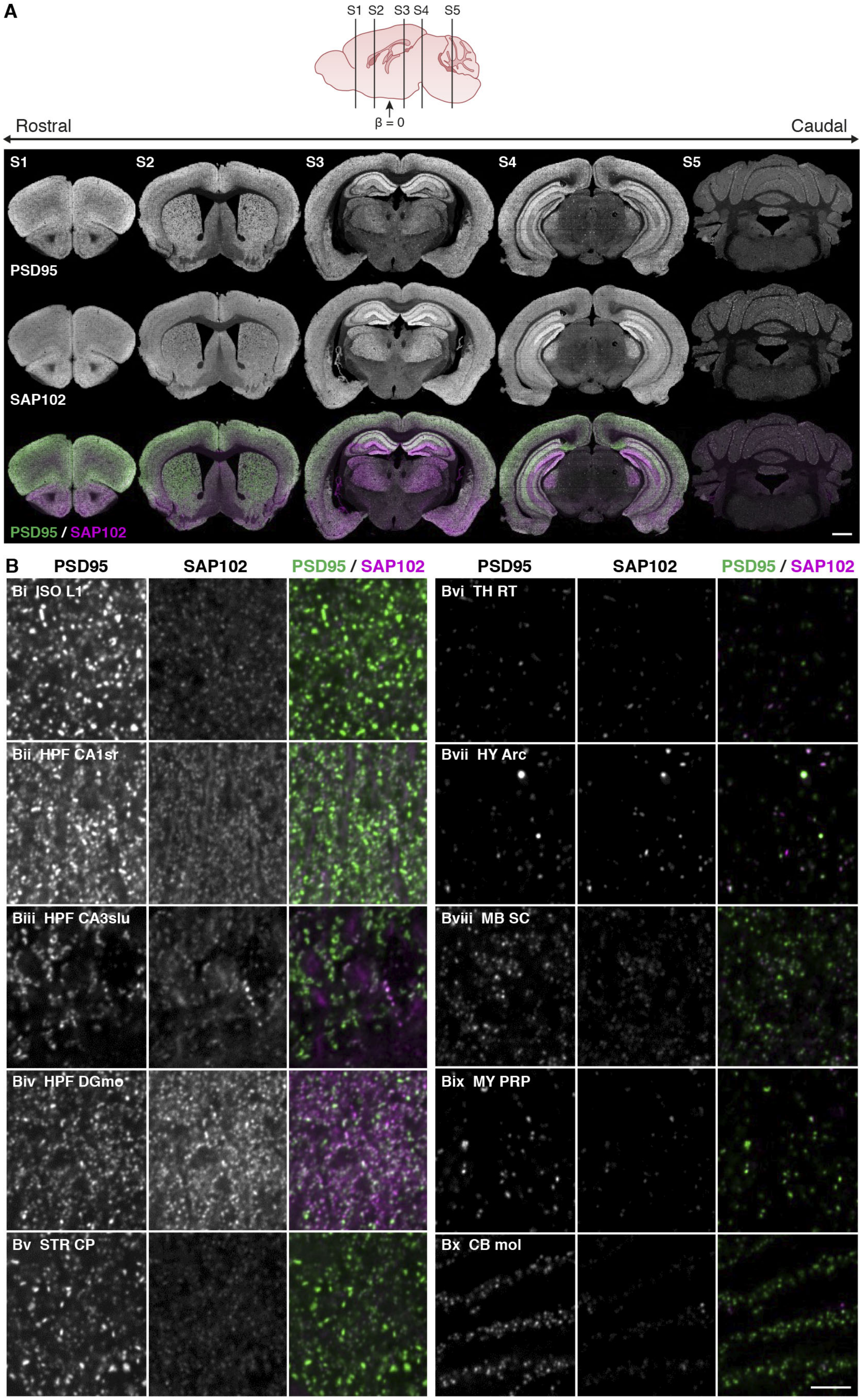
A
S1 S2 S3 S4 S5
β = 0
Rostral
Caudal
S1
S2
S3
S4
S5
PSD95
SAP102
PSD95 / SAP102
B
PSD95
SAP102
PSD95 / SAP102
PSD95
SAP102
PSD95 / SAP102
Bi ISO L1
Bii HPF CA1sr
Biii HPF CA3slu
Biv HPF DGmo
Bv STR CP
Bvi TH RT
Bvii HY Arc
Bviii MB SC
Bix MY PRP
Bx CB mol

**Figure S6 related to Figure 2. Comparison of expression of PSD95eGFP and SAP102mKO2**

A. As in Figure 2A, the expression pattern of PSD95 (grey in top row, green in bottom row) and SAP102 (grey in second row, magenta in bottom row) in the stitched down-sampled images from thousands of high-resolution (75 nm/pixel) images for 5 coronal sections: Section 1 (S1) is located at bregma (β) level +2.3 mm, Section 2 (S2) at β +0.8 mm, Section 3 (S3) at β -1.9 mm, Section 4 (S4) at β -3.1 mm, Section 5 (S5) at β -5.8 mm.
B. High-resolution images comparing the expression of PSD95-eGFP and SAP102-mKO2 puncta in different brain regions: isocortex (ISO), layer 1 (Bi); hippocampal formation (HPF), CA1 statum radiatum (CA1sr, Bii), CA3 stratum lucidum (CA3slu, Biii) and Dentate Gyrus molecular layer (DGmo, Biv); striatum (STR) caudate putamen (CP, Bv); thalamus (TH) reticular nucleus (RT, Bvi); hypothalamus (HY) arcuate nucleus (Arc, Bvii); midbrain (MB) superior colliculus (SC, Bviii); nucleus prepositus (Bix); cerebellum (CB), molecular layer (mol, Bx).

**Figure S7.**

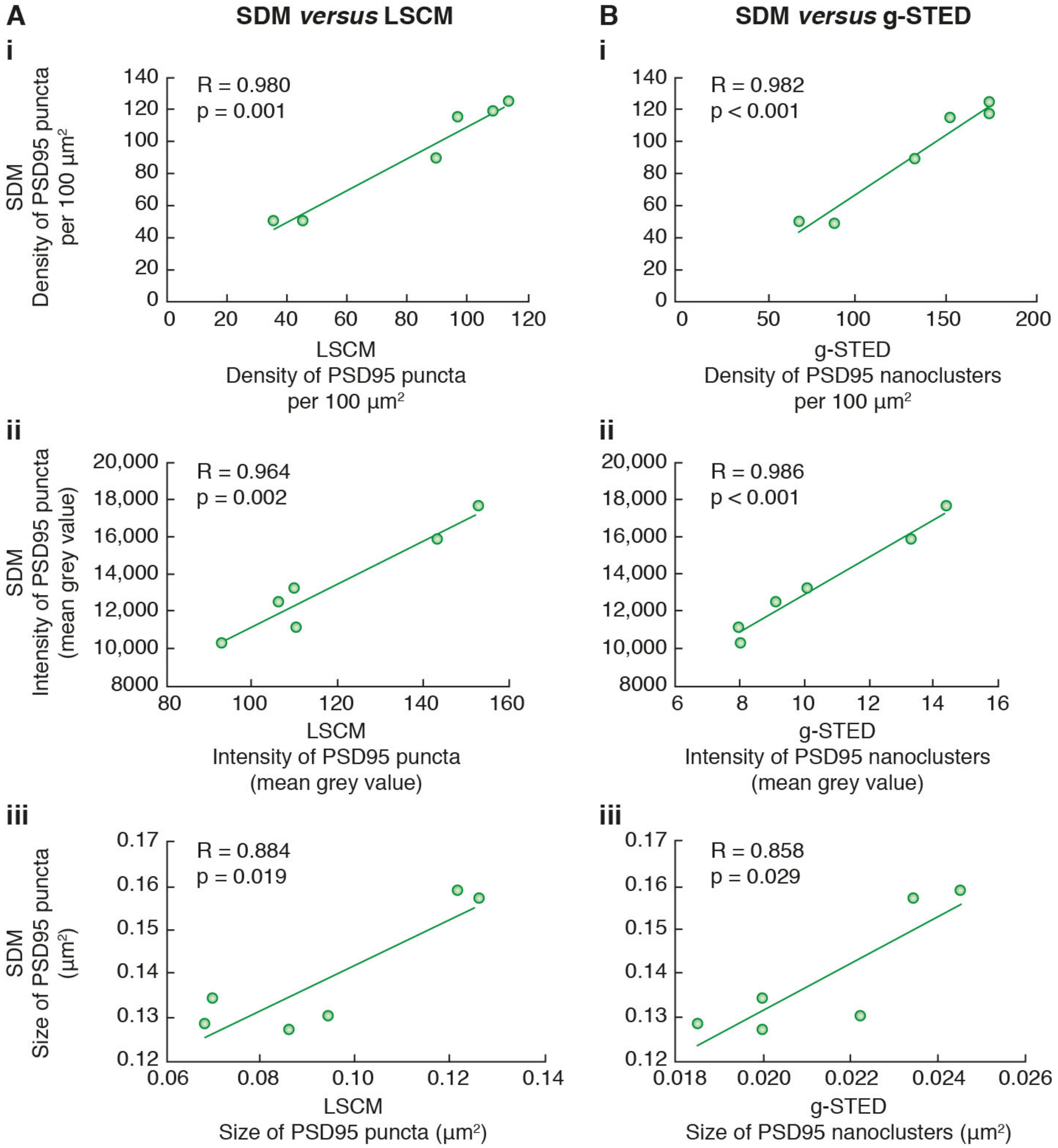


**Figure S7 related to Figure 2. Validation of Spinning Disk Confocal microscopy (SDM) quantifications.**

Correlations of PSD95-eGFP puncta parameters quantified from the Yokogawa CV1000 SDM with data (Broadhead et al., 2016) from the Leica SP5 Laser Scanning Confocal Microscope (LSCM, A) or Leica SP5 gated-Stimulation Emission Depletion (g-STED, B) systems. High positive correlations (see Pearson coefficient R for each panel) are

observed from both systems for the three main parameters: PSD95 punctum density (Ai, Bi), intensity (Aii, Bii) and size (Aiii, Biii).

**Figure S8.**

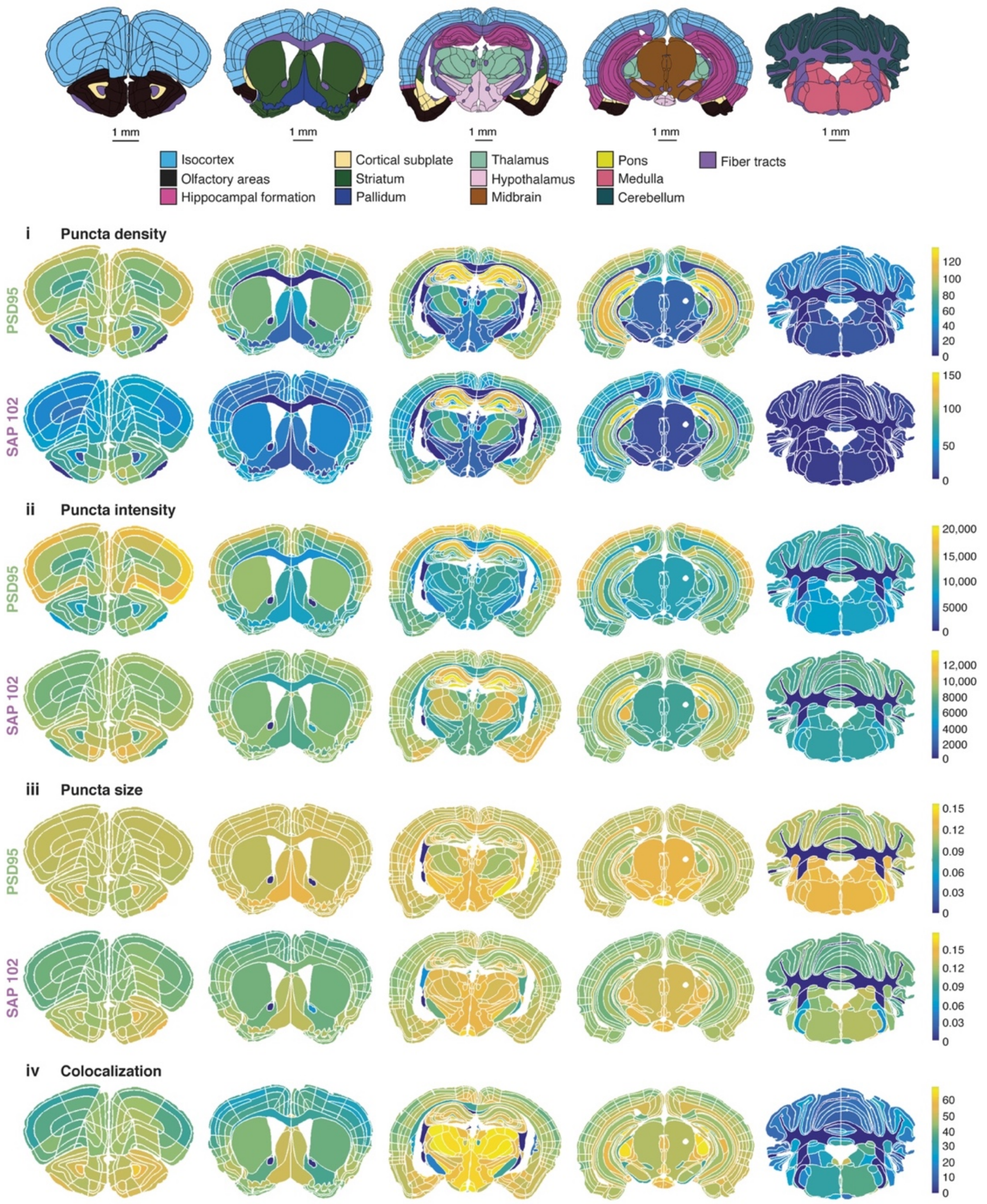


**Figure S8 related to Figure 2. Synaptome maps of PSD95 and SAP102**

A high-resolution image of synaptome maps from Figure 2C, which shows the spatial and anatomical distribution of median punctum density (i), intensity (ii), size (iii) and

colocalization (iv) for PSD95 (upper panels) and SAP102 (lower panels) in delineated subregions. Parameter units: density, number of puncta per 100 $\mu m^2$; intensity, mean grey value per punctum (AU $\times 10^4$); size, $\mu m^2$; colocalization, %.

**Figure S9.**

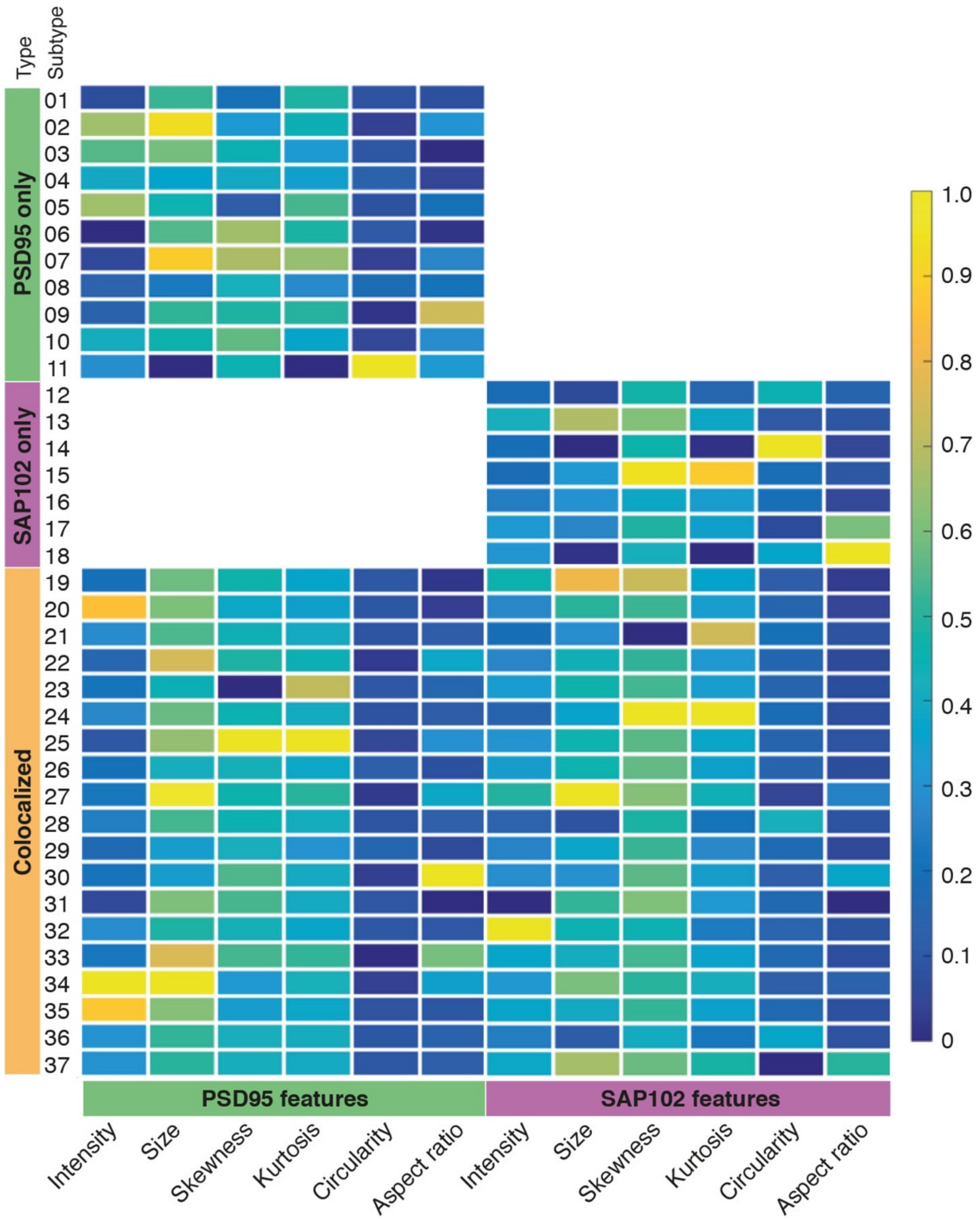


**Figure S9 related to Figure 3. Catalog of the 37 synapse subtypes identified and their parameters.**

Median value of the synaptome parameters (intensity, size, skewness, kurtosis, circularity, aspect ratio) for PSD95 (left side) and SAP102 (right side), for each of three types of synapses (PSD95-only; SAP102-only; Colocalized) that are further divided into 37 subtypes. Values are normalized for each parameter.

**Figure S10.**

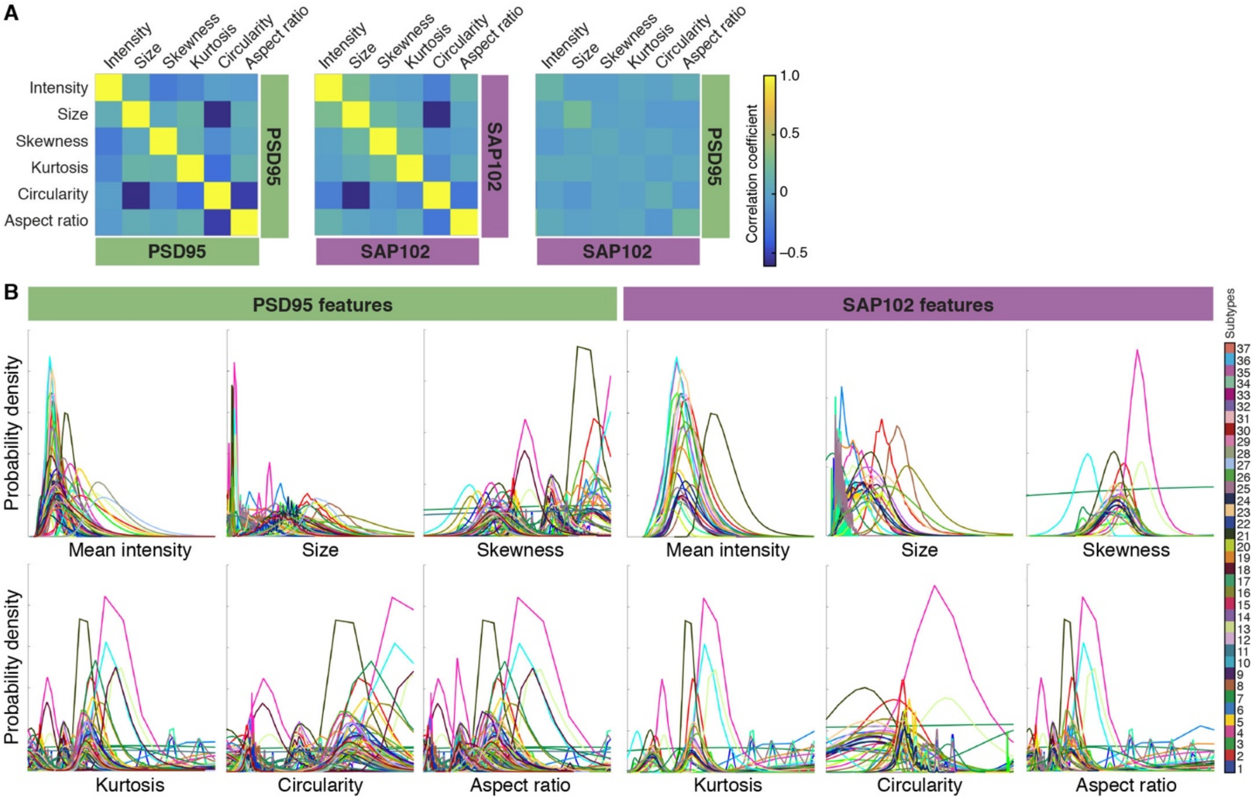


**Figure S10 related to Figure 3. Absence of correlation between the different parameters measured from individual puncta.**

A. The different synaptome parameters show low correlations at the level of individual synapses for PSD95 (left panel) and SAP102 (middle panel), and between PSD95 and SAP102 (right panel).

B. Probability density functions of each of the 12 synaptome parameters (left, PSD95 parameters; right, SAP102 parameters) for each 37 subtypes of synapses. Subtypes indicated by key.

**Figure S11.**

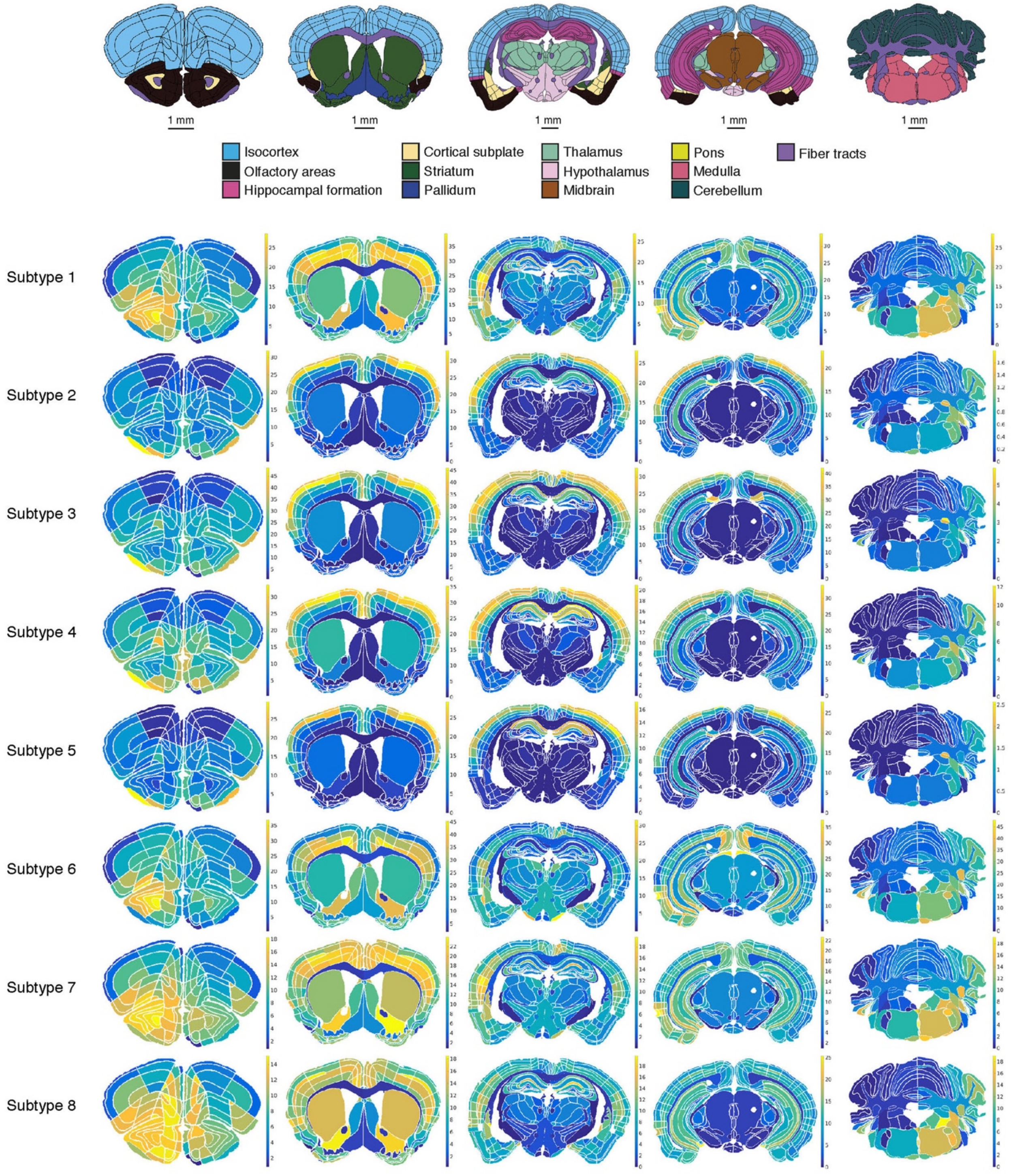

1 mm
Isocortex
Olfactory areas
Hippocampal formation
Cortical subplate
Striatum
Pallidum
Thalamus
Hypothalamus
Midbrain
Pons
Medulla
Cerebellum
Fiber tracts
Subtype 1
Subtype 2
Subtype 3
Subtype 4
Subtype 5
Subtype 6
Subtype 7
Subtype 8

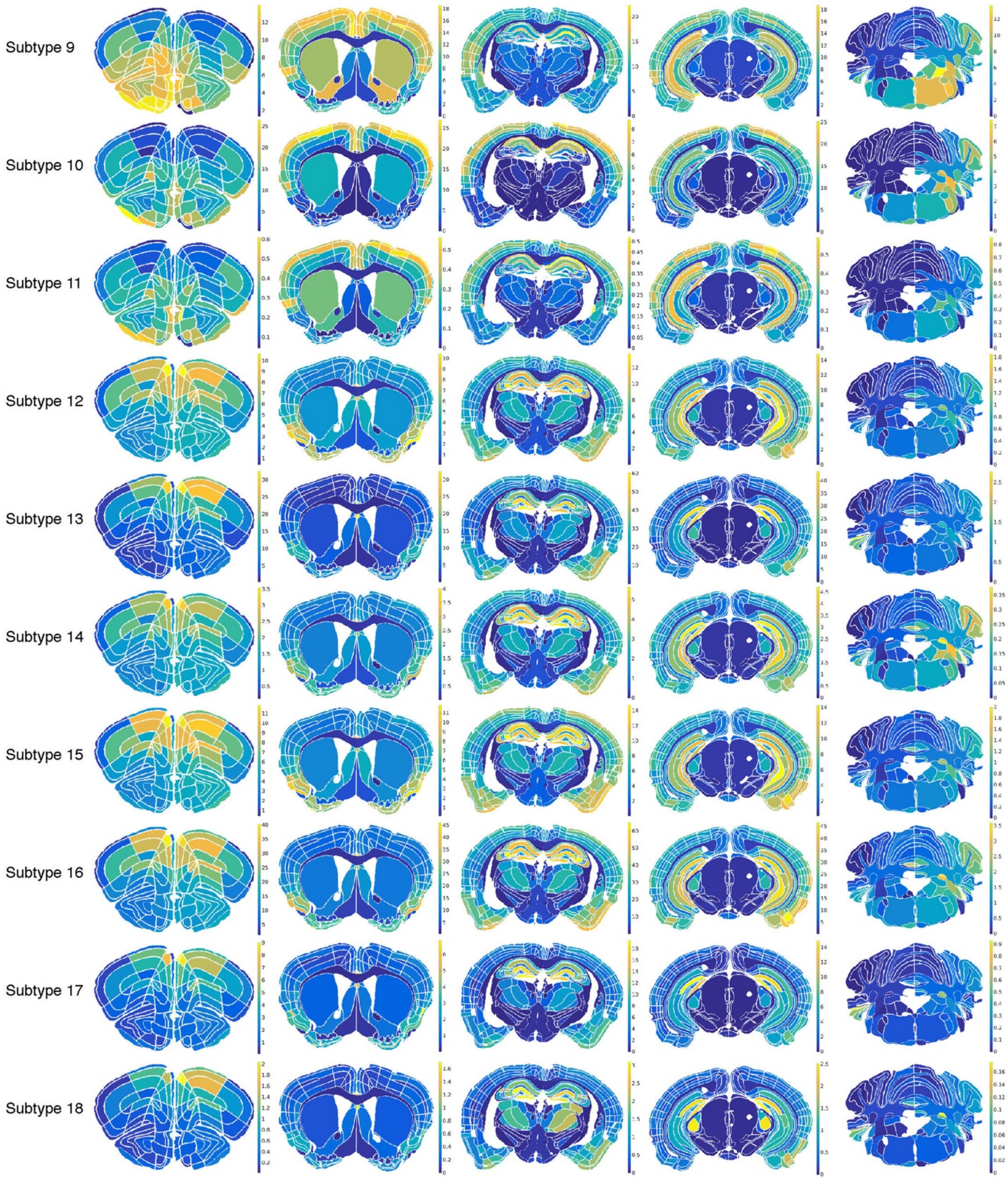
Subtype 9
Subtype 10
Subtype 11
Subtype 12
Subtype 13
Subtype 14
Subtype 15
Subtype 16
Subtype 17
Subtype 18

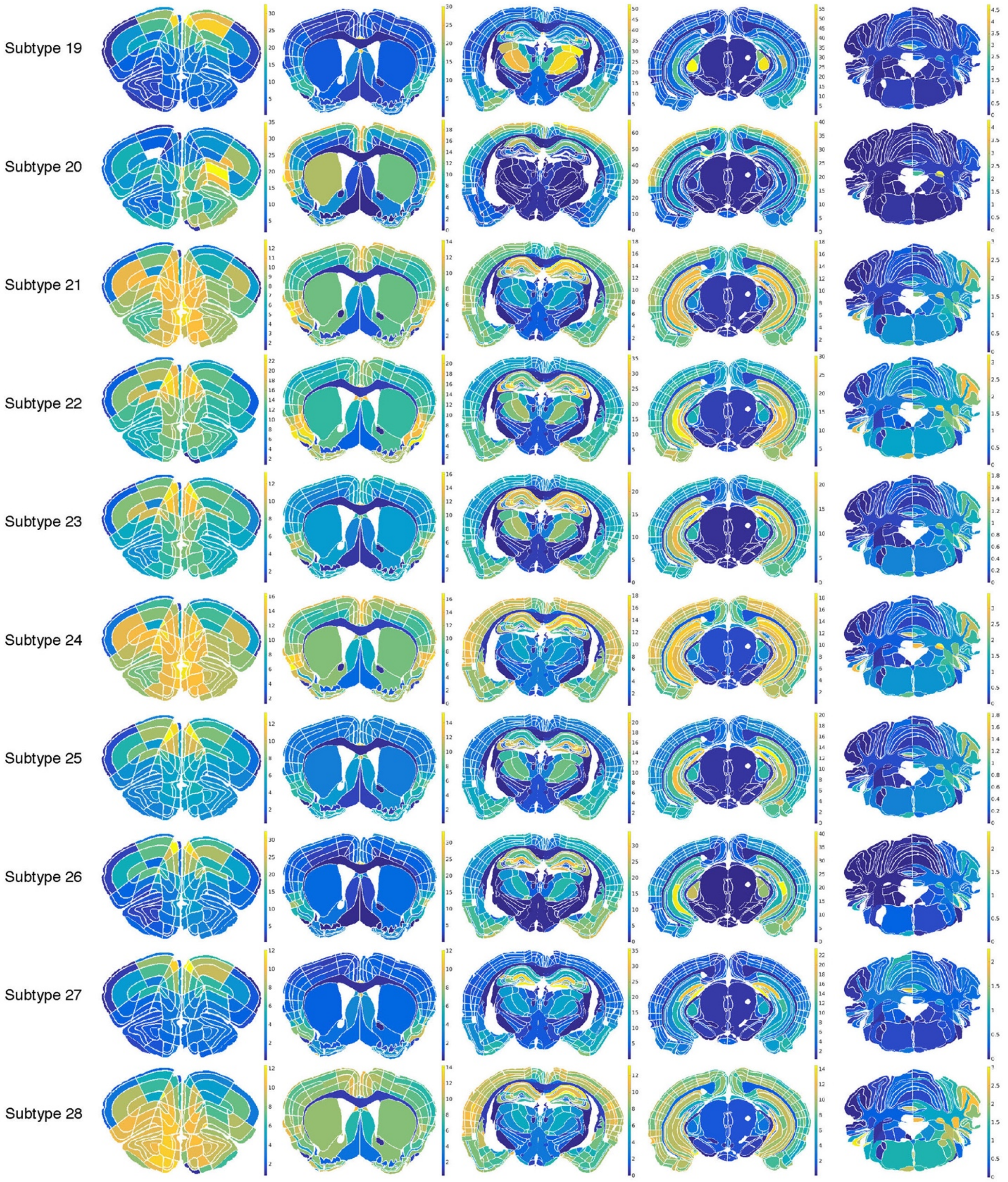
Subtype 19
Subtype 20
Subtype 21
Subtype 22
Subtype 23
Subtype 24
Subtype 25
Subtype 26
Subtype 27
Subtype 28

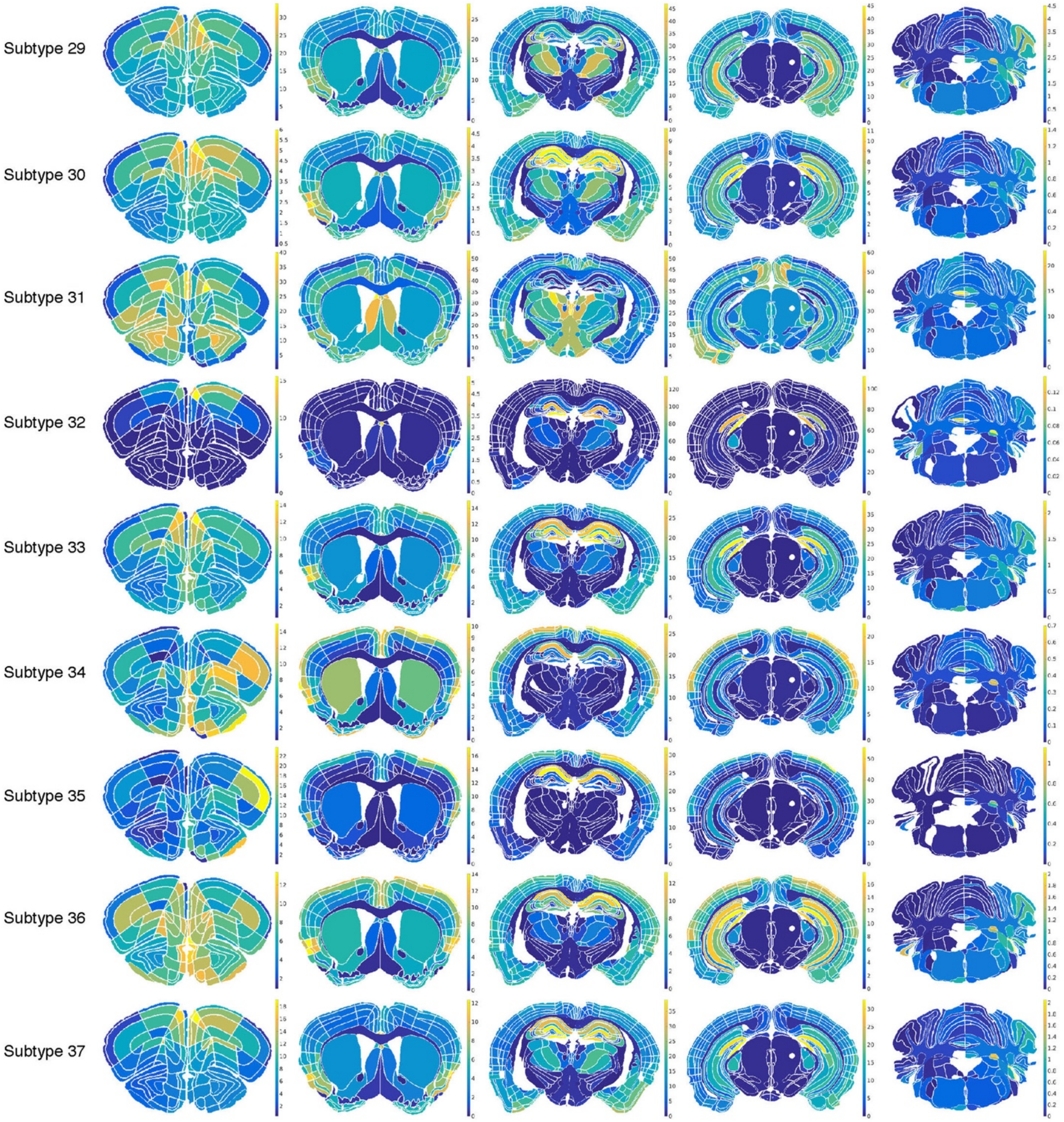


**Figure S11 related to Figure 3. Supervised synaptome maps of synaptic subtypes.** Median density distribution of each of the 37 synapse subtypes across the 5 coronal sections within defined anatomical subregions of the ARA. Subtypes #01–11 correspond to PSD95-only synapses (type 1), subtypes #12–18 correspond to SAP102-only synapses (type 2) and subtypes #19–37 correspond to colocalized synapses (type 3).

**Figure S12.**

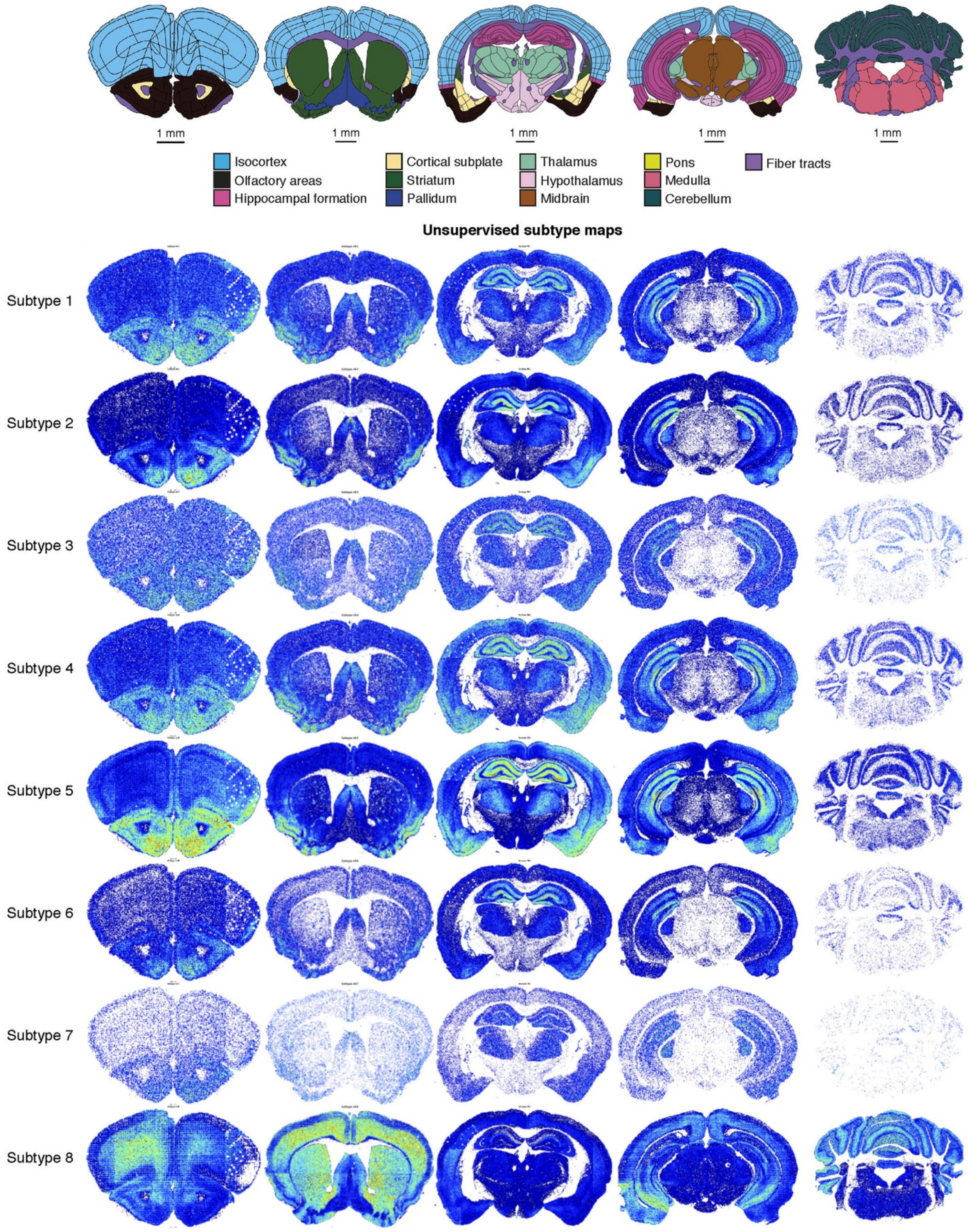

1 mm
Isocortex
Olfactory areas
Hippocampal formation
Cortical subplate
Striatum
Pallidum
Thalamus
Hypothalamus
Midbrain
Pons
Medulla
Cerebellum
Fiber tracts
Unsupervised subtype maps
Subtype 1
Subtype 2
Subtype 3
Subtype 4
Subtype 5
Subtype 6
Subtype 7
Subtype 8

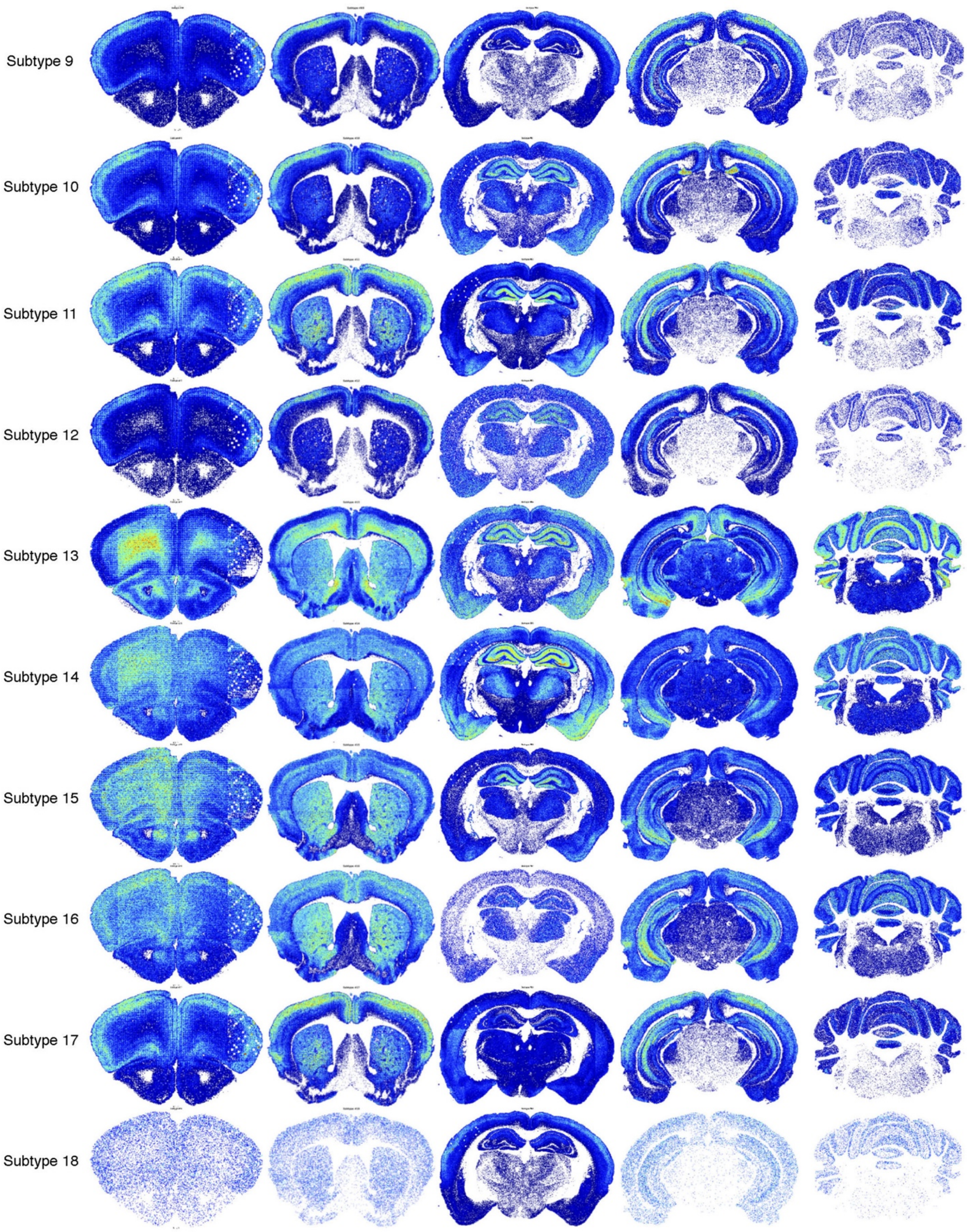

Subtype 9
Subtype 10
Subtype 11
Subtype 12
Subtype 13
Subtype 14
Subtype 15
Subtype 16
Subtype 17
Subtype 18

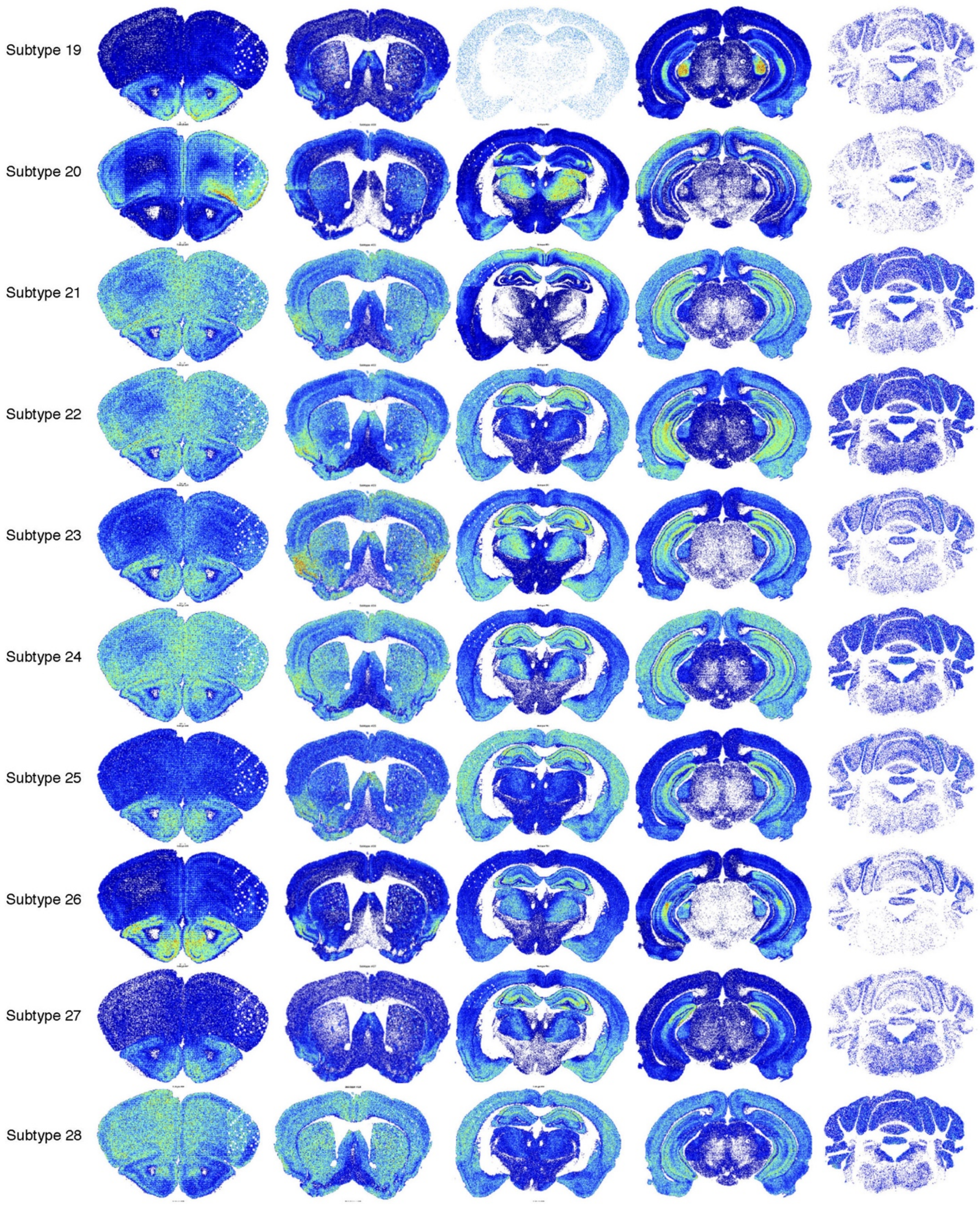
Subtype 19
Subtype 20
Subtype 21
Subtype 22
Subtype 23
Subtype 24
Subtype 25
Subtype 26
Subtype 27
Subtype 28

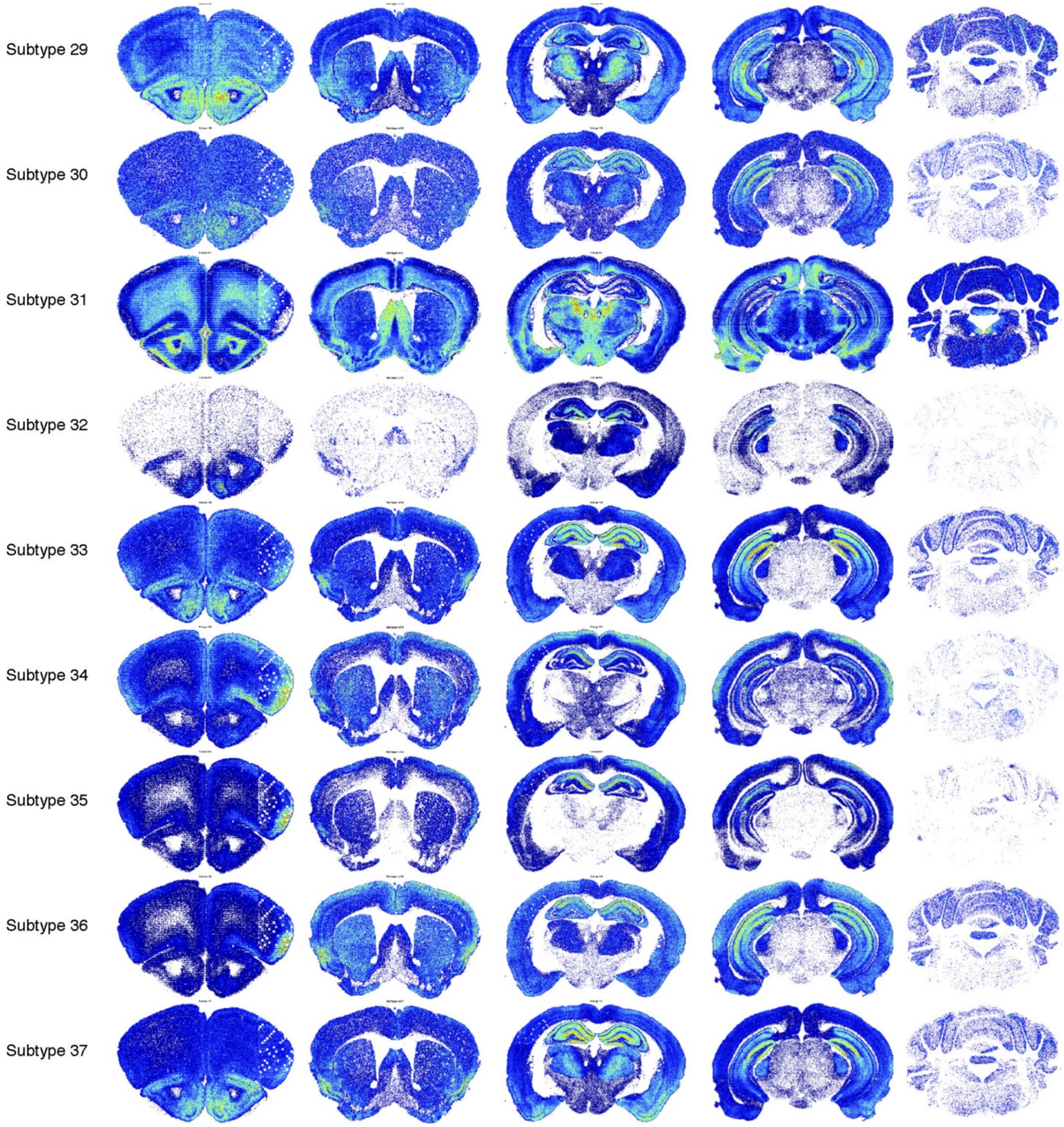


**Figure S12 related to Figure 3. Unsupervised synaptome maps of synaptic subtypes.**

Density distribution of each of the 37 synapse subtypes across the 5 coronal sections, independent of anatomical delineations. Subtypes #01–11 correspond to PSD95-only synapses (type 1), subtypes #12–18 correspond to SAP102-only synapses (type 2) and subtypes #19–37 correspond to colocalized synapses (type 3).

**Figure S13.**

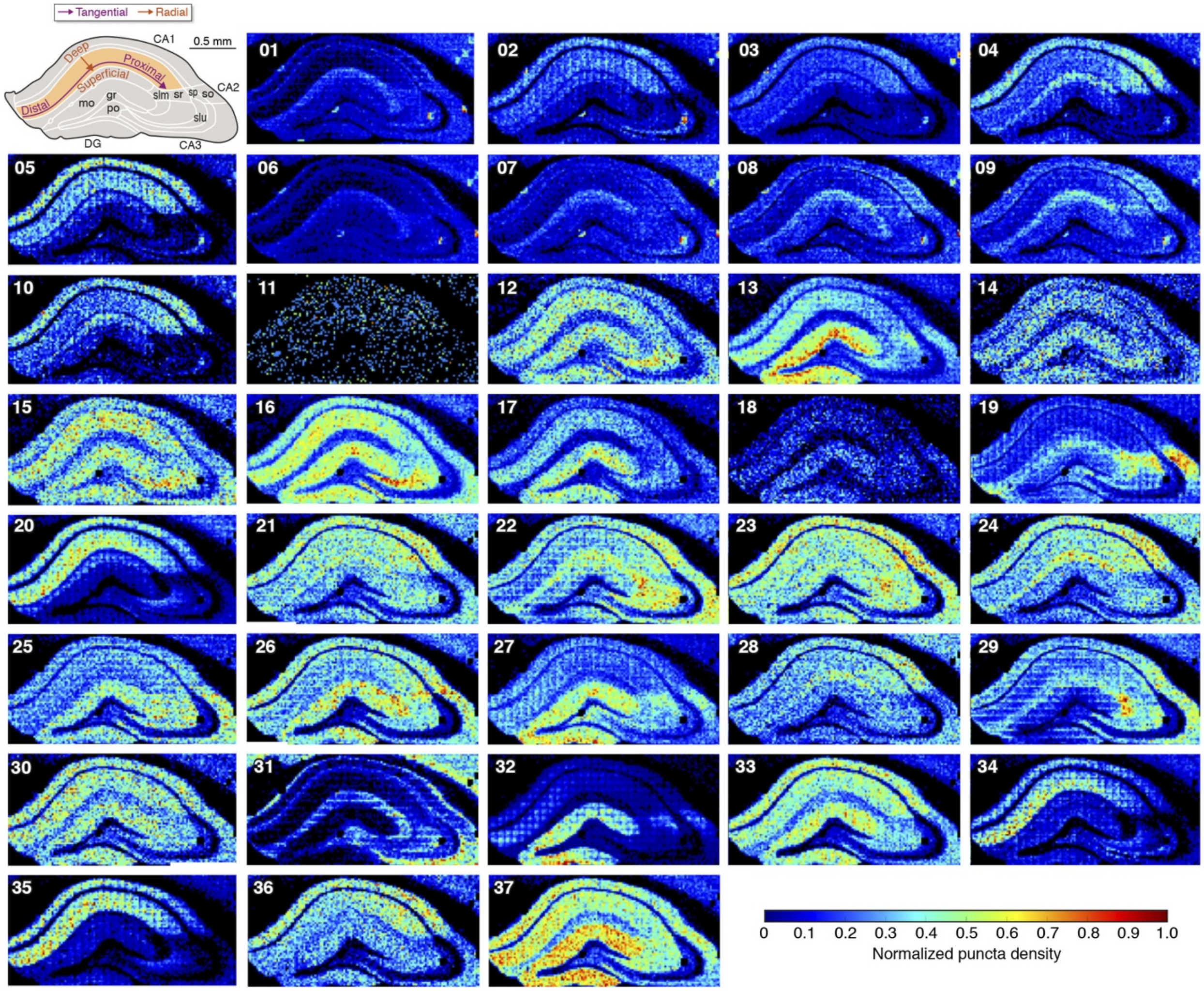


**Figure S13 related to Figure 3. Spatial distribution of synapse subtypes in the hippocampus.**

Density distribution of each of the 37 synapse subtypes in the dorsal hippocampus, independent of anatomical delineations. Top left panel shows the nomenclature of hippocampal subregions and gradients. Subtypes #01–11 correspond to PSD95-only synapses, subtypes #12–18 correspond to SAP102-only synapses and subtypes #19–37 correspond to colocalized synapses. Density values are normalized between 0 – 1 for the hippocampus.

**Figure S14.**

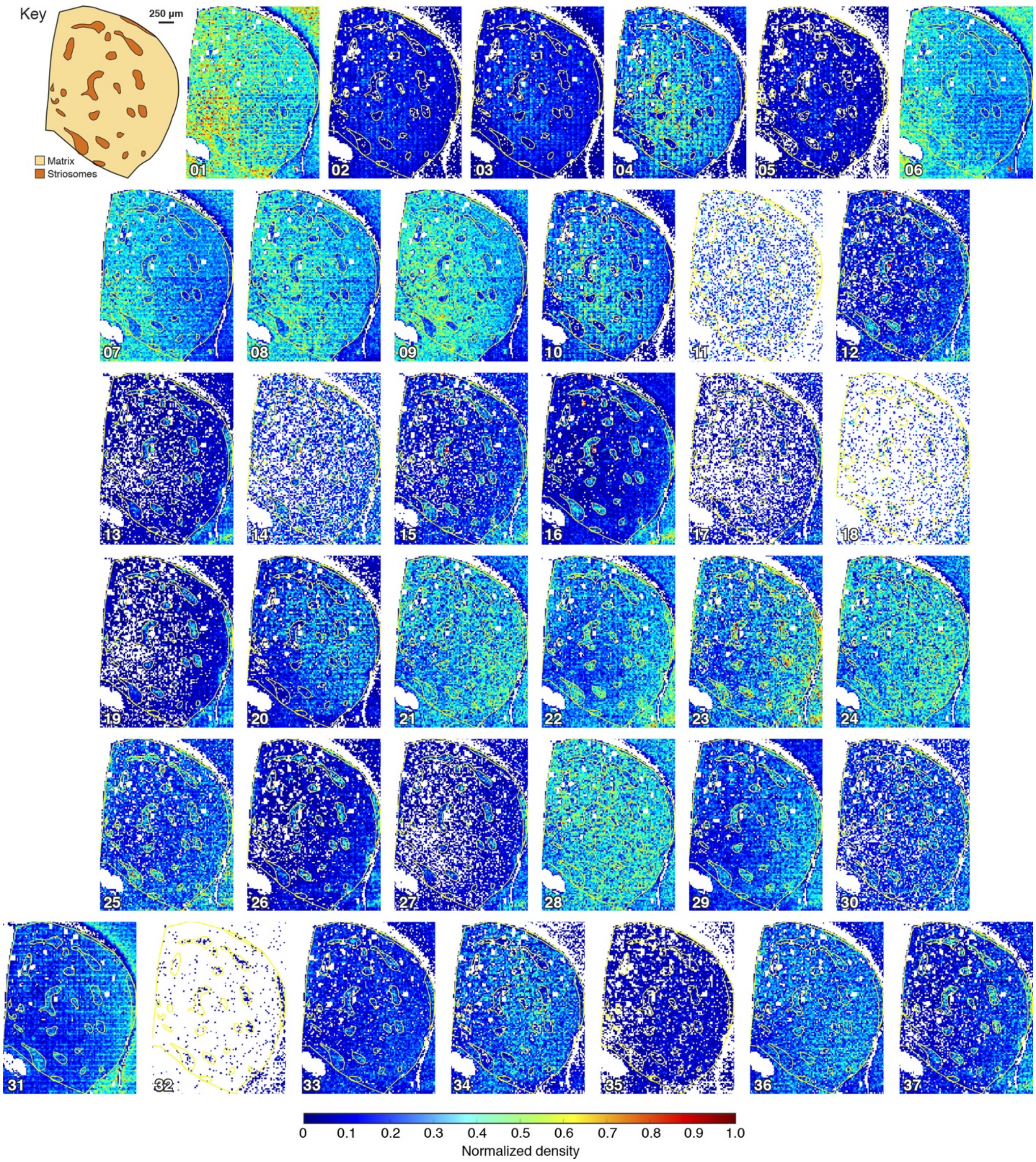


**Figure S14 related to Figure 3. Spatial distribution of synapse subtypes in the striatum.**

Density distribution of each of the 37 synapse subtypes in the caudate putamen (CP) independent of anatomical delineations. First panel shows boundaries between the

striosomes/matrix compartments and CP outlines, which are represented by yellow lines in all other panels. Subtypes #01–11 correspond to PSD95-only synapses, subtypes #12–18 correspond to SAP102-only synapses and subtypes #19-37 correspond to colocalized synapses. Density values are normalized between 0 – 1 for the striatum.

**Figure S15.**

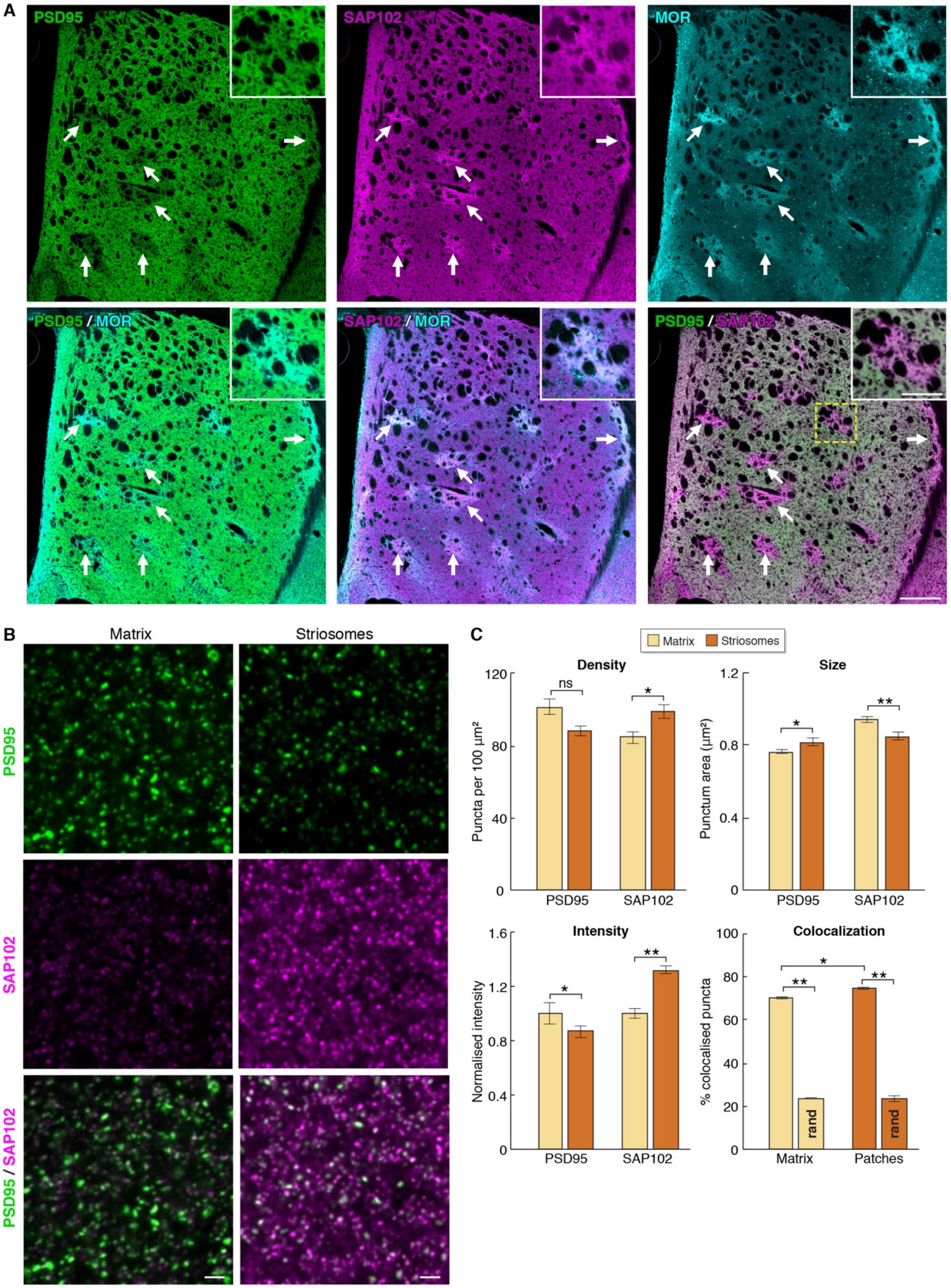
A
PSD95
SAP102
MOR
PSD95 / MOR
SAP102 / MOR
PSD95 / SAP102
B
Matrix
Striosomes
PSD95
SAP102
PSD95 / SAP102
C
Matrix
Striosomes
Density
Puncta per 100 µm²
ns
*
PSD95
SAP102
Size
Punctum area (µm²)
*
**
PSD95
SAP102
Intensity
Normalised intensity
*
**
PSD95
SAP102
Colocalization
% colocalised puncta
*
**
**
rand
rand
Matrix
Patches

**Figure S15 related to Figure 3. Distribution of PSD95 and SAP102 between striatal compartments.**

A. Low magnification images (20X) showing the expression pattern of PSD95 (green), SAP102 (magenta) and mu opioid receptor (MOR, cyan) in the striatum reveal that SAP102-rich/PSD95-poor patches match regions enriched in MOR, which correspond to striosomes (arrows). Insets are zoomed-in images of the striosome demarcated by the yellow box. Scale bars: 250 μm and 100 μm for insets.
B. High magnification images (100X) showing the differential expression of PSD95 (green) and SAP102 (magenta) at the level of individual synaptic puncta in matrix (left panels) and striosome compartments (right panels). Scale bar is 2 μm.
C. Quantification of punctum density, intensity, size and colocalization in the matrix compartment (yellow) and striosomes (orange). Significance was measured using paired t-tests between juxtaposed matrix and striosome compartments for N = 3 mice: * $p<0.05$, ** $p<0.01$. For colocalization, significance was also compared by paired *t*-test with a random (rand) control, using mismatched images between the two channels. Graphs are mean ± standard deviation.

**Figure S16.**

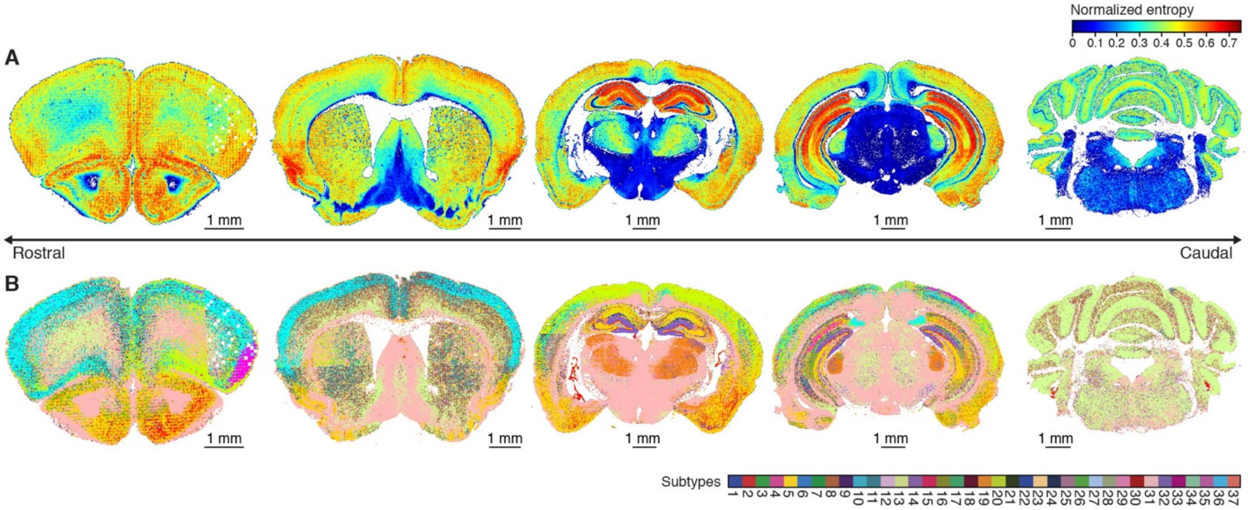


**Figure S16 related to Figure 3. Synaptome Diversity and Dominant subtype maps.**

A. Synaptome Diversity Map, as in Figure 3H, showing the spatial distribution of normalized Shannon information entropy per unit area (19.2μm × 19.2μm), which reflects the inequality in density between subtypes independent of anatomical delineations in the five coronal sections in 3H. Scale of normalized entropy indicated. Scale bars: 1 mm.

B. Synaptome Dominant Subtype Map, as in Figure 3E, showing the spatial distribution of the subtype with the highest density per area unit (19.2μm × 19.2μm) independent of anatomical delineations in the coronal sections. Subtype key indicated. Scale bars: 1 mm.

**Figure S17.**

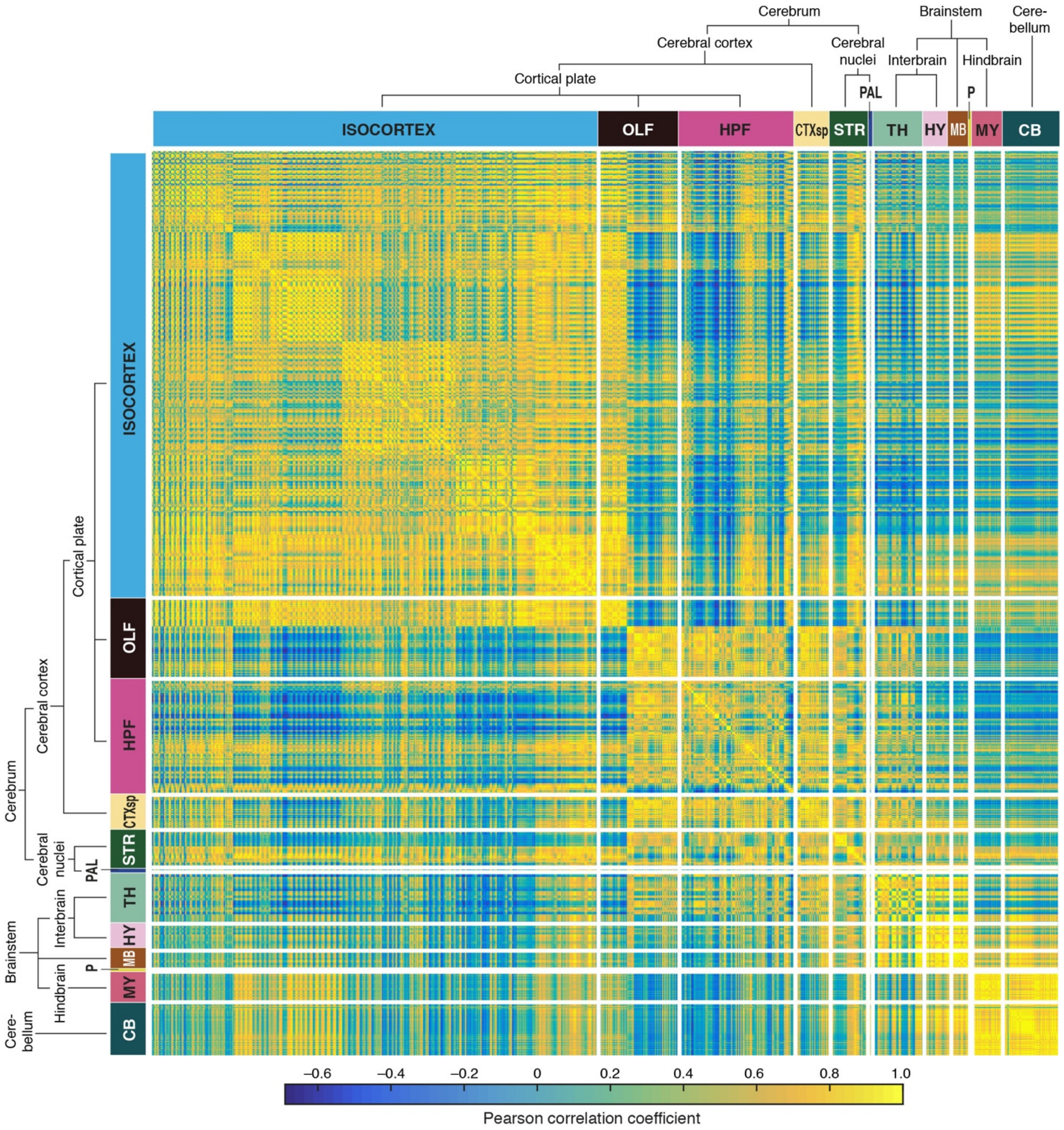


**Figure S17 related to Figure 3. Similarity matrix of subtype distribution between brain subregions.**

Matrix representing the level of similarity between pairs of subregions based on their density distributions of the 37 synapse subtypes. The similarity is calculated using the Pearson correlation coefficient, ranging from -1 to 1, where 1 indicates perfect similarity (yellow).

**Figure S18.**

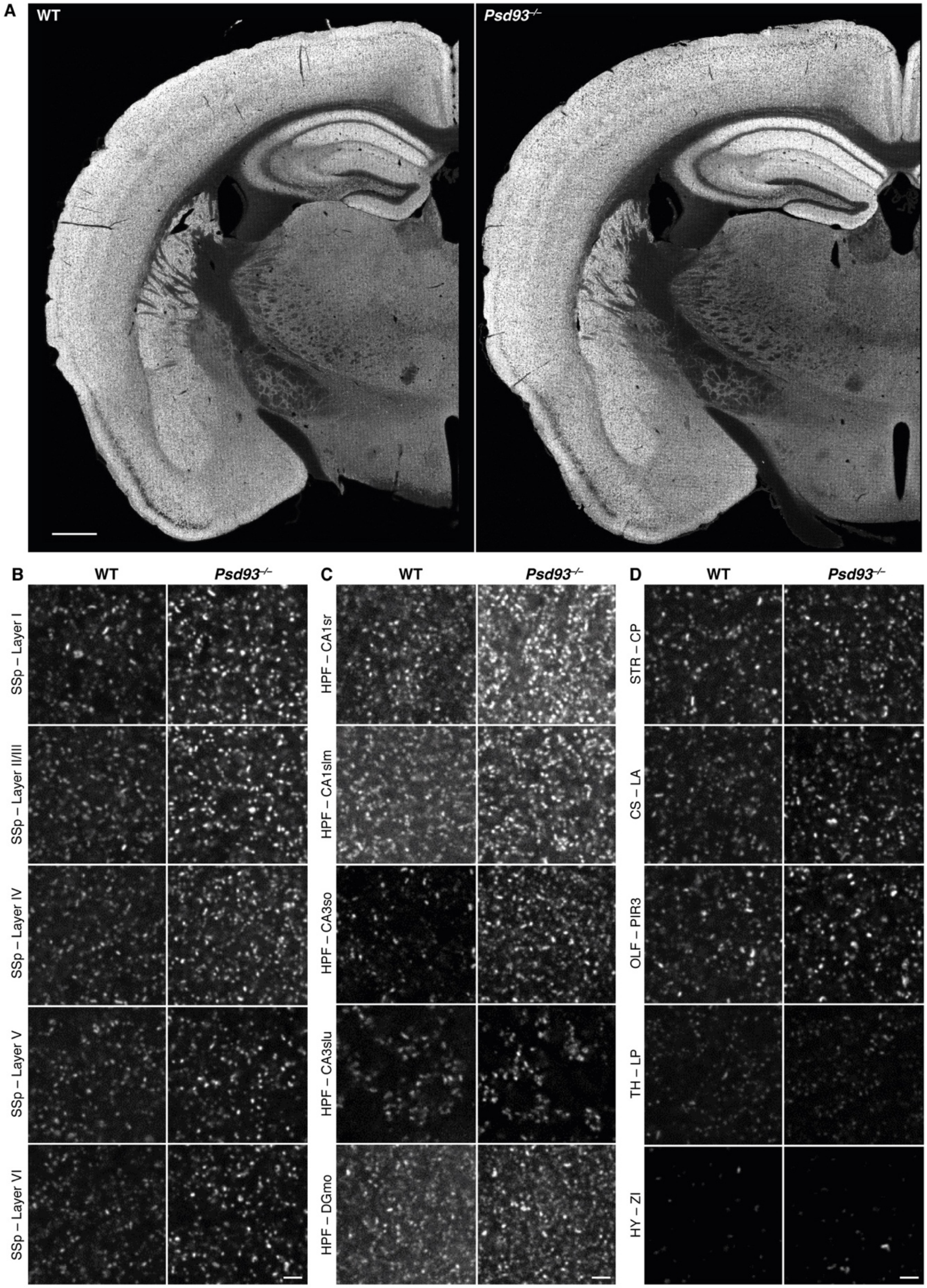
A
WT
Psd93−/−
B
WT
Psd93−/−
SSp – Layer I
SSp – Layer II/III
SSp – Layer IV
SSp – Layer V
SSp – Layer VI
C
WT
Psd93−/−
HPF – CA1sr
HPF – CA1slm
HPF – CA3so
HPF – CA3slu
HPF – DGmo
D
WT
Psd93−/−
STR – CP
CS – LA
OLF – PIR3
TH – LP
HY – ZI

**Figure S18 related to Figure 7. PSD95-eGFP expression pattern in wild-type and *Psd93*$^{-/-}$ mice.**

A. Low resolution images of PSD95-eGFP expression in wild-type (WT) and *Psd93*$^{-/-}$ mutant mice. Scale bar: 500 µm.

B. High magnification images (100X) of PSD95-eGFP expression in WT and *Psd93*$^{-/-}$ mice in the isocortex: primary somatosensory area (SSp), barrel field layers I to VI.

C. High magnification images (100X) of PSD95-eGFP expression in WT and *Psd93*$^{-/-}$ mice in the hippocampal formation (HPF): CA1 *stratum radiatum* (CA1sr), CA1 *stratum lacunosum-moleculare* (CA1slm), CA3 *stratum oriens* (CA3so), CA3 *stratum lucidum* (CA3slu) and *dentate gyrus* molecular layer (DGmo).

D. High magnification images (100X) of PSD95-eGFP expression in WT and *Psd93*$^{-/-}$ mice in other regions: the *Caudate Putamen* (CP) nucleus of the striatum (STR), the Lateral Amygdala nucleus (LA) of the cortical subplate (CS), layer 3 of the Piriform cortex (PIR3) of the olfactory areas (OLF), the Lateral Posterior nucleus (LP) of the thalamus (TH) and the *Zona Incerta* (ZI) of the hypothalamus (HY). Scale bars (B-D): 2 µm.

**Figure S19.**

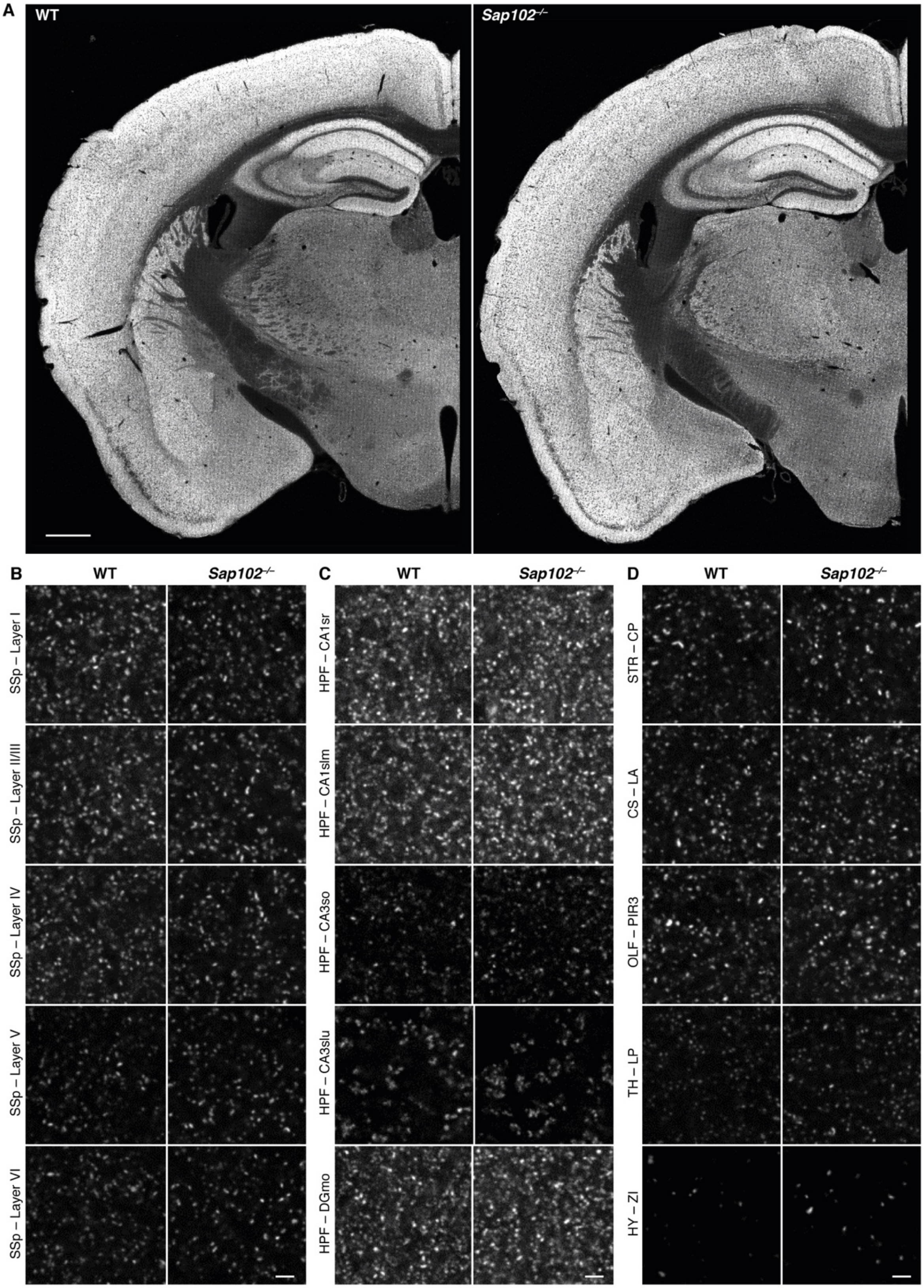
A
WT
*Sap102^{-/-}*
B
WT
*Sap102^{-/-}*
SSp – Layer I
SSp – Layer II/III
SSp – Layer IV
SSp – Layer V
SSp – Layer VI
C
WT
*Sap102^{-/-}*
HPF – CA1sr
HPF – CA1slm
HPF – CA3so
HPF – CA3slu
HPF – DGmo
D
WT
*Sap102^{-/-}*
STR – CP
CS – LA
OLF – PIR3
TH – LP
HY – ZI

**Figure S19 related to Figure 7. PSD95-eGFP expression pattern in wild-type and *Sap102*$^{-/-}$ mice.**

A. Low resolution image of PSD95-eGFP expression in wild-type (WT) and *Sap102*$^{-/-}$ mice. Scale bar: 500 μm.
B. High magnification pattern (100X) of PSD95-eGFP expression in WT and *Sap102*$^{-/-}$ mice in the isocortex: primary somatosensory area (SSp), barrel field layers I to VI.
C. High magnification pattern (100X) of PSD95 expression in WT and *Sap102*$^{-/-}$ mice in the hippocampal formation(HPF): CA1 *stratum radiatum* (CA1sr), CA1 *stratum lacunosum-moleculare* (CA1slm), CA3 *stratum oriens* (CA3so), CA3 *stratum lucidum* (CA3slu) and *dentate gyrus* molecular layer (DGmo).
D. High magnification pattern (100X) of PSD95 expression in WT and *Sap102*$^{-/-}$ mice in other regions: the *Caudate Putamen* (CP) nucleus of the striatum (STR), the Lateral Amygdalar nucleus (LA) of the cortical subplate (CS), layer 3 of the Piriform cortex (PIR3) of the olfactory areas (OLF), the Lateral Posterior nucleus (LP) of the thalamus (TH) and the *Zona Incerta* (ZI) of the hypothalamus (HY). Scale bars (B-D): 2 μm.

**Figure S20.**

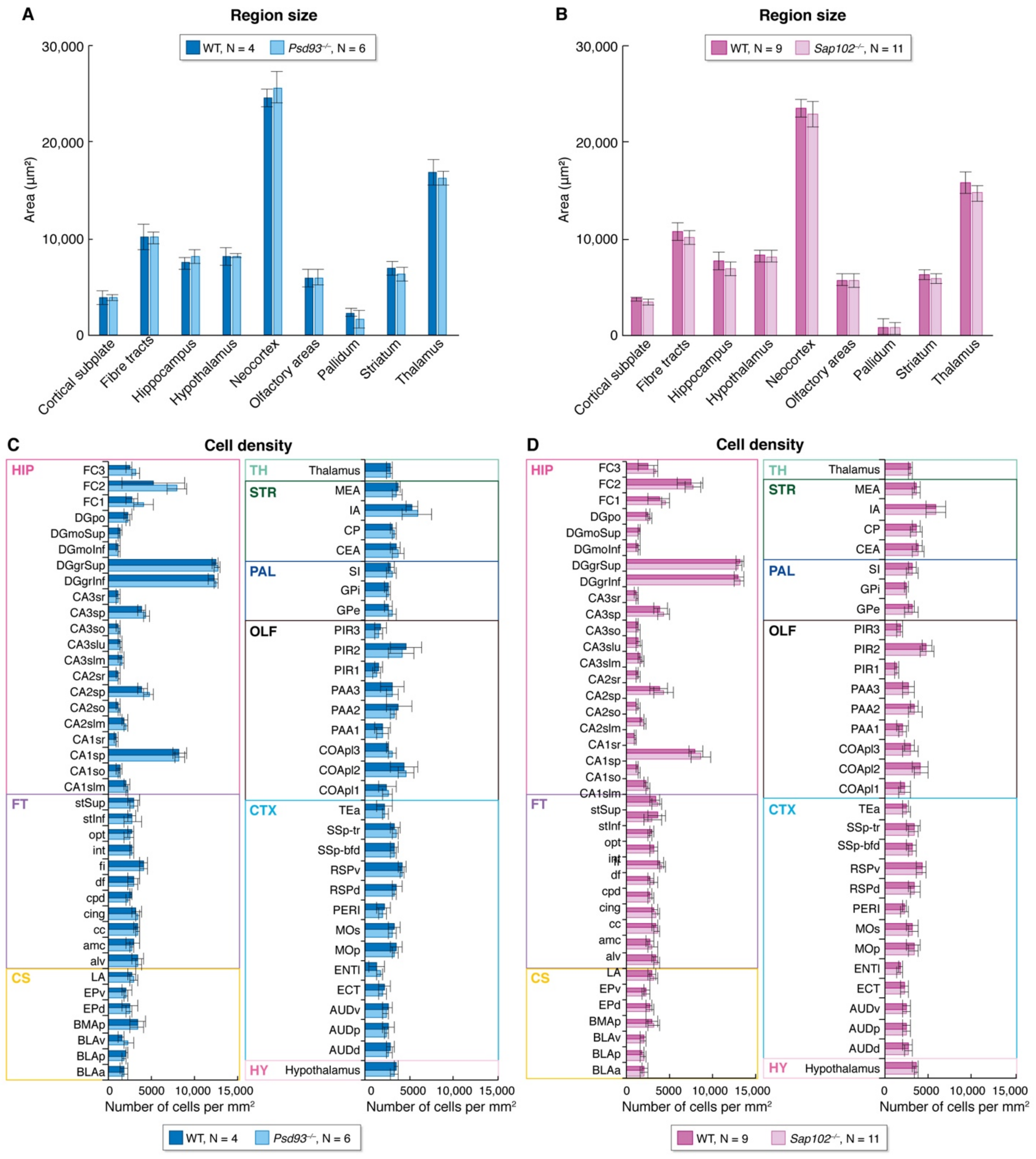


**Figure S20 related to Figure 7. Region size and cell density are unchanged in *Psd93*$^{-/-}$ and *Sap102*$^{-/-}$ mice.**

All graphs are mean ± standard deviation.

**A.** No significant difference in region size was observed in any of the overarching areas analyzed between WT mice (dark blue, N=4) and *Psd93*$^{-/-}$ mice (light blue, N=6).

**B.** No significant difference in region size was observed in any of the overarching areas analyzed between WT mice (dark pink, N=9) and *Sap102*$^{-/-}$ mice (light pink, N=11).

**C.** No significant difference in cell density was observed in any of the subregions of the hippocampus (HIP), fiber tracts (FT), cortical subplate (CS), thalamus (TH), striatum (STR), pallidum (PAL), olfactory areas (OLF), isocortex (CTX) nor hypothalamus (HY) between WT mice (dark blue, N=4) and *Psd93*$^{-/-}$ mice (light blue, N=6). Significance was assessed using the Bayesian estimation method with Benjamini-Hochberg correction. Subregions listed below.

**D.** No significant difference in cell density was observed in any of the subregions of the HIP, FT, CS, TH, STR, PAL, OLF, CTX nor HY between WT mice (dark pink, N=9) and *Sap102*$^{-/-}$ mice (light pink, N=11). Significance was assessed using the Bayesian estimation method with Benjamini-Hochberg correction. Subregions listed below.

Subregion abbreviations: alv: alveus; amc: amygdalar capsule; AUDd: Auditory area, dorsal part; AUDp: Auditory area, primary part; AUDv: Auditory area, ventral part; BLAa: Basolateral Amygdalar nucleus, anterior part; BLAp: Basolateral Amygdalar nucleus, posterior part; BLAv: Basolateral Amygdalar nucleus, ventral part; BMAp: Basomedial Amygdalar nucleus, posterior part; CA1slm: Cornu Ammonis 1, stratum lacunosum-moleculare; CA1so: Cornu Ammonis 1, stratum oriens; CA1sp: Cornu Ammonis 1, stratum pyramidale; CA1sr: Cornu Ammonis 1, stratum radiatum; CA2slm: Cornu Ammonis 2, stratum lacunosum-moleculare; CA2so: Cornu Ammonis 2, stratum oriens; CA2sp: Cornu Ammonis 2, stratum pyramidale; CA2sr: Cornu Ammonis 2, stratum radiatum; CA3slm: Cornu Ammonis 3, stratum lacunosum-moleculare; CA3slu: Cornu Ammonis 3, stratum lucidum; CA3so: Cornu Ammonis 3, stratum oriens; CA3sp: Cornu Ammonis 3, stratum pyramidale; CA3sr: Cornu Ammonis 3, stratum radiatum; cc: corpus callosum; CEA: Central Amygdalar nucleus; cing: cingulum bundle; COApl1: Cortical Amygdalar area, posterior part, lateral zone, layer 1; COApl2: Cortical Amygdalar area, posterior part, lateral zone, layer 2; COApl3: Cortical Amygdalar area, posterior part, lateral zone, layer 3; CP: Caudate Putamen; cpd: cerebral peduncle; df: dorsal fornix; DGgrInf: Dentate Gyrus, granular layer, inferior blade; DGgrSup: Dentate Gyrus, granular layer, superior blade; DGmoInf: Dentate Gyrus, molecular layer, inferior blade;

DGmoSup: Dentate Gyrus, molecular layer, superior blade; DGpo: Dentate Gyrus, polymorphic cell layer; ECT: Ectorhinal area; ENTl: Entorhinal area, lateral part; EPd: Endopiriform nucleus, dorsal part; EPv: Endopiriform nucleus, ventral part; FC1: Fasciola Cinerea, layer 1; FC2: Fasciola Cinerea, layer 2; FC3: Fasciola Cinerea, layer 3; fi: fimbria; GPe: Globus Pallidus, external segment; GPi: Globus Pallidus, internal segment; IA: Intercalated Amygdalar nucleus; int: internal capsule; LA: Lateral Amygdalar nucleus; MEA: Medial Amygdalar nucleus; MOp: Primary motor area; MOs: secondary Motor area; opt: optic tract; PAA1: Piriform-Amygdalar Area, molecular layer; PAA2: Piriform-Amygdalar Area, pyramidal layer; PAA3: Piriform-Amygdalar Area, polymorph layer; PERI: Perirhinal area; PIR1: Piriform area, layer 1; PIR2: Piriform area, layer 2; PIR3: Piriform area, layer 3; RSPd: Retrosplenial area, dorsal part; RSPv: Retrosplenial area, ventral part; SI: primary Somatosensory cortex; SSp-bfd: primary Somatosensory area, barrel field; SSp-tr: primary Somatosensory area, trunk; stInf: stria terminalis, inferior part; stSup: stria terminalis, superior part; TEa: Temporal association areas.
